# Role of disulfidptosis in colorectal adenocarcinoma: implications for prognosis and immunity

**DOI:** 10.3389/fimmu.2024.1409149

**Published:** 2024-09-27

**Authors:** Ruanruan Yang, Chunxiao Lai, Luji Huang, Feng Li, Weiqi Peng, Meiyan Wu, Jinge Xin, Yan Lu, Manzhao Ouyang, Yang Bai, Haoqiang Lei, Shunhui He, Yu Lin

**Affiliations:** ^1^ Department of Gastroenterology, Baiyun Branch, Nanfang Hospital, Southern Medical University, Guangzhou, China; ^2^ Department of Gastrointestinal Surgery, Shunde Hospital, Southern Medical University, Foshan, China; ^3^ Department of Gastroenterology, Shenzhen Hospital of Beijing University of Chinese Medicine (Longgang), Shenzhen, China; ^4^ Department of Good Clinical Practice (GCP), Shunde Hospital, Southern Medical University, Foshan, China; ^5^ Huangpu People’s Hospital of Zhongshan, Zhongshan, China; ^6^ Department of Gastroenterology, Shunde Hospital, Southern Medical University, Foshan, China

**Keywords:** disulfidptosis, colorectal cancer, prognosis, drug sensitivity, classification

## Abstract

**Background:**

Recent research has found a new way of cell death: disulfidptosis. Under glucose starvation, abnormal accumulation of disulfide molecules such as Cystine in Solute Carrier Family 7 Member 11 (SLC7A11) overexpression cells induced disulfide stress to trigger cell death. The research on disulfidptosis is still in its early stages, and its role in the occurrence and development of colorectal malignancies is still unclear.

**Method:**

In this study, we employed bioinformatics methods to analyze the expression and mutation characteristics of disulfidptosis-related genes (DRGs) in colorectal cancer. Consensus clustering analysis was used to identify molecular subtypes of Colorectal Adenocarcinoma (COAD) associated with disulfidptosis. The biological behaviors between subtypes were analyzed to explore the impact of disulfidptosis on the tumor microenvironment. Constructing and validating a prognostic risk model for COAD using diverse data. The influence of key genes on prognosis was evaluated through SHapley Additive exPlanations (SHAP) analysis, and the predictive capability of the model was assessed using Overall Survival analysis, Area Under Curve and risk curves. The immunological status of different patients and the prediction of drug treatment response were determined through immune cell infiltration, TMB, MSI status, and drug sensitivity analysis. Single-cell analysis was employed to explore the expression of genes at the cellular level, and finally validated the expression of key genes in clinical samples.

**Result:**

By integrating the public data from two platforms, we identified 2 colorectal cancer subtypes related to DRGs. Ultimately, we established a prognosis risk model for COAD using 7 genes (FABA4+GIPC2+EGR3+HOXC6+CCL11+CXCL10+ITLN1). SHAP analysis can further explained the positive or negative impact of gene expression on prognosis. By dividing patients into high-risk and low-risk groups, we found that patients in the high-risk group had poorer prognosis, higher TMB, and a higher proportion of MSI-H and MSI-L statuses. We also predicted that drugs such as 5-Fluorouracil, Oxaliplatin, Gefitinib, and Sorafenib would be more effective in low-risk patients, while drugs like Luminesib and Staurosporine would be more effective in high-risk patients. Single-cell analysis revealed that these 7 genes not only differ at the level of immune cells but also in epithelial cells, fibroblasts, and myofibroblasts, among other cell types. Finally, the expression of these key genes was verified in clinical samples, with consistent results.

**Conclusions:**

Our research findings provide evidence for the role of disulfidptosis in COAD and offer new insights for personalized and precise treatment of COAD.

## 1 Introduction

Colorectal cancer (CRC) is the third most common malignant tumor in the world, with a mortality rate ranking second globally (1).Colon adenocarcinoma (COAD) accounts for 90% of cases (2), and it is estimated that there will be 3.2 million new cases of COAD worldwide by 2040 (3). Early-stage COAD patients who undergo radical resection can achieve a 5-year survival rate of up to 90%. However, most clinically diagnosed COAD patients are in the advanced clinical stage, and despite new treatment options such as targeted therapy and immunotherapy, the 5-year survival rate for average advanced-stage COAD patients is still not more than 15% due to the complexity of colorectal cancer and various factors such as immune escape and drug resistance (4). Therefore, it is important to explor specific molecular markers related to the prognosis of colorectal adenocarcinoma patients, identifying different subtypes of colorectal adenocarcinoma, and guiding treatment using bioinformatics methods.

A novel and interesting form of cell death that has recently gained attention is called disulfidptosis, characterized by disulfide stress. The occurrence of disulfidptosis is triggered by the inhibition of NADPH synthesis under glucose starvation conditions, which induces high expression of SLC7A11, leading to an increased influx of cysteine into the cell, resulting in abnormal accumulation of intracellular disulfide bonds and subsequent protein misfolding. Disulfidptosis does not belong to any known type of cell death, such as ferroptosis, apoptosis, or pyroptosis, and it cannot be inhibited by conventional drugs used to suppress cell death nor prevented by knocking out key genes for ferroptosis/apoptosis. Reducing agents for disulfide stress, such as dithiothreitol, β-mercaptoethanol, and Tris(2-carboxyethyl) phosphine, can completely inhibit glucose starvation-induced cell death in SLC7A11 high cells. In addition, thiol oxidants (diamine and diethyl maleate) promote cell death in SLC7A11high cells under glucose starvation and lead to a sharp accumulation of disulfide molecules within cells (5).

Some studies have provided insights into the potential role of disulfidptosis in cancer biology. For example, disulfidptosis is associated with cellular oxidative stress, where cancer cells under oxidative stress conditions lead to the accumulation of disulfide bonds, a redox state closely related to the survival and proliferation of cancer cells (6) Furthermore, disulfidptosis, by affecting the structure of the actin cytoskeleton, increases memne permeability and disrupts the structural integrity of the cell memne, affecting cancer cell migration, invasion, and may also induce apoptosis and other forms of programmed cell death (7). Disulfidptosis may regulate the activity and function of immune cells, influencing the tumor immune microenvironment, and thus affecting the outcome of tumor patients. These findings emphasize the importance of further exploring the mechanisms of disulfidptosis in various cancers, including colorectal cancer. The study by Liu et al. also proposes strategies for targeting disulfidptosis in cancer treatment (5).

Despite the growing body of research on disulfidptosis, a comprehensive analysis of the expression patterns, potential molecular pathways, clinical significance, and immune correlation of disulfidptosis-related genes in COAD is still required. This study integrated two sets of COAD data from TCGA and GEO using bioinformatics analysis methods, investigated the gene expression and mutation characteristics of DRGs in COAD, identified key genes and subtypes, and subsequently constructed a risk prognosis model. The potential clinical applicability and value of the model for guiding individualized treatment were confirmed through immune infiltration analysis and drug sensitivity analysis. The aim is to contribute to the development of new treatment strategies and the improvement of prognosis for COAD patients.

## 2 Materials and methods

### 2.1 Data sources and processing

RNA-seq data and clinical information for colorectal cancer patients were collected from two sources: GSE39582(http://www.ncbi.nlm.nih.gov/geo/) and TCGA-COAD (https://cancergenome.nih.gov/). A total of 969 patients were included in this study. The clinical variables included age, sex, TNM stage, tumor grade, follow-up time, and survival status. Additionally, downloading simple nucleotide variation (SNV) data for TCGA-COAD patients from the UCSC Xena database (http://xena.ucsc.edu/).The FPKM values were transformed into transcripts per kilobase million (TPM), which were treated as comparable to transcripts obtained from the GEO microarray. The measurement of gene expression profiles was achieved by utilizing TPM estimation, followed by log2-based transformation. We utilized Strawberry Perl (version 5.26) to perform ID conversion on the two datasets. Furthermore, we combined and performed batch correction on mRNA expression data from the four datasets using the “limma” and “sva” packages, respectively (8). The twenty-four genes are disulfidptosis-related genes(DRGs)retrieved from the currently available publications, and they include SLC7A11, GYS1, NDUFS1, NDUFA11, NUBPL, NCKAP1, LRPPRC, SLC3A2, RPN1, ACTN4, ACTB, CD2AP, CAPZB, DSTN, FLNA, FLNB, INF2, IQGAP1, MYH10, MYL6, MYH9, PDLIM1, TLN1, and OXSM (5, 9). We downloaded ‘GDSC2_Res.rds’ and ‘GDSC2_Expr.rds’ from the Cancer Cell Line Encyclopedia database (https://sites.broadinstitute.org/ccle/) for drug sensitivity analysis (10).

### 2.2 Consensus clustering analysis of PRGs

The R package “Consensus ClusterPlus” was used for consensus unsupervised clustering analysis to classify patients into different DRG-related molecular subtypes based on the expression of DRGs (11). The clustering analysis was performed using the “PAM” algorithm with Euclidean distance as the distance measure. Eighty percent of the samples were randomly sampled and repeated 1000 times. The optimal K value was confirmed through the proportion of fuzzy clustering (PAC) and cumulative distribution function (CDF), and K was taken as the number of molecular subtypes.

### 2.3 The relationship between molecular subtypes and clinical characteristics and prognosis of colorectal cancer

We compared the clinical features (including age, gender, TNM stage) among different molecular subtypes using the ‘pheatmap’ package for visualization. Survival time and status were integrated from two datasets (946 patients), and the overall survival (OS) differences among different subtypes were evaluated based on the Kaplan-Meier method. The ‘Limma’ package in R language was used to screen for differentially expressed genes (DEGs) between different molecular subtypes in terms of double sulfur death.

### 2.4 Correlation enrichment analysis

We used the ‘c5.go.symbols.gmt’ and ‘c2.cp.kegg.Hs.symbols’ files from the MsigDB database (https://www.gsea-msigdb.org/gsea/msigdb) (12) to perform gene set variation analysis (GSVA) and gene set enrichment analysis (GSEA) (13),analyzing the biological functional differences between high- and low-risk populations and the biological functional differences associated with DRGs. To explore the potential biological functions of DEGs, Gene Ontology (GO) (14) and Kyoto Encyclopedia of Genes and Genomes (KEGG) analyses (15) were performed using the ‘cluster Profiler’ package (16) with a p-value<0.05 considered statistically significant.

### 2.5 Construction of disulfidptosis-related prognostic signature in colorectal cancer

Firstly, using the ‘limma’ package, a LASSO-univariate Cox analysis was conducted on the differentially expressed genes (DEGs) to screen for genes associated with prognosis as subsequent genes^15^. Based on the expression levels of the candidate genes, cluster analysis was performed to classify patients into different subtypes for further analysis. Subsequently, multivariate Cox analysis was conducted to further screen the candidate genes and obtain the target gene. Remove samples with missing expression of target genes, and finally include 920 patients to construct the model. The patients were divided into training set and validation set at a ratio of 1:1. The risk score was calculated using the following formula:

score=“∑ (Expi∗coefi)” , where Σ denotes the sum from i=1 to N, and N is the number of selected genes.

The median cut-off value of the risk score was used to divide patients into high and low-risk groups. The ‘pheatmap’ package in R was used to draw risk curves and heatmap, and the ‘survival’ package was used to draw survival curves and ROC curves to evaluate the clinical predictive value of the model. We also performed a drill-down analysis on the impact of each gene on the prognosis model using SHAP value dependence analysis based on single features. SHAP values can quantify the impact of the expression levels of each gene on the patient’s prognosis, thereby making this model more effective in guiding medical practice and medical diagnosis (16). We use the “rms” package to construct a nomogram, which is used to predict the OS of patients at 1 year, 3 years, and 5 years.

### 2.6 The analysis of tumor microenvironment and immune-related factors

By downloading the source code “CIBERSORT.R” and reference data files from the CIBERSORT website (https://cibersort.stanford.edu/) and combining the gene expression data from two datasets, CIBERSORT R was used to analyze the samples, resulting in an input sample file “CIBERSORT-Results.txt” (showing the infiltration of 22 types of immune cells in each tumor sample). Using the “limma” package, we investigated the differences in 22 immune-infiltrating cells between high and low-risk groups (17). Additionally, based on the gene matrix, the “estimate” package was used to evaluate the stromal, immune, and tumor purity of the tumor tissue (17, 18). Finally, we utilized TISCH (http://tisch.comp-genomics.org/) to investigate the impact of 7 prognostic markers on the tumor microenvironment at the single-cell level.

### 2.7 Mutation data processing and tumor mutation burden

Using SNV data from 359 Colorectal cancer patients in the TCGA database, the tumor mutation burden (TMB) was calculated for each patient, where TMB = the number of non-synonymous mutations/exome chip size (approximately 38Mb) (19, 20). A Perl script was used to obtain the number of non-synonymous mutations in the sequencing data for each patient, which was then adjusted using the above formula. The “GenVisR” package was used to draw gene mutation waterfall plots for the high and low-risk groups separately. The “survival” package was used to analyze the relationship between the high and low-risk groups, TMB levels, and survival prognosis.

### 2.8 Drug sensitivity analysis

Using the ‘oncoPredict’ package, sensitivity scores for 198 drugs were calculated for 909 patients, allowing for a comparison of the therapeutic effects of targeted biologics between the high and low-risk groups.

### 2.9 Statistical analyses

All data analyses were performed using the following software: R language (version 4.3.0) and Strawberry Perl (version 5.30.0). A p-value less than 0.05 was considered statistically significant.

### 2.10 Analysis of quantitative reverse transcription polymerase chain reaction

Both colorectal cancer and adjacent non-cancerous tissues used in this study were derived from patients with colorectal cancer after surgery in the Shunde Hospital, during 2022-2023. The acquisition and use of clinical samples used in this study were approved by the Medical Ethics Committee of Shunde Hospital of Southern Medical University (KYL20220125).

We extracted RNA from the specimen using a TRlzol reagent (Ambion, USA) and then reverse-transcribed it into cDNA using a quantitative reverse transcription kit (Promega, USA). Quantitative PCR (qPCR)is a technique for measuring DNA content in sample in real time. Real-time fluorescence quantitative qPCR assay was performed with the help of SYBR-Green (Vazyme, China) and expression levels were standardized to -actin levels. The primers are shown in **Supplementary Table S5**.

## 3 Results

### 3.1 Basic information of colorectal cancer patients

This study included two datasets with a total of 969 patients with rectal cancer (**Table 1**). Clinical data on survival time and survival status were available for all patients (**Table 2**).

**Table 1:** Information of two datasets.

Datasets	Platform	Country	Numbers of patients
GSE39582	GPL570	French	585
TCGA	Illumina HiSeq	US	384

**Table 2:** General information of patients in two datasets.

	GSE26712	TCGA
Age(mean ± SD)	71 ± 13.3	67.0 ± 12.8
Gender(Femla/Male)	256/310	180/205
Stage(n,%)		
I	Na	66
II	Na	151
III	Na	103
IV	Na	54
Unknow	Na	11
T		
Tis	1	1
T1	11	9
T2	45	68
T3	367	263
T4	119	44
Unknow	23	0
N		
N0	302	231
N1	134	88
N2	98	66
N3	6	0
Unknow	26	0
M		
M0	482	286
M1	61	54
Unknow	23	45
Fustat(n,%)		
Alive	371(12.97%)	306(79%)
Death	191(69.73%)	79(21%)
Unknow	4(17.30%)	0

### 3.2 Expression and mutation of 24 disulfidptosis-related genes

**Figure 1:**
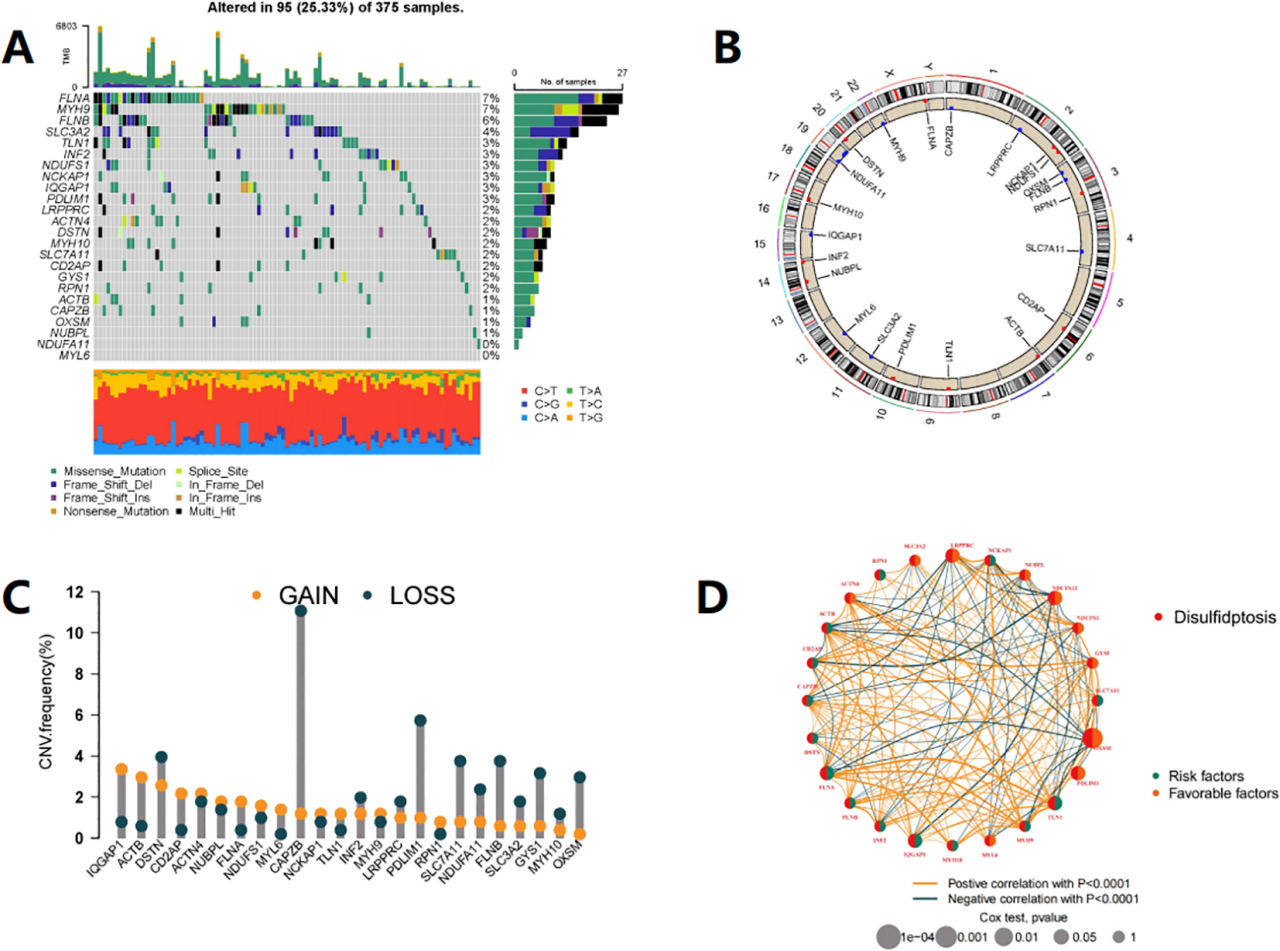
Expression and mutation profile of DRG in COAD. **(A)** Mutation frequency of 24 DRGs in the TCGA-COAD cohort of 375 patients. **(B)** The locations of CNV alterations in DRGs across 23 chromosomes, The red dots represent gain, while the blue dots represent loss. **(C)** The DRGs in TCGA-COAD chohrt show instances of gene copy number gain and gene copy number loss. **(D)** Interactions among DRGs in COAD. Each node represents a gene, the size of the node corresponds to the significance level (p-value), indicating the strength of the association between the gene and prognosis. The orange and green connecting lines represent positive and negative interactions between genes, respectively.

**Figure 1A** shows the mutation status of 24 DRGs in CRC patients, with a total mutation rate of 25.33%, with FLNA, MYH9, FLNB, and SLC3A2 having the highest mutation rates. In the copy number variation analysis of DRG (**Figure 1C**), we observed copy number gain of IQGAP1, ACTB, and DSTN, and copy number loss of CAPZB, PDLIM1, SLC7A11, FLNB, GYS1, and OXSM. **Figure 1B** shows the chromosomal location of DRG mutations. KM analysis divided the genes into high and low expression groups based on the median gene expression level, and 17 genes showed significant differences in survival between the two groups (P< 0.05) (**Supplementary Table S1**). **Figure 1D** illustrates the interaction relationships among 24 DRGs.

### 3.3 Identification of DRG subtypes in COAD

**Figure 2:**
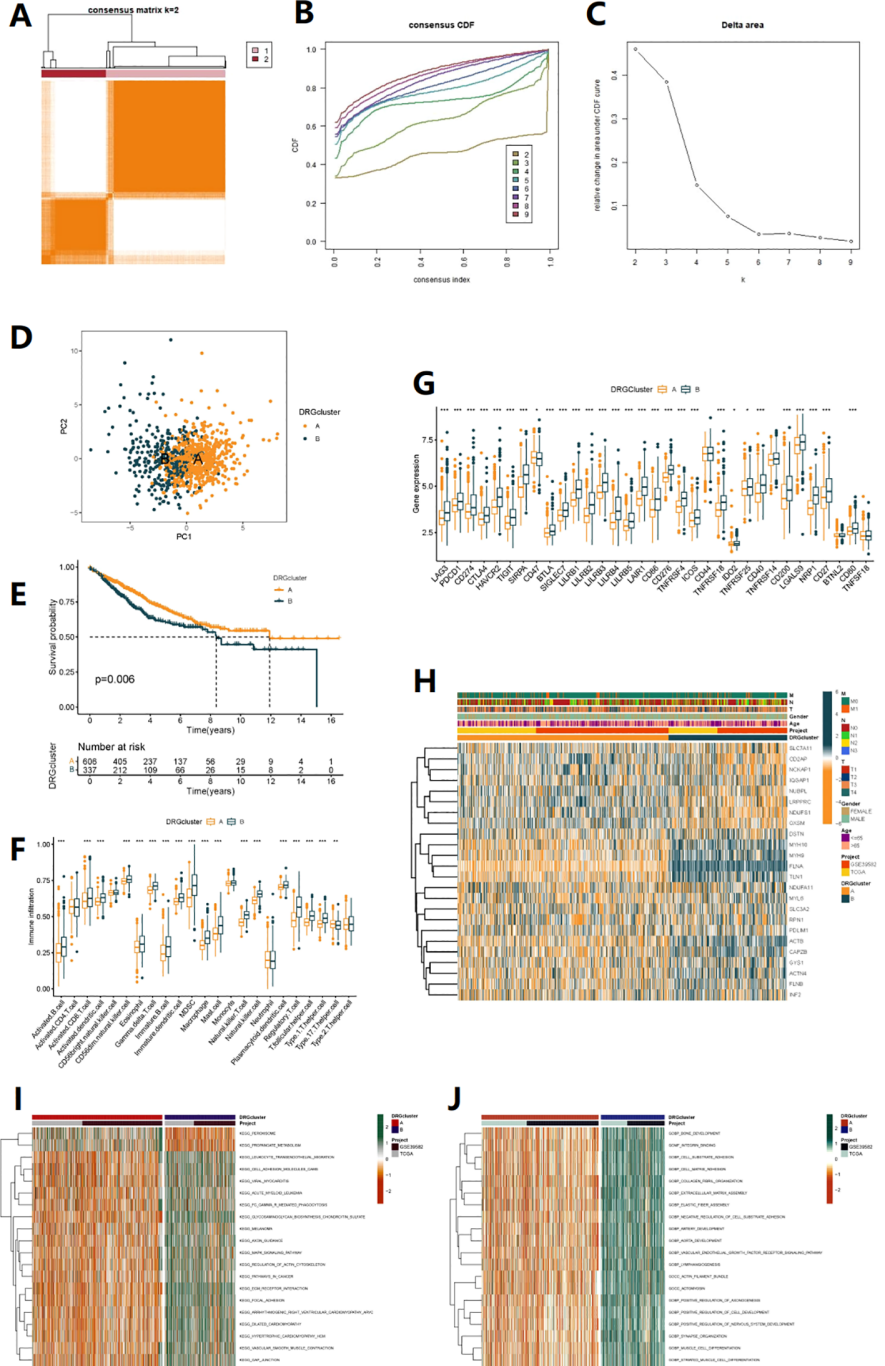
Clinicopathological and biological characteristics associated with two subtypes of DRG identified by consensus clustering analysis. **(A)** Unsupervised clustering of disulfidptosis-related genes and Consensus matrix heatmaps for k =2. **(B)** Cumulative Distribution Function (CDF) from k=2 to 9. **(C)** relative change in area under CDF curve. **(D)** PCA analysis. **(E)** Kaplan-Meier curve shows different overall survival (OS) between the two DRG subtypes. **(F)** Bundance of 23 infiltrating immune cells in the two DRG subtypes (*p< 0.05, **p< 0.01, ***p< 0.001).**(G)** Differential expression analysis of immune checkpoint genes between two subtypes. **(H)** Heatmap of clinical pathological features and expression of 24 DRGs in TCGA-COAD, GSE39582 cohorts. **(I, J)** GO and KEGG enrichment analysis between two subtypes, with orange indicating activation of related pathways and green indicating inhibition of related pathways.

To explore the relationship between the expression of 24 DRGs and Colorectal cancer, we used a clustering analysis to obtain the optimal K value (**Figure 2A**, **Supplementary Figure S1**). When k=2, patients were divided into two subtypes, A and B (**Figures 2A–D**, **Supplementary Table S2**). Survival analysis revealed that subtype A had a significant survival advantage (**Figure 2E**), and **Figure 2H** showed the clinical differences between subtypes A and B, with subtype B patients having higher tumor T and N staging. KEGG enrichment analysis showed that subtype A was mainly related to Propanoate Metabolism and Peroxisome. In contrast, subtype B was significantly enriched in MAPK Signaling Pathway, Pathways In Cancer and Fc Gammar R-mediated Phagocytosis (**Figure 2I**). GO annotation revealed that subtype B was mainly enriched in Cell Substrate Adhesion, as well as negative regulation of this process, with involvement in Vascular Endothelial Growth Factor Receptor Signaling Pathway, Artery Development, and Bone Development (**Figure 2J**).

Additionally, we used the CIBERSORT to analyze the immune cell infiltration in subtype B patients, which showed a significantly more abundant immune cell infiltration, including activated B cell, activated CD8 T cell, activated dendritic cell, CD56dim natural killer cell, eosinophil, gamma delta T cell, immature B cell, immature dendritic cell, myeloid-derived suppressor cell(MDSC), macrophage, mast cell, natural killer T cell, natural killer cell, regulatory T cell, T follicular helper cell, and others (**Figure 2F**). Most of the immune checkpoints, such as PD1, PD-L1, and CTLA-4, were also significantly higher expressed in subtype B (**Figure 2G**). Taken together, our results suggest that there are significant functional differences and immune microenvironment between the molecular subtypes of COAD based on DRG, which may be related to immune therapy response.

### 3.4 Identification of gene subtypes based on DEGs

**Figure 3:**
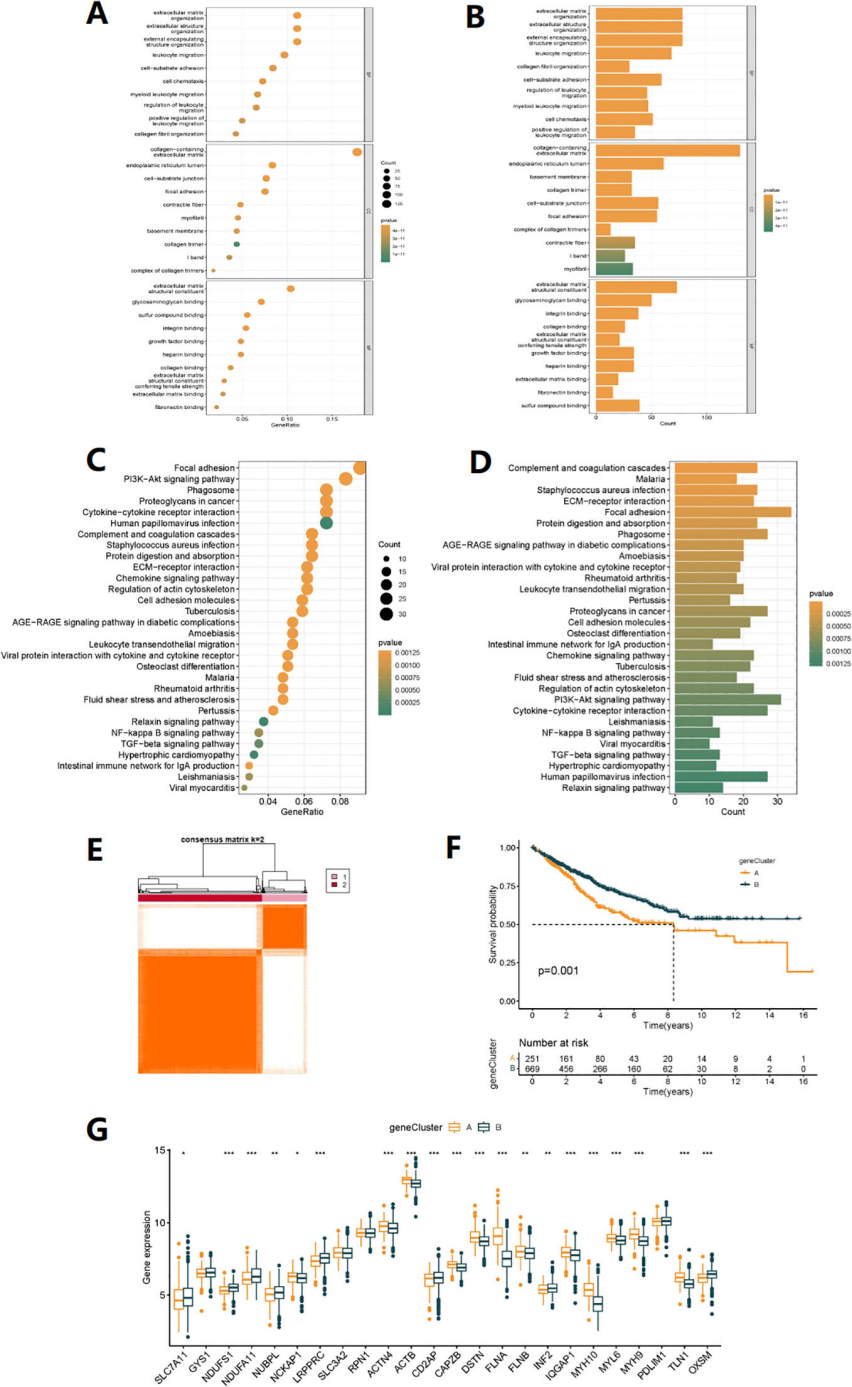
Gene subtype analysis based on DEGs. **(A, B)** GO enrichment analyses of DEGs among two DRG subtypes. **(C, D)** KEGG enrichment analyses of DEGs among two DRG subtypes. **(E)** The consensus clustering algorithm (k = 2) was used to divide all samples in TCGA-COAD and GSE35982 cohorts into two DRG gene subtypes. **(F)** Kaplan-Meier survival analysis of two gene subtypes. **(G)** Differences in the expression of 24 DRGs between two gene subtypes (*p< 0.05, **p< 0.01, ***p< 0.001).

We further explored the potential biological behaviors between the disulfidptosis-related subtypes and found 744 differentially expressed genes (DEGs) (**Supplementary Table S3**) between DRG subtypes A and B (**Supplementary Table S2**). In the molecular function (MF) results of GO annotation, it is worth noting that the DEGs were significantly enriched in sulfur compound binding pathways. In biological processes (BP), they were mainly related to extracellular structures, regulation of leukocyte migration, and positive regulation. In cellular components (CC), they were associated with intracellular structures, extracellular matrix composition, and cell-matrix connections (**Figures 3A, B**). KEGG enrichment analysis also suggested that DEGs were involved in the regulation of the Regulation of actin cytoskeleton (disulfidptosis can affect this regulation process), as well as pathways related to colon cancer development, including the PI3K-Akt signaling pathway, NF-kappa B signaling pathway, TGF-beta signaling pathway, and pathways related to cell signaling, immune response, cell adhesion, and apoptosis (**Figures 3C, D**). These results suggest that DEGs may be closely related to disulfidptosis and participate in colon cancer development through the above-mentioned pathways. Based on the expression of DEGs, clustering analysis classified patients into A and B gene subtypes (**Figure 3E**, **Supplementary Figure S2**, **Table S4**), with significant differences in prognosis between different subtypes of patients (**Figure 3F**). Finally, we analyzed the expression differences of 24 DRGs in different gene subtypes (**Figure 3G**).

### 3.5 Construction of disulfidptosis-related prognostic signature in ovarian cancer

**Figure 4:**
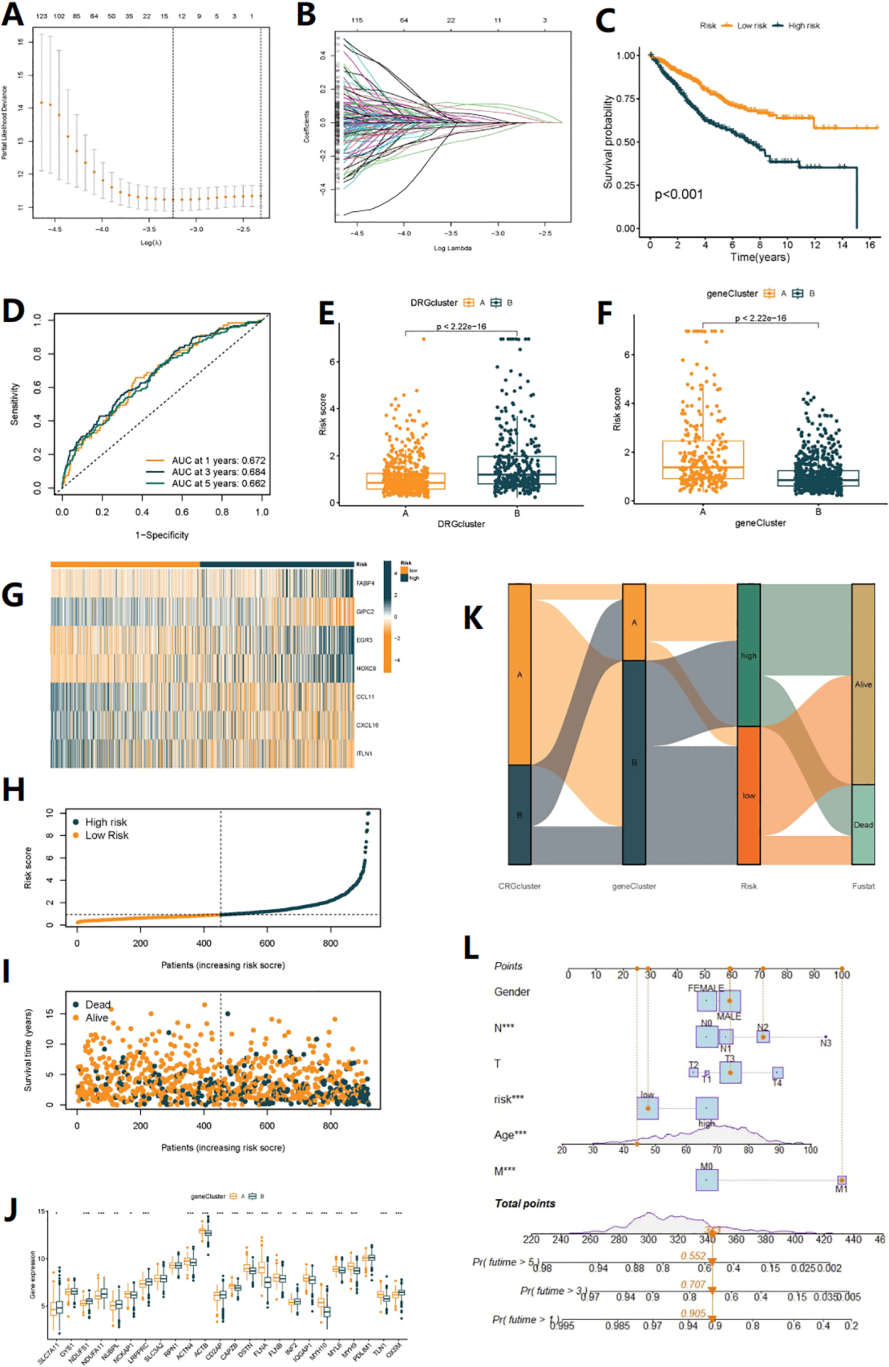
Construction of disulfidptosis-related prognostic signature. **(A, B)** The LASSO path plot shows the feature selection process. **(C)** Kaplan-Meier curve shows different overall survival (OS) between high and low-risk score groups. **(D)** ROC curves to predict the sensitivity and specificity of 1-, 3- and 5-year survival according to the Risk score. **(E, F)** Differences in Risk score between the two DRG clusters and the two gene clusters. **(G)** Expression of 7 DEGs in the high and low-risk groups. **(H, I)** Ranked dot and scatter plots showing the Risk score distribution and patient survival status, respectly. **(J)** Expression of 24 DRGs in the high and low-risk groups. **(K)** Alluvial diagram of subtype distributions in groups with different DRG_scores and survival outcomes (*p< 0.05, **p< 0.01, ***p< 0.001). **(L)** Nomogram can integrate patients’ clinical features and risk scores to predict patient prognosis.

Seven genes were identified for constructing the risk model through Lasso regression analysis (**Figures 4A, B**). Risk score =(0.200*expression of FABA4)+(-0.182*expression of GIPC2)+(0.249*expression of EGR3)+(0.133*expression of HOXC6)+(-0.120*expression of CCL11)+(-0.192*expression of CXCL10)+(-0.056*expression of ITLN1). Prognostic and risk analyses were performed for the risk models constructed for the total cohort (n=920), training set (n=460), and validation set (n=460). The results of the three datasets were consistent, with better prognosis for low-risk patients (**Figure 4C**, **Supplementary Figures S3A**, **Figure S4A**). The model had a certain value in predicting the prognosis of patients at 1, 3, and 5 years (**Figure 4D**, **Supplementary Figure S3B**, **Figure S4B**). As the risk increased, the number of deaths from COAD also increased (**Figures 4G-I**, **Supplementary Figures S3B-E**, **Figures S4B-E**). By combining the clinical feature score and risk score, a column chart could predict the survival time of patients. For example, a patient with a risk score of 363 had a 90.5% probability of surviving for 1 year, a 67.6% probability of surviving for 3 years, and a 58.5% probability of surviving for 5 years (**Figure 4L**). Additionally, we found that DRG subtypes, gene subtypes, and high/low-risk groups were closely related (**Figures 4E, F, K**), with better prognosis for DRG subtype A, gene subtype B, and low-risk group, while worse prognosis for DRG subtype B, gene subtype A, and high-risk group, with consistent results. **Figure 4J** showed the expression differences of 24 DRGs between high and low-risk groups.

### 3.6 SHAP(SHapley Additive exPlanations)analysis

**Figure 5:**
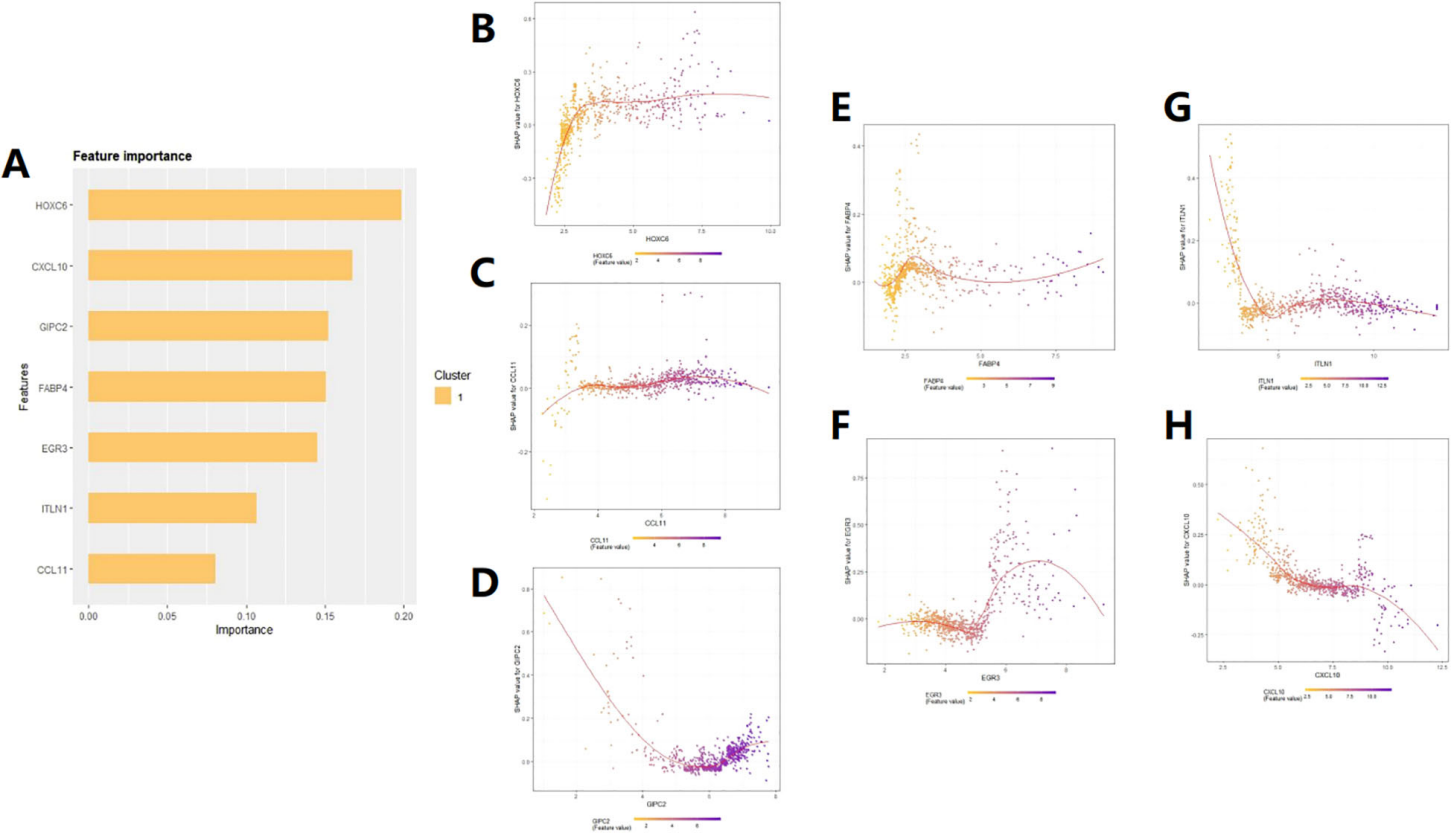
SHAP feature importance analysis. **(A)** Ranking of the impact of the 7 feature genes on patient prognosis. **(B–H)** Prediction of patient prognosis based on the relationship between SHAP values and the expression levels of the seven target genes.

SHAP analysis can rank the impact of these 7 target genes on prognosis (**Figure 5A**) and quantify their positive or negative impact on prognosis based on gene expression levels (**Figures 5B-H**). The upward trend and downward trend on the curve respectively indicate a positive and negative impact on patient prognosis. For example, the expression level of HOXC6 tends to stabilize with a certain degree of expression, indicating a stable impact on patient prognosis (**Figure 5B**). CCL11 and GIPC2 are primarily expressed on the upward curve (**Figures 5C, D**), indicating a positive impact on patient prognosis. When the expression of FABP4 is greater than 2.5, it begins to have a negative impact on patient prognosis (**Figure 5E**). EGR3 is mainly expressed on the downward trend curve (**Figure 5F**). TLIN1 and CXCL10 also have different effects on patient prognosis at different expression ranges (**Figures 5G, H**). By combining the COX risk coefficients, we can intuitively judge the specific impact of these genes on the prognosis of COAD patients based on the changes in their expression range.

### 3.7 TMB analysis and survival analysis of TMB

**Figure 6:**
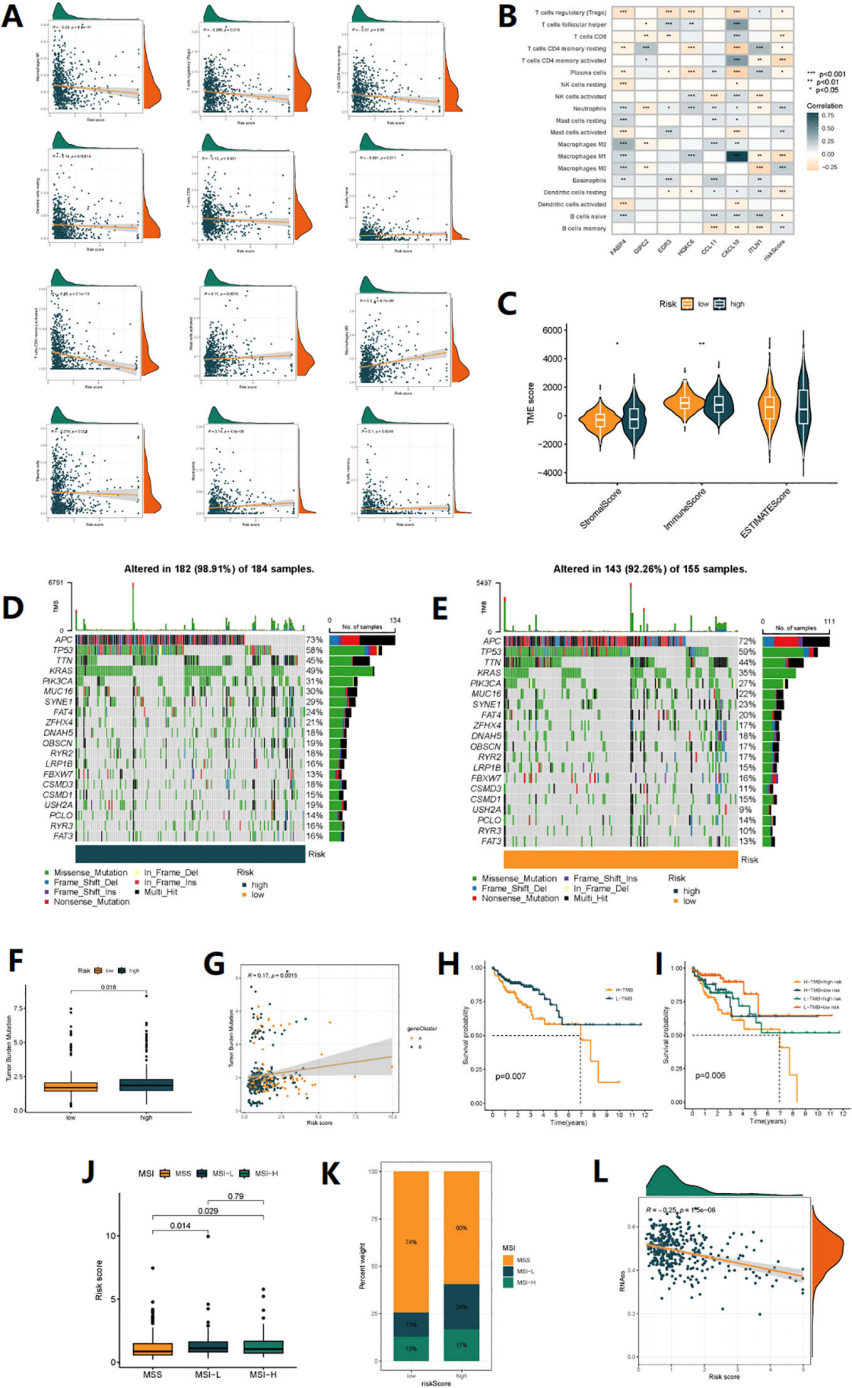
TMB analysis and immune microenvironment analysis. **(A)** Correlations between Risk score and immune cell types. **(B)** The correlation between immune cell abundance and three genes in the risk model. **(C)** Comparison of StromalScores, ImmuneScores, and ESTIMATE Scores between high-risk and low-risk patients. **(D, E)** mutation status of all genes in high-risk and low-risk groups of patients is displayed separately. **(F)** Comparison of TMB levels between high-risk and low-risk patients. **(G)** The linear variation of tumor mutational burden (TMB) influenced by risk scores. **(H)** The KM curve graph indicates the impact of high and low TMB on patient survival. **(I)** The KM analysis assessed the differences in survival among patients with different TMB levels and risk scores. **(J)** The relationship between different MSI statuses and risk scores. **(K)** The proportion of different MSI statuses in the high-risk and low-risk groups. **(L)** The relationship between Stemness Scores and risk score.

Using the CIBERSORT analysis, we observed that naive B cells, resting dendritic cells, M1 macrophages, plasma cells, memory activated CD4+ T cells, memory resting CD4+ T cells, CD8+ T cells, and regulatory T cells (Tregs) were negatively associated with the Risk score. In contrast, memory B cells, M0 macrophages, activated mast cells, and neutrophils were positively associated with the risk score (**Figure 6A**). **Figure 6B** shows the close relationship between the 7 DRGs and the levels of immune cell infiltration. In addition, the high-risk group had a higher immuneScore and a lower stromalScore (*p*<0.05) (**Figure 6C**). The overall mutation rate and TMB were higher in the high-risk group compared to the low-risk group (**Figures 6D-G**), and patients with high TMB had poorer prognosis than those with low TMB (**Figure 6H**). Combining patient survival data, we further analyzed and found that patients with high risk and high TMB levels had the worst prognosis, followed by patients with low TMB levels in the high-risk group, consistent with the previous findings. However, in the low-risk group, there was no statistically significant impact of TMB levels on the prognosis of these patients (**Figure 6I**). Correspondingly, high-risk score was associated with MSI-H and MSI-L status, while low-risk score was associated with MSS status (P<0.05) (**Figure 6J**). **Figure 6K** also showed that the proportion of MSI-H and MSI-L was higher in the high-risk group, indicating that patients in the high-risk group may be more sensitive to immunotherapy. Finally, stem cell-related analysis showed a negative correlation between patient risk score and stem cell index, which means that colon cancer cells with lower risk scores have more obvious stem cell characteristics and lower cell differentiation levels (**Figure 6L**).

### 3.8 Drug susceptibility analysis

**Figure 7:**
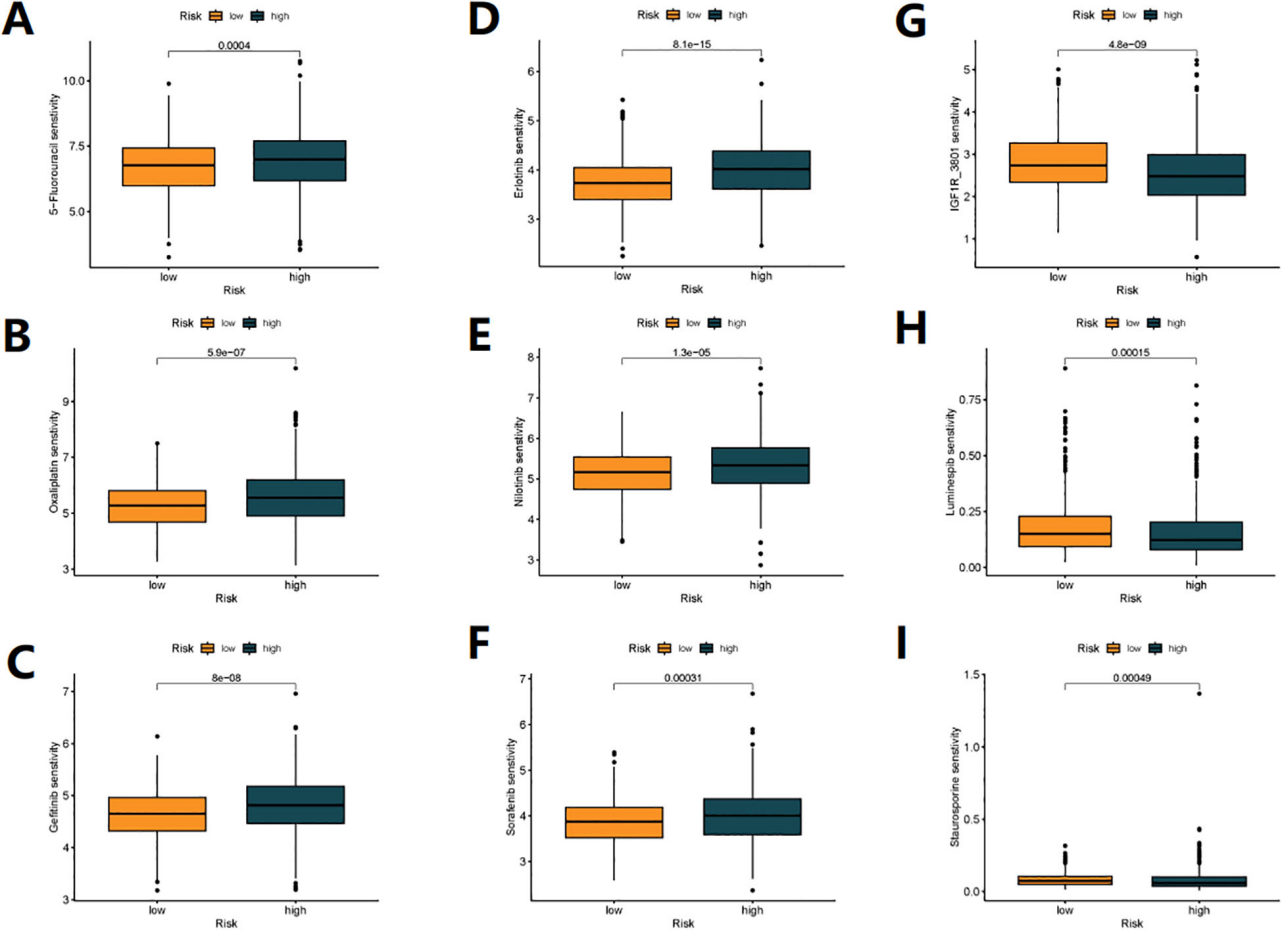
**(A–I)** Relationships between DRG_score and Drug susceptibility.

We analyzed the sensitivity of 198 drugs using the “oncoPredict” package and found that 66 drugs had differences in sensitivity between high-risk and low-risk patients (**Supplementary Figure S5**). Of note, first-line chemotherapy drugs for colon cancer, 5-Fluorouracil (**Figure 7A**) and Oxaliplatin (**Figure 7B**), as well as targeted molecular drugs Gefitinib (**Figure 7C**), Erlotinib (**Figure 7D**), Nilotinib (**Figure 7E**), and Sorafenib (**Figure 7F**) were more effective in low-risk patients. On the other hand, IGF1R_3801 (**Figure 7G**), Luminespib (**Figure 7H**) and Staurosporine (**Figure 7I**) were more effective in high-risk patients. These results may help us in future risk stratification and individual treatment selection for patients.

### 3.9 Single-cell level research

**Figure 8:**
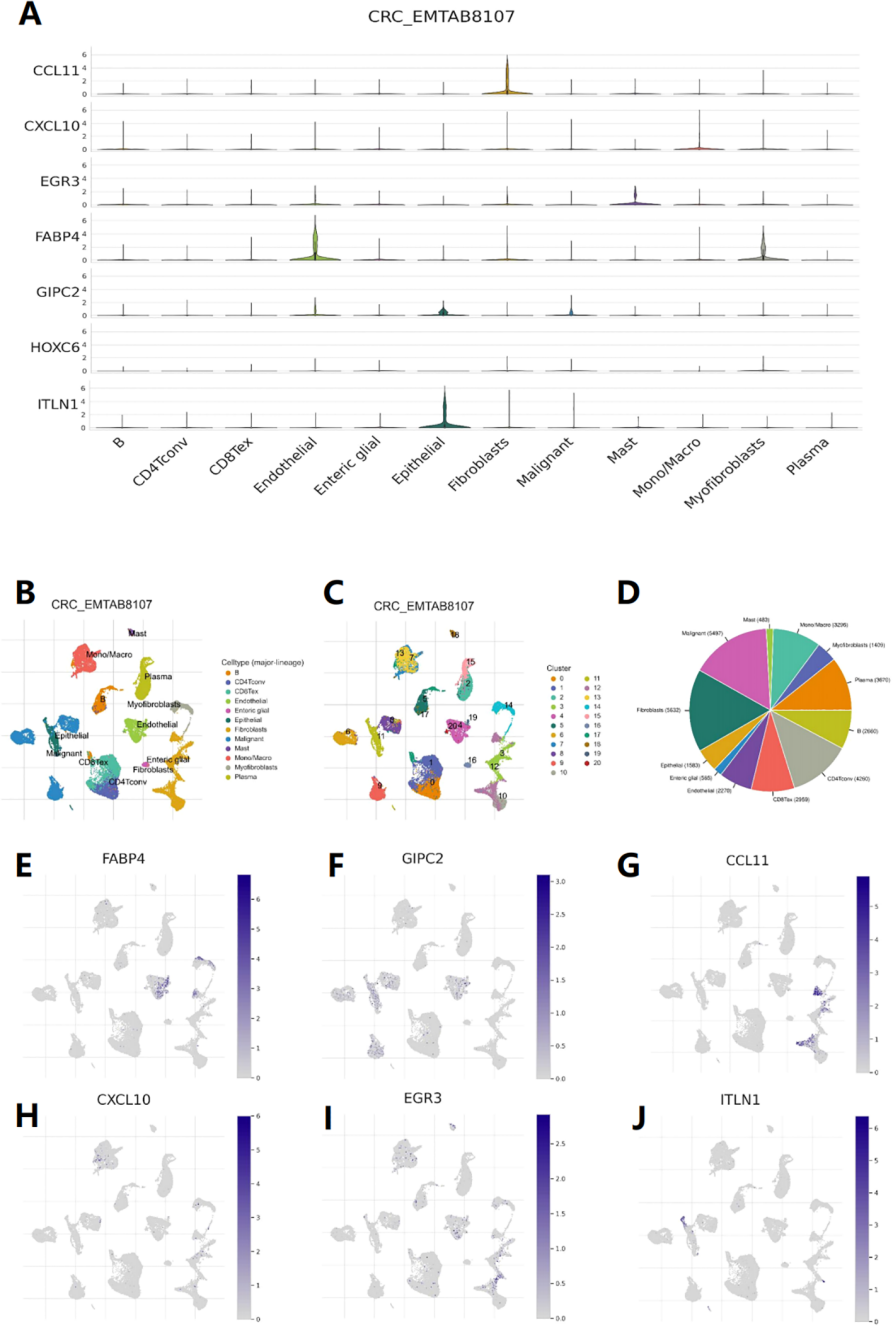
Single-cell level analysis. **(A)** Violin diagram shows the distribution of 7 feature genes expression in different cells. **(B)** Single-cell type map of major-lineage. **(C)** Single-cell cluster map. **(D)** Sunburst plot for single-cell classification. **(E-J)** The cell type map shows the expression of 7 feature genes at different single-cell levels.

Through the analysis of dataset EMTAB8107, it was found that CCL11 is mainly expressed in fibroblasts (**Figure 8G**), CXCL10 is expressed at the highest level in macrophages (**Figure 8H**), EGR3 is expressed in both macrophages and mast cells (**Figure 8I**), ITLN1, GIPC2, and FABP4 are mainly expressed in epithelial cells (endothelial and epithelial), and FABP4 is also expressed in myofibroblasts (**Figures 8E, F, J**). These results suggest that in addition to immune cells, these genes may also play a role in stromal cells outside of cancer cells. The expression levels of 7 genes across different cell types were obtained (**Figure 8A**), along with Uniform Manifold Approximation and Projection (UMAP) plots (**Figures 8B, C**), and a pie chart (**Figure 8D**).

### 3.10 Real-time quantitative reverse transcription PCR

**Figure 9:**
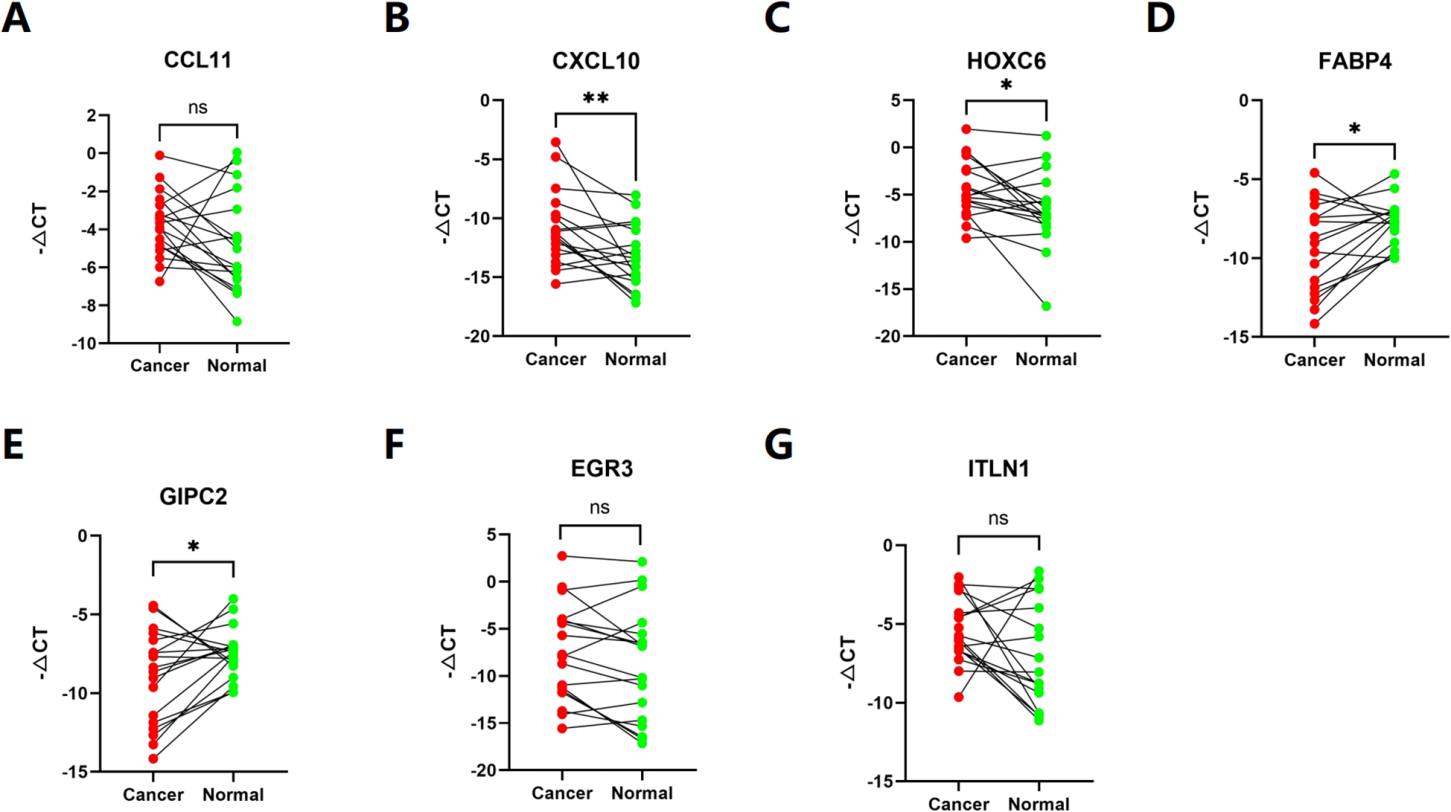
**(A–G)** The expression of each gene in 15 pairs of clinical COAD tissues and adjacent normal tissues. (ns p>0,05, *p< 0.05, **p< 0.01).

To determine whether 7 disulfidptosis prognostic genes are differentially expressed in colorectal cancer tissues, we used qRT-PCR to analyze the expression of each gene in 15 pairs of clinical COAD tissues and adjacent normal tissues. The results showed that the expression level in COAD tissues was different from that in normal tissues (**Figures 9A-G**).

## 4 Discussion

Colorectal cancer exhibits high heterogeneity, with different populations having different treatment sensitivity and clinical prognosis. Although many molecular biomarkers have emerged for predicting patient survival and treatment response, the existing biomarkers still have poor clinical validation. New key markers and treatment strategies are continuously being explored. Meanwhile, disulfidptosis has become a hot topic in recent years. Current research finds that under conditions of oxidative stress, certain cysteine residues in proteins of tumor cells can form disulfide bonds. If this crosslinking is too severe, it can lead to abnormal folding and assembly of proteins, resulting in the loss of normal biological function. The redox state is one of the important mechanisms for the occurrence and development of tumors (21), such as some tumor cells increase their survival and tolerance by changing the intracellular redox protein (22). Studies have shown that disulfidptosis may have potential applications in tumor therapy. For example, some anticancer drugs, such as cisplatin and paclitaxel, exert their anticancer effects by reacting with intracellular disulfides (23).

To explore strategies for guiding personalized treatment for COAD patients, our study comprehensively analyzed the expression and mutation status of 24 DRG in COAD and identified two distinct subtypes. Patients with subtype B had poorer prognosis and were significantly enriched in cancer-related pathways in KEGG analysis and a marked enrichment in immune cell phagocytosis and extracellular matrix adhesion in GO annotation. CIBERSORT analysis suggested significant differences in the tumor microenvironment between subtypes, with subtype B having a higher composition of immune cells, including macrophages. Macrophages are a component of the MPS and play a crucial role in maintaining innate immune responses, tissue homeostasis, and inflammation (24). Tumor-associated macrophages (TAMs) can be polarized from macrophages and are the most abundant immune cells in CRC. TAMs can interact with tumor cells through the secretion of exosomes or various cytokines, promoting tumor cell proliferation, migration, and angiogenesis. TAMs also recruit regulatory T cells (Tregs) through the CCL2, which inhibits T cell immune (25)response function, leading to an immunosuppressive microenvironment in CRC (24, 25). Additionally, subtype B had a higher abundance of MDSCs, which may inhibit the activity of immune cells, leading to immune escape and tumor progression (26).

Further analysis of DEGs between subtypes revealed significant enrichment in sulfur compound binding and regulation of the actin cytoskeleton. Current research suggests that during the process of apoptosis, actin undergoes a transition from a polymerized state to a depolymerized state, affecting the cytoskeletal structure within cells, leading to changes in cell morphology and restricted cell movement. In addition, changes in actin polymerization can also affect intracellular signal transduction, thus affecting cell metabolism and survival, ultimately leading to cell death (5).

In the end, we conducted a screen of the differentially expressed genes (DEGs) and, through LASSO regression, identified seven key genes associated with prognosis. These genes were used to construct a risk prognosis model related to disulfidptosis, which can stratify COAD patients into high-risk and low-risk groups. The nomogram can predict individualized clinical outcomes. During the model construction, we obtained risk coefficients for each gene. A positive risk coefficient indicates that the higher the expression level of this gene, the higher the patient’s risk score. Conversely, a negative risk coefficient suggests that the higher the expression level of the gene, the lower the patient’s risk score. Additionally, we further employed SHAP feature importance analysis to address the black-box issue inherent in machine learning. We not only ranked the genes based on their impact on prognosis but also validated and explained how specific expression levels of each gene contribute to patient prognosis.

FABP4 is a fatty acid-binding protein that plays a complex role in the development, progression, and prognosis of COAD. Previous studies have shown that the expression of FABP4 is significantly associated with advanced tumor staging, poorer disease-free survival, and overall survival in colorectal cancer, consistent with our findings (27). Further research has explored the underlying mechanisms and discovered that the expression of FABP4 increases ROS (reactive oxygen species) levels, leading to the activation of the ERK (extracellular signal-regulated kinase) pathway, which in turn activates mTOR (mammalian target of rapamycin), thereby promoting tumor cell growth.GIPC2 is an important member of the PDZ domain family, and previous studies have suggested that GIPC2 may play a critical role in tumor development and embryonic development by promoting interactions between G protein heterotrimers and Wnt receptors or receptor tyrosine kinases (28). Whole-genome sequencing revealed the presence of missense mutations F74Y and R312Q, as well as a nonsense mutation E216X, in GIPC2 in colorectal cancer (29). The E216X nonsense mutation is a deleterious mutation that results in the loss of the GH2 domain, which prevents GIPC2 from binding to MY06 (30). Arnon et al. reported that EGR3 is a member of the Early Growth Response gene family and participates in many biological processes such as cell proliferation, differentiation, and apoptosis. Studies have shown that this gene is associated with cancer cell migration, making it highly correlated with tumor progression, and its expression levels have been used as prognostic markers for various cancers, including CRC (31). In colon cancer cell lines, EGR3 has binding sites in several genes associated with resistance to the cancer treatment drug 5-fluorouracil. EGR3 can regulate the expression of these genes to affect the sensitivity of cells to 5-fluorouracil, thereby affecting the efficacy of cancer treatment (32). HOXC6 is a gene that encodes a transcription factor. Studies have reported that high expression of HOXC6 in CRC can promote tumor metastasis by activating the classical WNT pathway and promoting proliferation through the TGF-β/smad pathway (33). ITLN1 is an inflammatory factor that may be associated with various tumor diseases, including pleural mesothelioma, gastric cancer, and prostate cancer. Katsuya et al. found that its expression is significantly increased in the tumor tissues of colorectal cancer (CRC) patients, and compared with CRC tissues, the expression of ITLN1 also shows a gradient decrease in adenomatous polyps/serrated polyps and normal tissues (34). Conversely, the research results of Zhang Y et al. suggest that ITLN1 is positively correlated with a good prognosis in CRC patients (35). A part of our research results predicts that ITLN1 may have different degrees of impact on the prognosis of COAD patients at different expression levels. Considering that the previous research has a relatively single population of samples, it may be necessary to expand the sample size when necessary. The impact of ITLN1 on the prognosis of colorectal cancer and its underlying mechanisms are worthy of further exploration in the future. CCL11, also known as Eotaxin-1, is a chemokine that primarily attracts cells such as eosinophils and basophils to inflammatory sites, participating in biological processes such as immune regulation and inflammation. Studies have shown that Eotaxin-1 and its receptors are significantly upregulated in colorectal cancer, especially CCL11 and CCR3 (the receptor for CCL11) (36). Tripathi et al. (37) demonstrated on breast cancer cells that TAMs undergo phenotypic changes and aggregate in the hypoxic regions of tumors. Hypoxic tumor cells exhibit upregulation of intracellular eotaxin levels, which together promote tumor progression. CXCL10 is also a chemokine that attracts and activates immune cells, participating in tumor development and prognosis. Shang et al (38) found that CXCL10 can attract CD8+ T cells to infiltrate into tumor tissues and exert a cytotoxic effect. In addition, CXCL10 can also promote vascular normalization and increase the sensitivity of colorectal cancer to cetuximab combined with PD-1 checkpoint inhibitors (39).

TMB and MSI are important biomarkers for predicting potential response to immune therapy. The higher the TMB, the greater the number and type of neoantigens produced by tumor cells, making it easier to activate specific anti-tumor immune responses, which may make immune therapy more effective (40). MSI is caused by defects in the DNA mismatch repair (MMR) system, resulting in instability of DNA microsatellite sequences. Patients with high MSI colon cancer have a better response to PD-1/PD-L1 inhibitors.

Our study found that patients with high-risk scores had a higher overall mutation rate, higher TMB, and a higher proportion of MSI-H status. In addition, TMB significantly affected patient prognosis, and the combination of TMB and DRG risk scores may be an effective prognostic biomarker for colon cancer patients. Finally, we validated the sensitivity of first-line chemotherapy drugs Oxaliplatin, 5-Fluorouracil, and targeted drugs. The results showed that the risk model has the potential to stratify colon cancer patients for individualized risk assessment and may serve as a biomarker for identifying different patients’ sensitivity to immune therapy, chemotherapy drugs, and targeted drugs in the future.

In summary, we conducted cluster analysis on DRG and screened out differentially expressed genes for DRG subtypes. Subsequently, we constructed a prognostic risk model using 7 biomarkers to validate its predictive value for COAD patient prognosis. Although our model has outstanding ability in identifying patients’ immune status and predicting their prognosis, there are still limitations that need to be addressed in subsequent studies. Data analysis based on public databases may lead to deviations between predictions and actual situations. More COAD patient data are needed in the future to verify the usefulness of this model and the accuracy of treatment predictions. In addition, more prospective and fundamental research is needed to complete the details of this study.

## Data Availability

The datasets presented in this study can be found in online repositories. The names of the repository/repositories and accession number(s) can be found in the article/**Supplementary Material**.

## Supplementary material

Supplementary Figure S1 Unsupervised clustering of disulfidptosis-related genes and Consensus matrix heatmaps for k = 3-9.

Supplementary Figure S2 Unsupervised clustering of DEGs and Consensus matrix heatmaps for k = 3-9.

Supplementary Figure S3 Construction of disulfidptosis-related prognostic signature based on the train sets. **(A)** Kaplan-Meier curve shows different overall survival (OS) between high and low-risk score groups in train sets. **(B)** ROC curves to predict the sensitivity and specificity of 1-, 3- and 5-year survival according to the Risk score. **(C)** Expression of 7 DEGs in the high and low-risk groups. **(D, E)** Ranked dot and scatter plots showing the Risk score distribution and patient survival status, respectly.

Supplementary Figure S4 Construction of disulfidptosis-related prognostic signature based on the test sets. **(A)** Kaplan-Meier curve shows different overall survival (OS) between high and low-risk score groups in test sets. **(B)** ROC curves to predict the sensitivity and specificity of 1-, 3- and 5-year survival according to the Risk score. **(C)** Expression of 7 DEGs in the high and low-risk groups. **(D, E)** Ranked dot and scatter plots showing the Risk score distribution and patient survival status, respectly.

## References

[1] Bray F Ferlay J Soerjomataram I Siegel R. Global cancer statistics 2018: GLOBOCAN estimates of incidence and mortality worldwide for 36 cancers in 185 countries. CA Cancer J Clin. (2018) 68:394–424. doi: 10.3322/caac.21492 30207593

[2] Munro MJ Wickremesekera SK Peng L Tan ST Itinteang T. Cancer stem cells in colorectal cancer: A review. J Clin Pathol. (2018) 71:110–6. doi: 10.1136/jclinpath-2017-204739 28942428

[3] Xi Y Xu P. Global colorectal cancer burden in 2020 and projections to 2040. Transl Oncol. (2021) 14:101174. doi: 10.1016/j.tranon.2021.101174 34243011 PMC8273208

[4] Miller KD Nogueira L Mariotto AB Rowland JH Yabroff KR Alfano CM. Cancer treatment and survivorship statistics. CA Cancer J Clin. (2019) 69:363–85. doi: 10.3322/caac.21565 31184787

[5] Liu X Nie L Zhang Y Yan Y Wang C. Actin cytoskeleton vulnerability to disulfide stress mediates disulfidptosis. Nat Cell Biol. (2023) 25:404–14. doi: 10.1038/s41556-023-01091-2 PMC10027392 36747082

[6] Hogg PJ. Biological regulation through protein disulfide bond cleavage. Redox report: Commun Free Radical Res. (2002) 7:71–7. doi: 10.1179/135100002125000299 12189052

[7] Su Z Yang Z Xu Y Chen Y Yu Q. Apoptosis, autophagy, necroptosis, and cancer metastasis. Mol Cancer. (2015) 14:48. doi: 10.1186/s12943-015-0321-5 25743109 PMC4343053

[8] Ritchie ME Phipson B Wu D Hu Y Law CW Shi W. limma powers differential expression analyses for RNA-sequencing and microarray studies. Nucleic Acids Res. (2015) 20:e47. doi: 10.1093/nar/gkv007 PMC4402510 25605792

[9] Chen H Yang W Li Y Ma L Ji Z. Leveraging a disulfidptosis-based signature to improve the survival and drug sensitivity of bladder cancer patients. Front Immunol. (2023) 14:1198878. doi: 10.3389/fimmu.2023.1198878 37325625 PMC10266281

[10] Barretina J Caponigro G Stransky N Venkatesan K. The Cancer Cell Line Encyclopedia enables predictive modelling of anticancer drug sensitivity. Nature. (2012) 483:603–7. doi: 10.1038/nature11003 PMC3320027 22460905

[11] Wilkerson MD Hayes DN. ConsensusClusterPlus: a class discovery tool with confidence assessments and item tracking. Bioinf (Oxford England). (2010) 26:1572–3. doi: 10.1093/bioinformatics/btq170 PMC2881355 20427518

[12] Liberzon A. The Molecular Signatures Database (MSigDB) hallmark gene set collection. Cell Syst. (2015) 1:417–25. doi: 10.1016/j.cels.2015.12.004 PMC4707969 26771021

[13] Subramanian A. Gene set enrichment analysis: a knowledge-based approach for interpreting genome-wide expression profiles. Proc Natl Acad Sci U.S.A. (2005) 102:15545–50. doi: 10.1073/pnas.0506580102 PMC1239896 16199517

[14] Ashburner M. Gene ontology: tool for the unification of biology. The Gene Ontology Consortium. Nat Genet. (2000) 25:25–9. doi: 10.1038/75556 PMC3037419 10802651

[15] Kanehisa M Goto S. KEGG: kyoto encyclopedia of genes and genomes. Nucleic Acids Res. (2000) 28:27–30. doi: 10.1093/nar/28.1.27 10592173 PMC102409

[16] Yu G Wang L Han Y He Q. clusterProfiler: an R package for comparing biological themes among gene clusters. Omics: J Integr Biol. (2012) 16:284–7. doi: 10.1089/omi.2011.0118 PMC3339379 22455463

[17] Lundberg SM Erion G Chen H DeGrave A Prutkin JM Nair B. From local explanations to global understanding with explainable AI for trees. Nat Mach Intell. (2020) 2:56–67. doi: 10.1038/s42256-019-0138-9 32607472 PMC7326367

[18] Becht E Giraldo NA Lacroix L Buttard B Elarouci N Petitprez F. Estimating the population abundance of tissue-infiltrating immune and stromal cell populations using gene expression. Genome Biol. (2016) 17:218. doi: 10.1186/s13059-016-1070-5 27765066 PMC5073889

[19] Chalmers ZR. Analysis of 100,000 human cancer genomes reveals the landscape of tumor mutational burden. Genome Med. (2017) 9:34. doi: 10.1186/s13073-017-0424-2 28420421 PMC5395719

[20] Lawrence MS. Mutational heterogeneity in cancer and the search for new cancer-associated genes. Nature. (2013) 499:214–8. doi: 10.1038/nature12213 PMC3919509 23770567

[21] Iyamu EW. The redox state of the glutathione/glutathione disulfide couple mediates intracellular arginase activation in HCT-116 colon cancer cells. Digestive Dis Sci. (2010) 55:2520–8. doi: 10.1007/s10620-009-1064-1 19997976

[22] Sideris S. Efficacy of weekly paclitaxel treatment as a single agent chemotherapy following first-line cisplatin treatment in urothelial bladder cancer. Mol Clin Oncol. (2016) 4:1063–7. doi: 10.3892/mco.2016.821 PMC4887921 27284445

[23] Mitin T Hunt D Shipley WU Kaufman DS Uzzo R Wu CL. Transurethral surgery and twice-daily radiation plus paclitaxel-cisplatin or fluorouracil-cisplatin with selective bladder preservation and adjuvant chemotherapy for patients with muscle invasive bladder cancer (RTOG 0233): a randomised multicentre phase 2 trial. Lancet Oncol. (2013) 14:863–72. doi: 10.1016/S1470-2045(13)70255-9 PMC3955198 23823157

[24] Liu Y Cao X. The origin and function of tumor-associated macrophages. Cell Mol Immunol. (2015) 12:1–4. doi: 10.1038/cmi.2014.83 25220733 PMC4654376

[25] Yang L Zhang Y. Tumor-associated macrophages: From basic research to clinical application. J Hematol Oncol. (2017) 10:58. doi: 10.1186/s13045-017-0430-2 28241846 PMC5329931

[26] Ouyang LY Wu XJ Ye SB Zhang RX Li ZL. Tumor-induced myeloid-derived suppressor cells promote tumor progression through oxidative metabolism in human colorectal cancer. J Transl Med. (2015) 13:47. doi: 10.1186/s12967-015-0410-7 25638150 PMC4357065

[27] Kim SH Pyo JS Son BK Oh IH Min KW. Clinicopathological significance and prognostic implication of nuclear fatty acid-binding protein 4 expression in colorectal cancer. Pathol Res Pract. (2023) 249:154722. doi: 10.1016/j.prp.2023.154722 37591068

[28] Liu Y Lou W Chen G Ding B Kuang J. Genome-wide screening for the G-protein-coupled receptor (GPCR) pathway-related therapeutic gene RGS19 (regulator of G protein signaling 19) in bladder cancer. Bioengineered. (2021) 12:5892–903. doi: 10.1080/21655979.2021.1971035 PMC8806424 34482807

[29] Berger MF Hodis E Heffernan TP Deribe YL Lawrence MS. Melanoma genome sequencing reveals frequent PREX2 mutations. Nature. (2012) 485:502–6. doi: 10.1038/nature11071 PMC3367798 22622578

[30] Network, C.G.A. Comprehensive molecular characterization of human colon and rectal cancer. Nature. (2012) 487:330–7. doi: 10.1038/nature11252 PMC3401966 22810696

[31] Knudsen AM Eilertsen I Kielland S Pedersen MW Sørensen MD Dahlrot RH. Expression and prognostic value of the transcription factors EGR1 and EGR3 in gliomas. Sci Rep. (2020) 10:9285. doi: 10.1038/s41598-020-66236-x 32518380 PMC7283475

[32] Szoke D Gyorffy A Surowiak P Tulassay Z Dietel M Gyorffy B. Identification of consensus genes and key regulatory elements in 5-fluorouracil resistance in gastric and colon cancer. Onkologie. (2007) 30:421–6. doi: 10.1159/000104490 17848813

[33] Ji ML Feng Q He G Yang L Tang W Lao X. Silencing homeobox C6 inhibits colorectal cancer cell proliferation. Oncotarget. (2016) 7:29216–27. doi: 10.18632/oncotarget.v7i20 PMC5045391 27081081

[34] Katsuya N Sentani K Sekino Y Yamamoto Y Kobayashi G Babasaki T. Clinicopathological significance of intelectin-1 in colorectal cancer: Intelectin-1 participates in tumor suppression and favorable progress. Pathol Int. (2020) 70:943–52. doi: 10.1111/pin.v70.12 33002285

[35] Zhang Y Gao T Wu M Xu Z Hu H. Value analysis of ITLN1 in the diagnostic and prognostic assessment of colorectal cancer. Trans Cancer Res. (2024) 13:2877–91. doi: 10.21037/tcr-24-137 PMC11231763 38988920

[36] Komura T Yano M Miyake A Takabatake H Miyazawa M Ogawa N. Immune condition of colorectal cancer patients featured by serum chemokines and gene expressions of CD4+ Cells in blood. Can J Gastroenterol Hepatol. (2018) 2018:7436205. doi: 10.1155/2018/7436205 PMC6016223 29992127

[37] Tripathi C Tewari BN Kanchan RK Baghel KS Nautiyal N Shrivastava R. Macrophages are recruited to hypoxic tumor areas and acquire a pro-angiogenic M2-polarized phenotype via hypoxic cancer cell derived cytokines Oncostatin M and Eotaxin. Oncotarget. (2014) 5:5350–68. doi: 10.18632/oncotarget.v5i14 PMC4170629 25051364

[38] Shang S Yang YW Chen F Yu L Shen SH Li K. TRIB3 reduces CD8+ T cell infiltration and induces immune evasion by repressing the STAT1-CXCL10 axis in colorectal cancer. Sci Transl Med. (2022) 14:eabf0992. doi: 10.1126/scitranslmed.abf0992 34985967

[39] Yan W Qiu L Yang M Xu A Ma M Yuan Q. CXCL10 mediates CD8+ T cells to facilitate vessel normalization and improve the efficacy of cetuximab combined with PD-1 checkpoint inhibitors in colorectal cancer. Cancer Lett. (2023) 567:216263. doi: 10.1016/j.canlet.2023.216263 37354983

[40] Klempner SJ Fabrizio D Bane S Reinhart M Peoples T Ali SM. Tumor mutational burden as a predictive biomarker for response to immune checkpoint inhibitors: A review of current evidence. Oncologist. (2020) 25:e147–159. doi: 10.1634/theoncologist.2019-0244 PMC6964127 31578273

