# Supplementary figures and images for: Role of disulfidptosis in colorectal adenocarcinoma: implications for prognosis and immunity
Source: Front Immunol. 2024 Sep 27;15:1409149. doi: 10.3389/fimmu.2024.1409149 (PMC11466812; doi:10.3389/fimmu.2024.1409149)

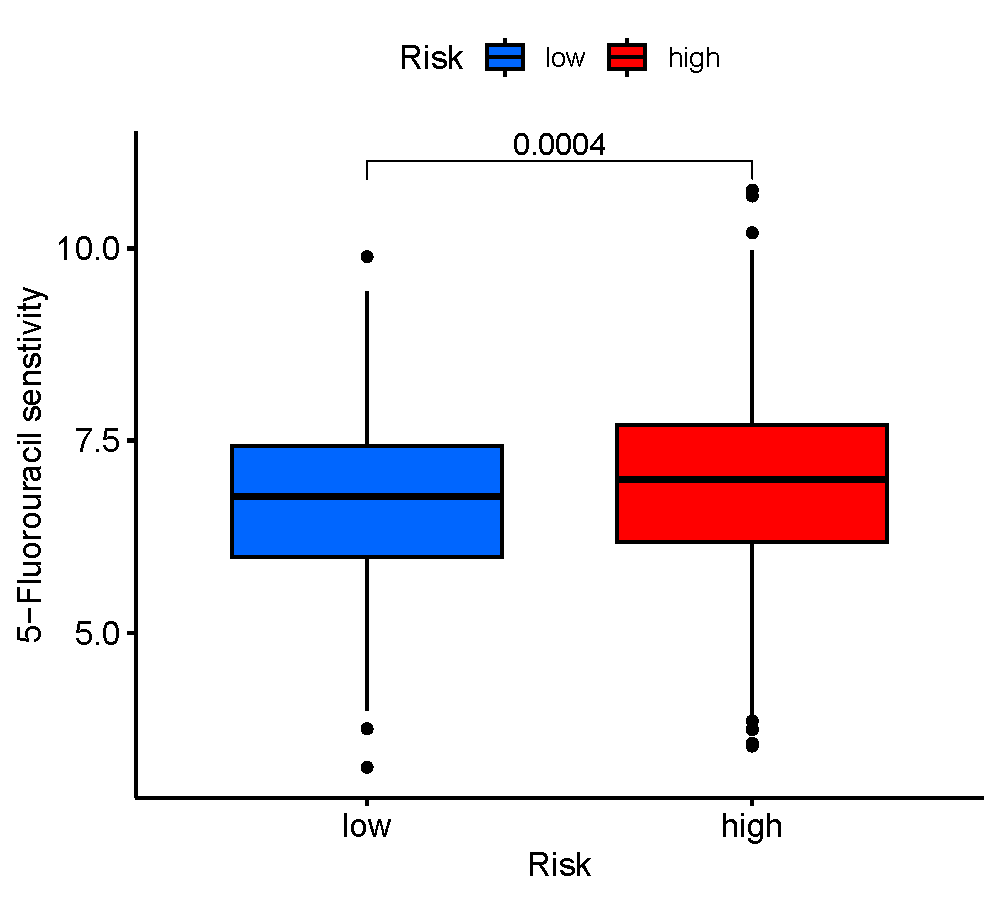

Supplement: Supplementary Figure S1 — Unsupervised clustering of disulfidptosis-related genes and Consensus matrix heatmaps for k = 3-9. [file DataSheet1.zip › Fiugre S5/drugSenstivity.5-Fluorouracil.png]

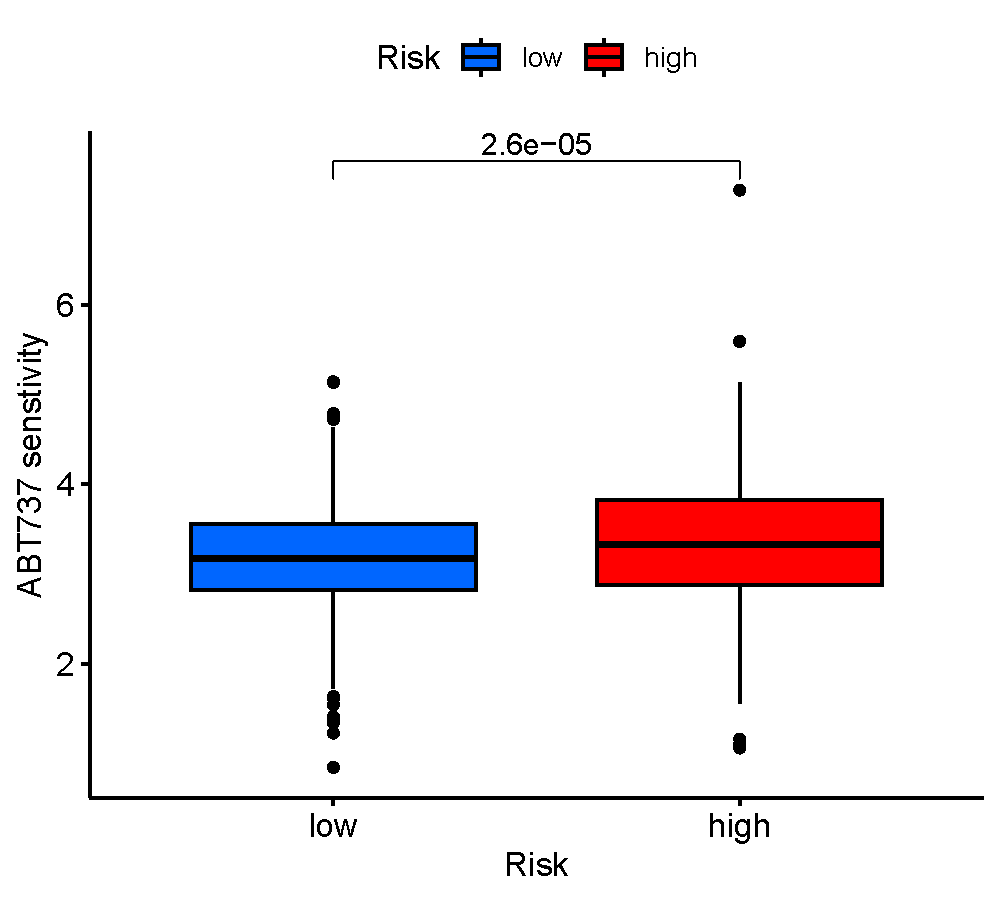

Supplement: Supplementary Figure S1 — Unsupervised clustering of disulfidptosis-related genes and Consensus matrix heatmaps for k = 3-9. [file DataSheet1.zip › Fiugre S5/drugSenstivity.ABT737.png]

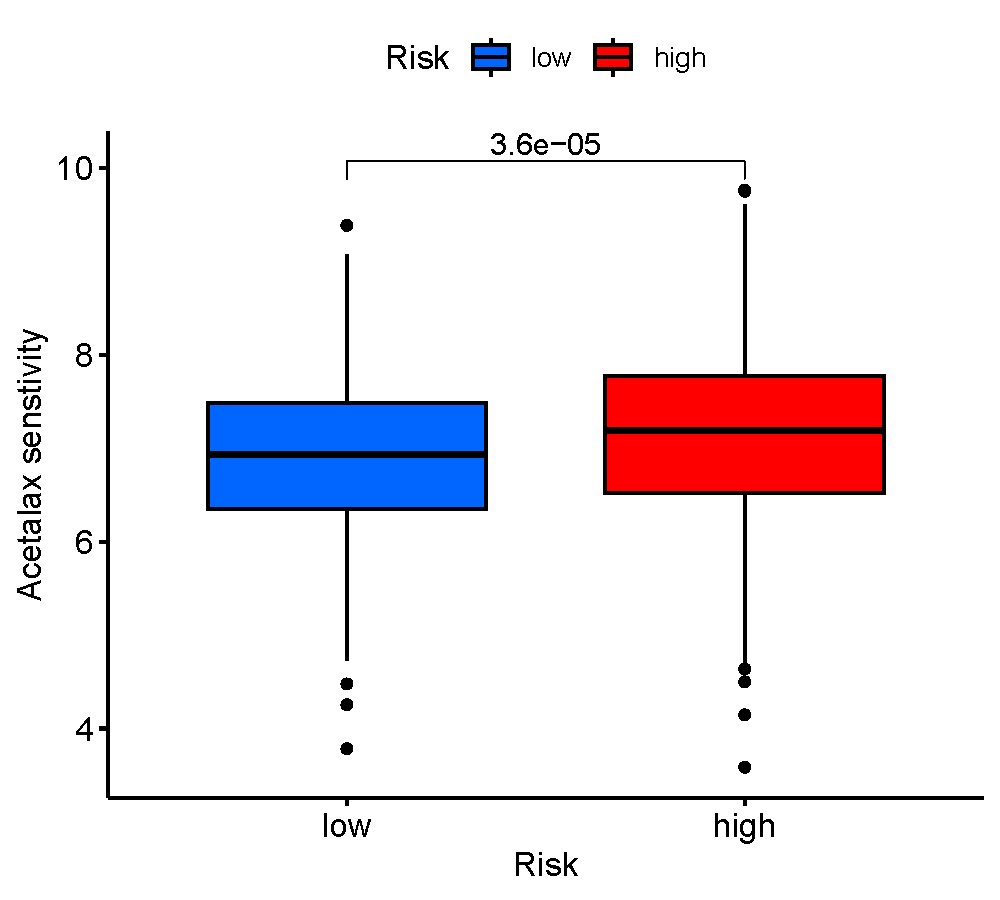

Supplement: Supplementary Figure S1 — Unsupervised clustering of disulfidptosis-related genes and Consensus matrix heatmaps for k = 3-9. [file DataSheet1.zip › Fiugre S5/drugSenstivity.Acetalax.png]

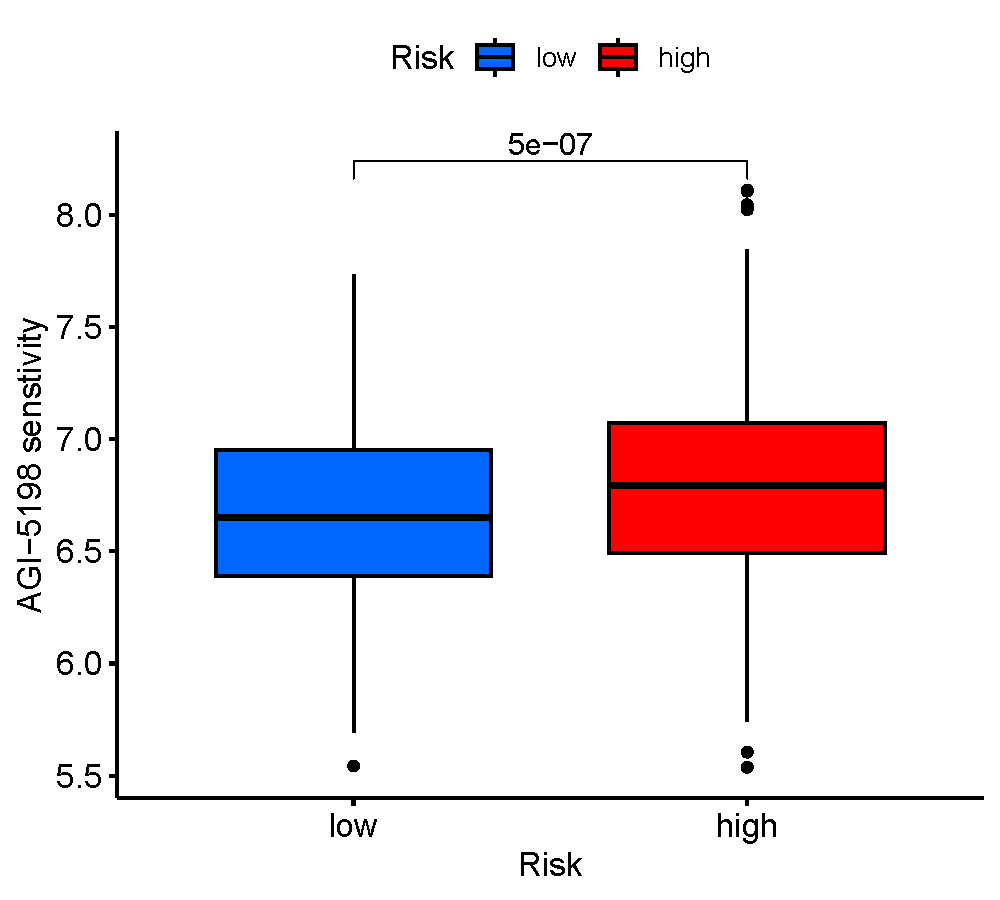

Supplement: Supplementary Figure S1 — Unsupervised clustering of disulfidptosis-related genes and Consensus matrix heatmaps for k = 3-9. [file DataSheet1.zip › Fiugre S5/drugSenstivity.AGI-5198.png]

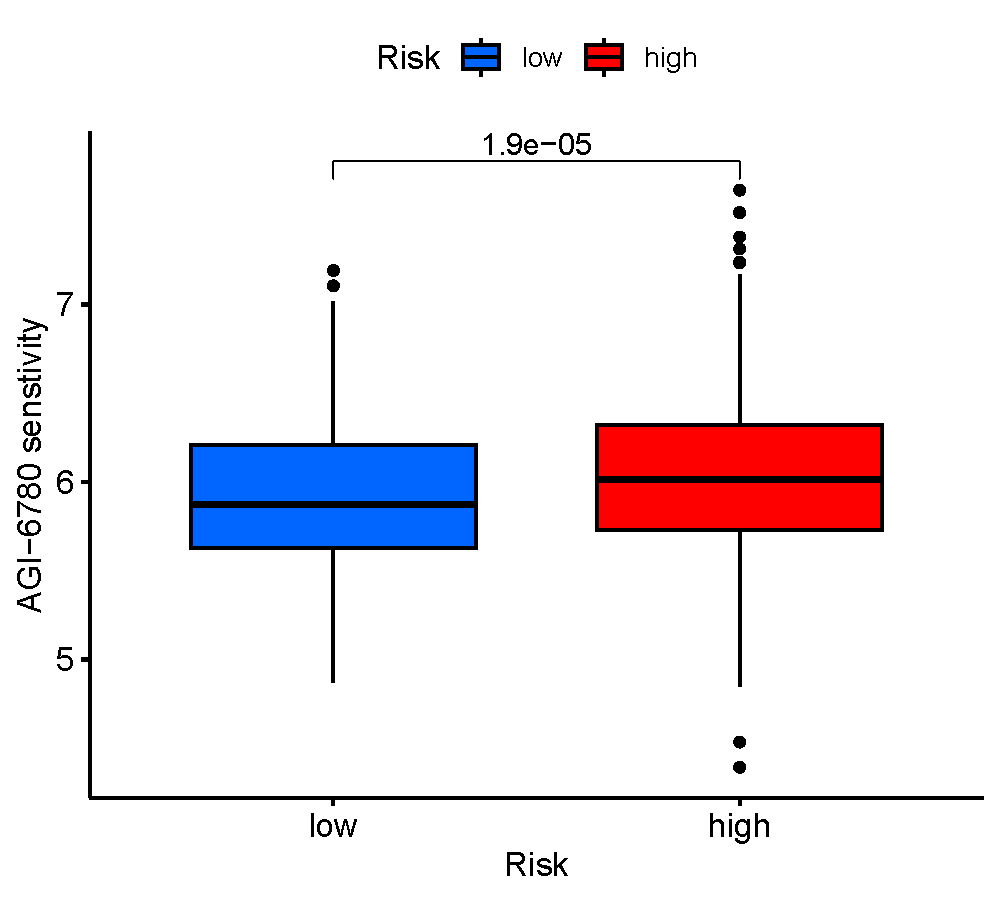

Supplement: Supplementary Figure S1 — Unsupervised clustering of disulfidptosis-related genes and Consensus matrix heatmaps for k = 3-9. [file DataSheet1.zip › Fiugre S5/drugSenstivity.AGI-6780.png]

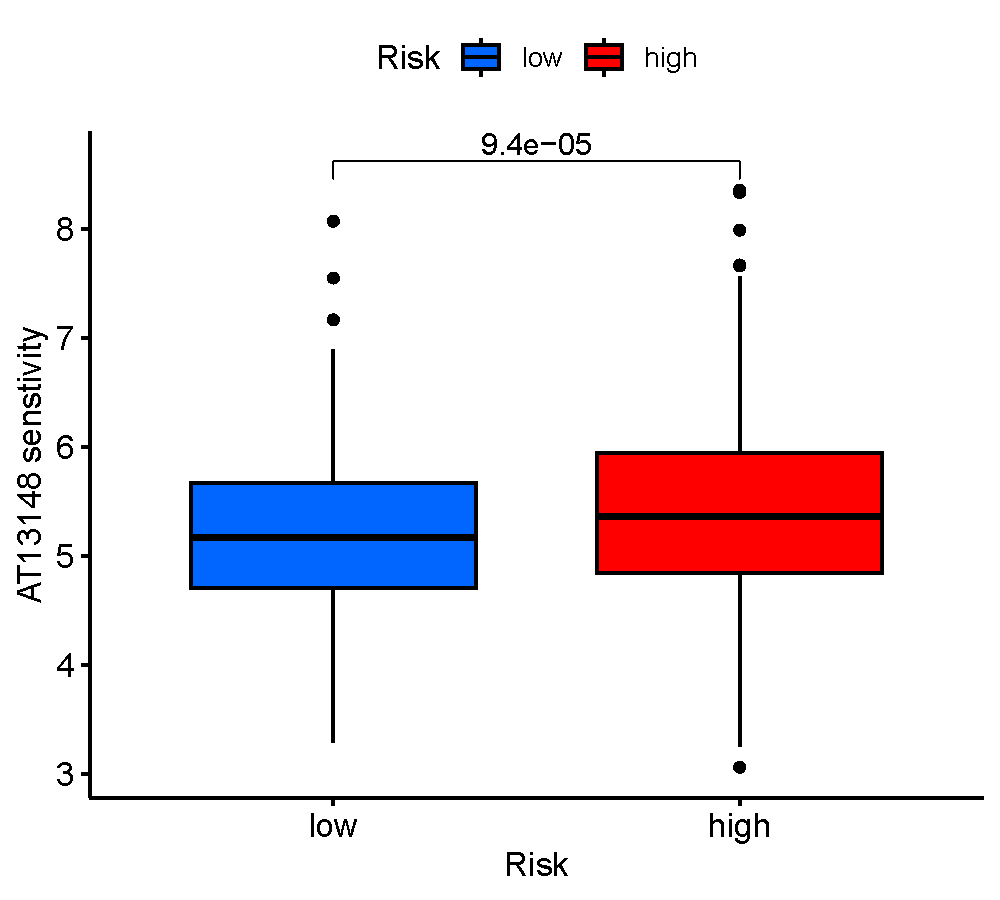

Supplement: Supplementary Figure S1 — Unsupervised clustering of disulfidptosis-related genes and Consensus matrix heatmaps for k = 3-9. [file DataSheet1.zip › Fiugre S5/drugSenstivity.AT13148.png]

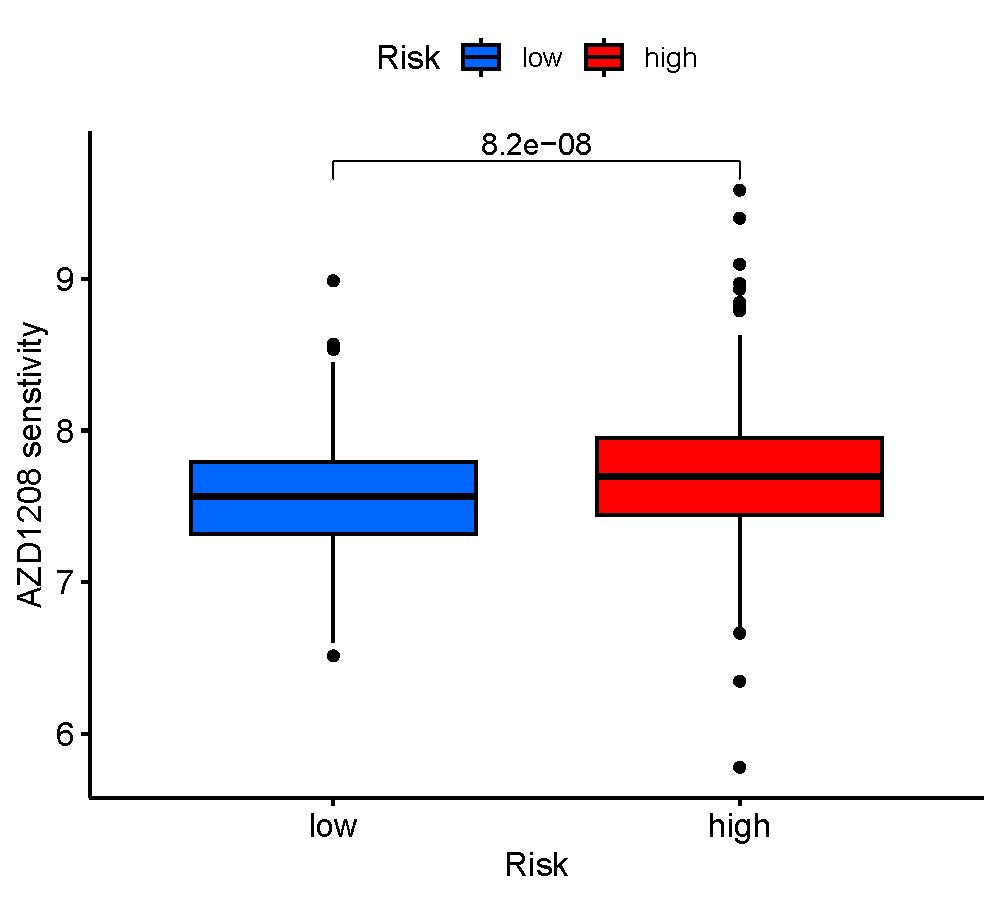

Supplement: Supplementary Figure S1 — Unsupervised clustering of disulfidptosis-related genes and Consensus matrix heatmaps for k = 3-9. [file DataSheet1.zip › Fiugre S5/drugSenstivity.AZD1208.png]

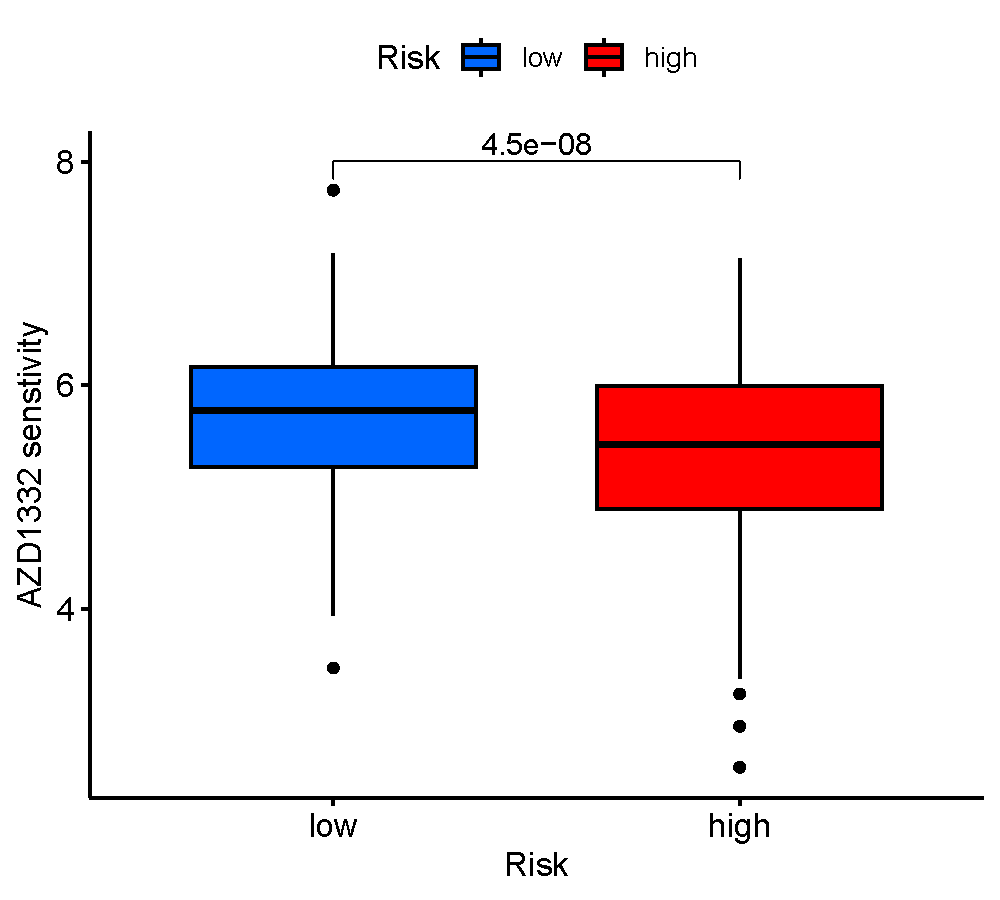

Supplement: Supplementary Figure S1 — Unsupervised clustering of disulfidptosis-related genes and Consensus matrix heatmaps for k = 3-9. [file DataSheet1.zip › Fiugre S5/drugSenstivity.AZD1332.png]

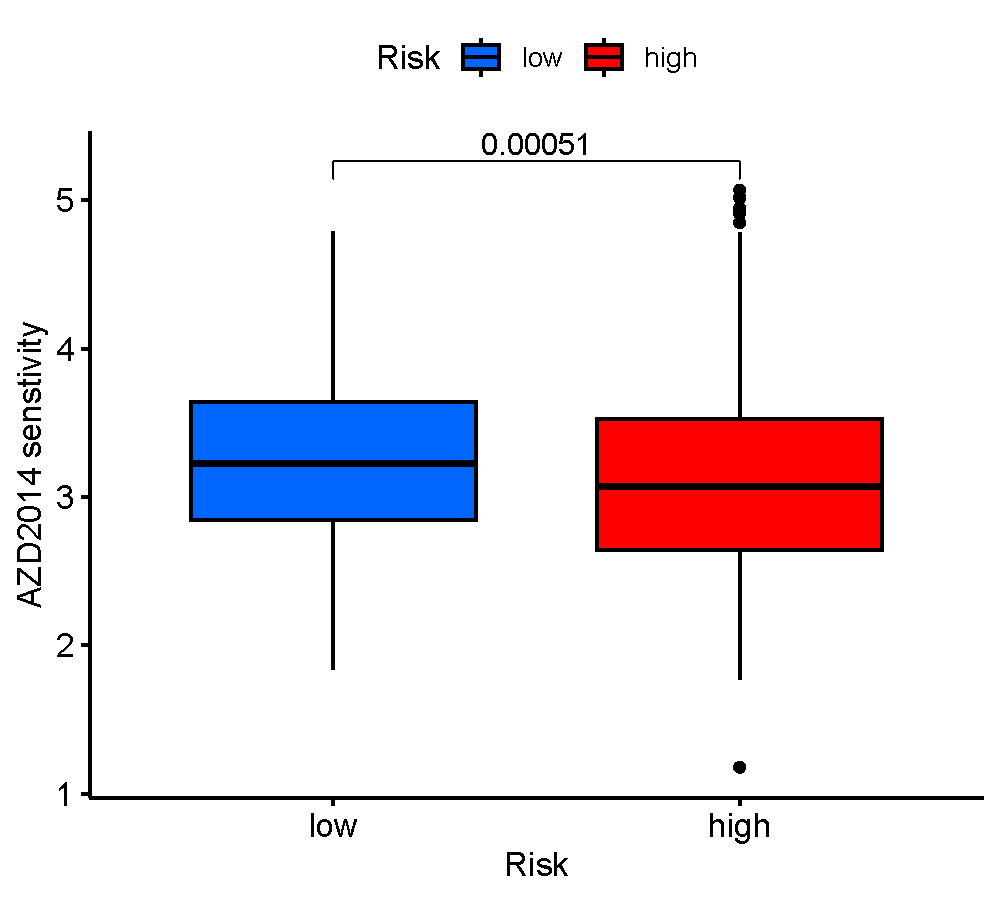

Supplement: Supplementary Figure S1 — Unsupervised clustering of disulfidptosis-related genes and Consensus matrix heatmaps for k = 3-9. [file DataSheet1.zip › Fiugre S5/drugSenstivity.AZD2014.png]

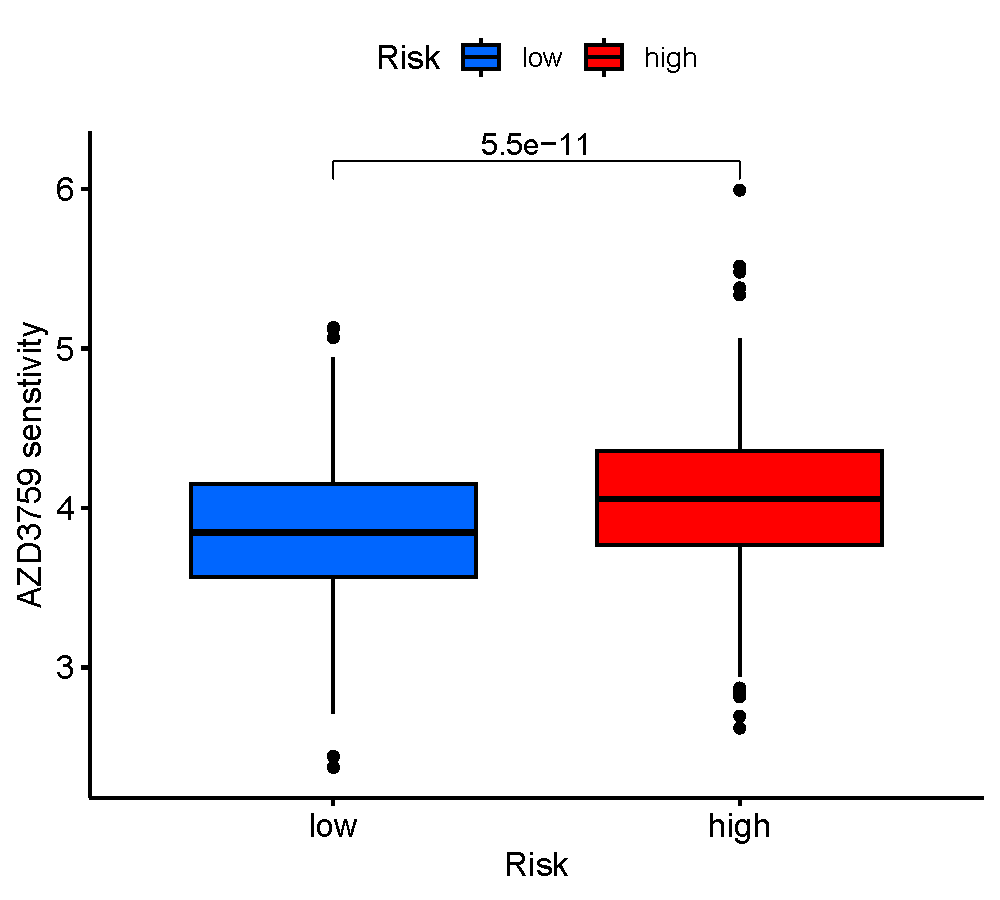

Supplement: Supplementary Figure S1 — Unsupervised clustering of disulfidptosis-related genes and Consensus matrix heatmaps for k = 3-9. [file DataSheet1.zip › Fiugre S5/drugSenstivity.AZD3759.png]

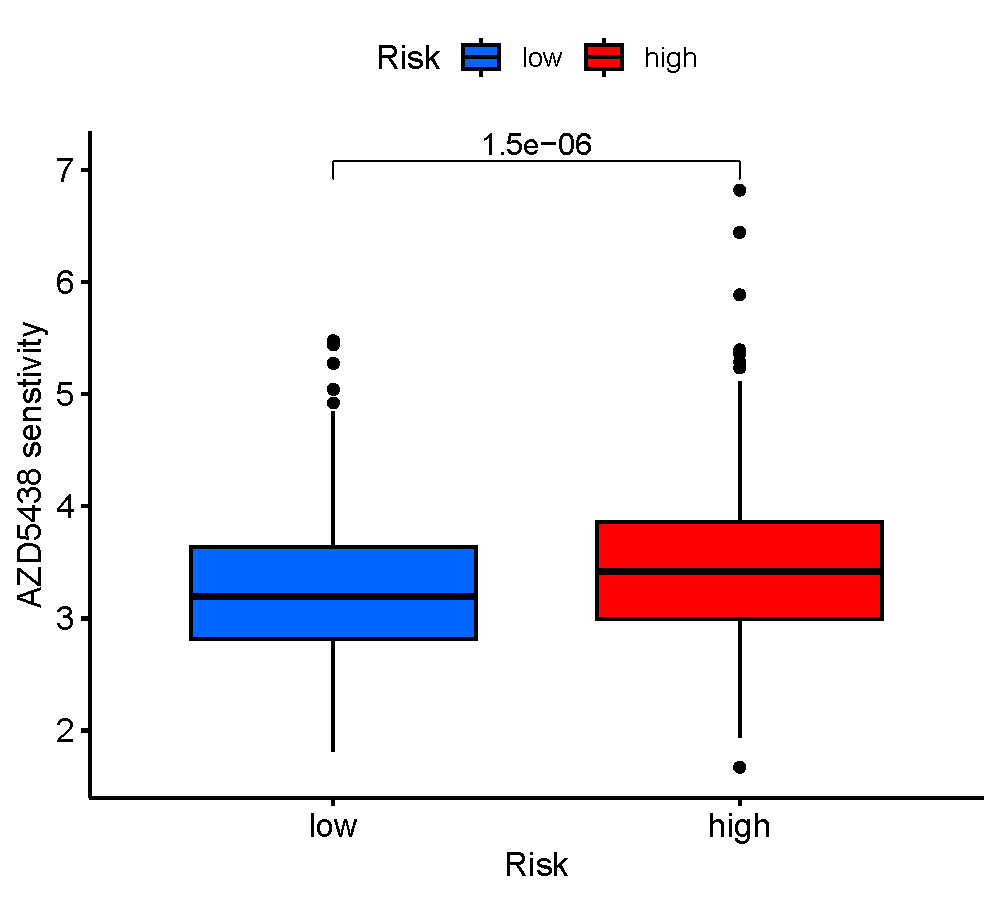

Supplement: Supplementary Figure S1 — Unsupervised clustering of disulfidptosis-related genes and Consensus matrix heatmaps for k = 3-9. [file DataSheet1.zip › Fiugre S5/drugSenstivity.AZD5438.png]

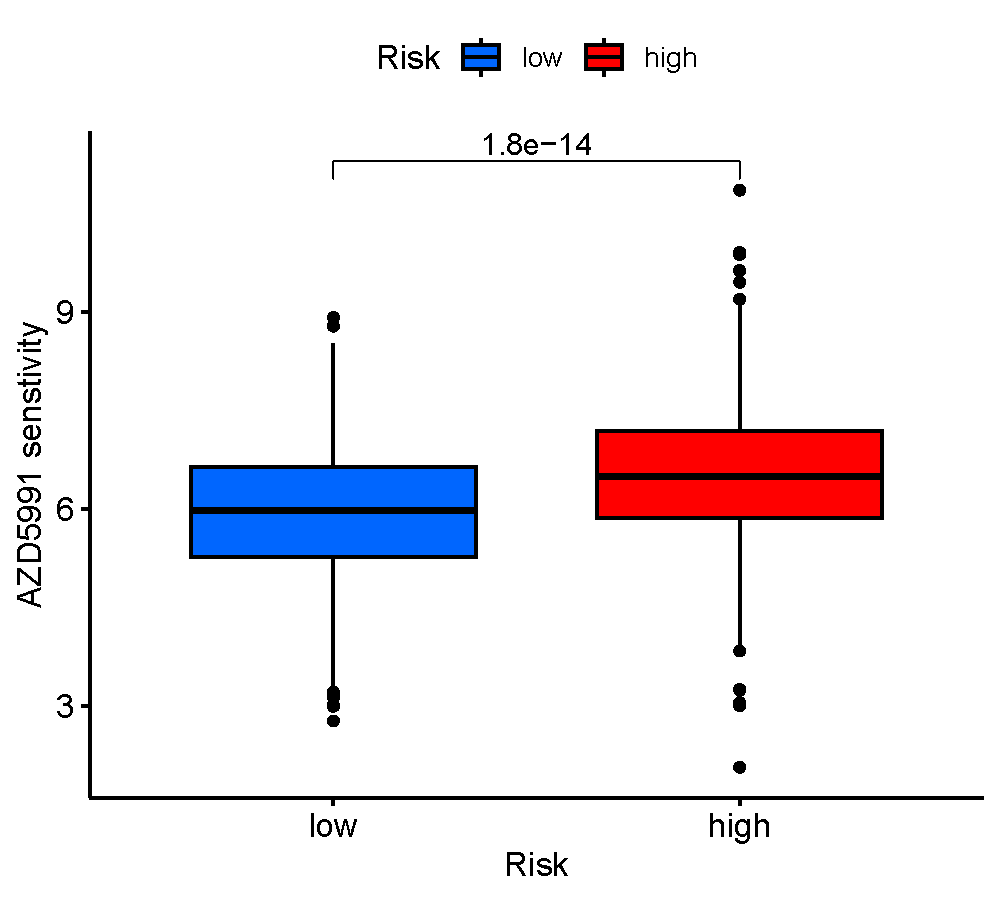

Supplement: Supplementary Figure S1 — Unsupervised clustering of disulfidptosis-related genes and Consensus matrix heatmaps for k = 3-9. [file DataSheet1.zip › Fiugre S5/drugSenstivity.AZD5991.png]

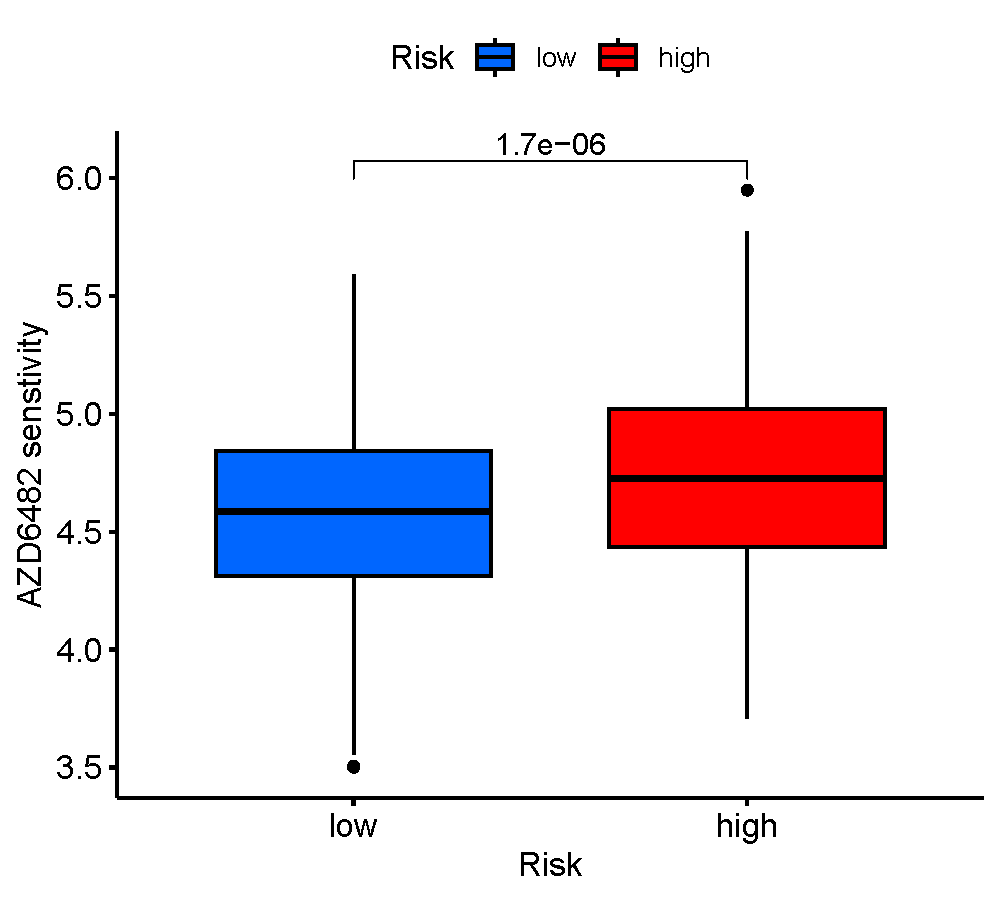

Supplement: Supplementary Figure S1 — Unsupervised clustering of disulfidptosis-related genes and Consensus matrix heatmaps for k = 3-9. [file DataSheet1.zip › Fiugre S5/drugSenstivity.AZD6482.png]

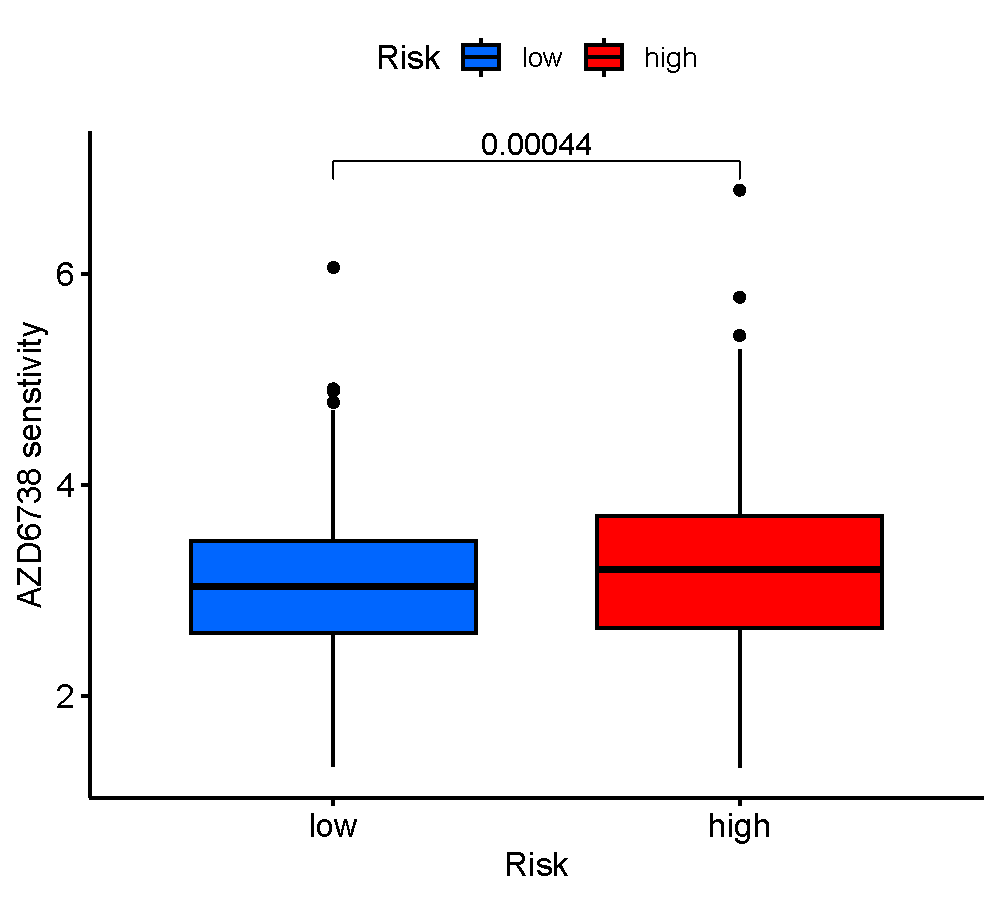

Supplement: Supplementary Figure S1 — Unsupervised clustering of disulfidptosis-related genes and Consensus matrix heatmaps for k = 3-9. [file DataSheet1.zip › Fiugre S5/drugSenstivity.AZD6738.png]

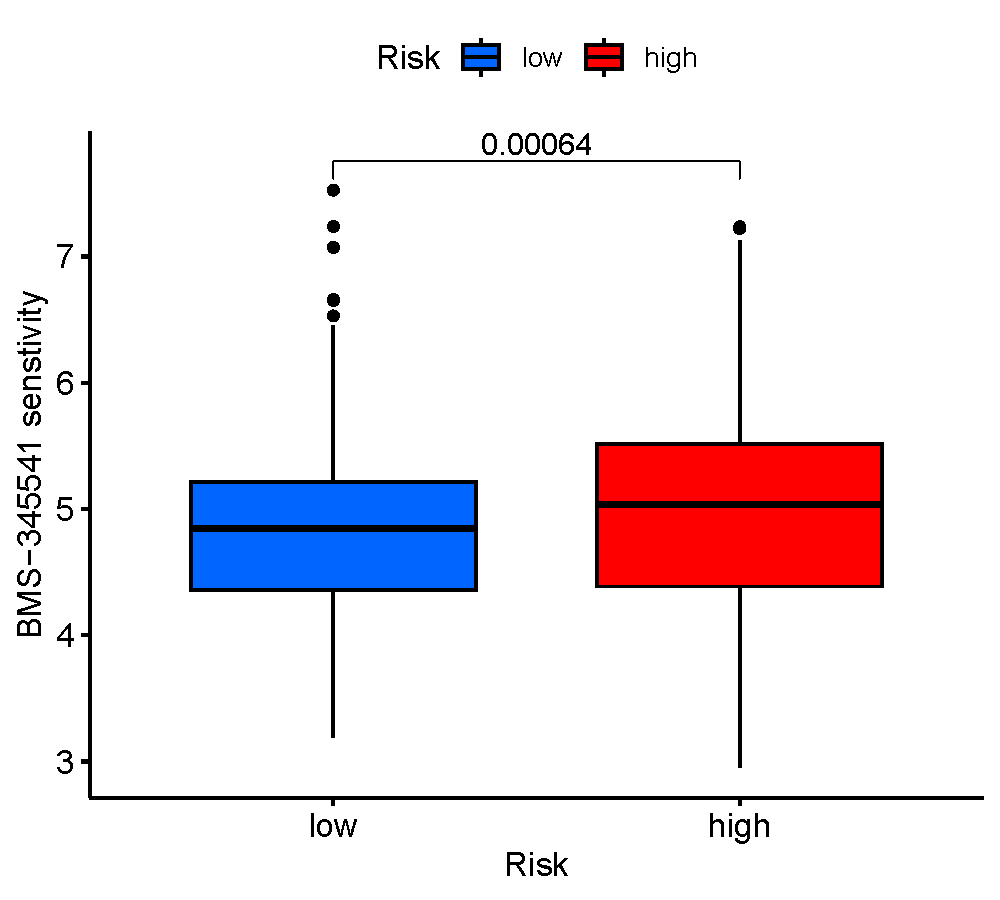

Supplement: Supplementary Figure S1 — Unsupervised clustering of disulfidptosis-related genes and Consensus matrix heatmaps for k = 3-9. [file DataSheet1.zip › Fiugre S5/drugSenstivity.BMS-345541.png]

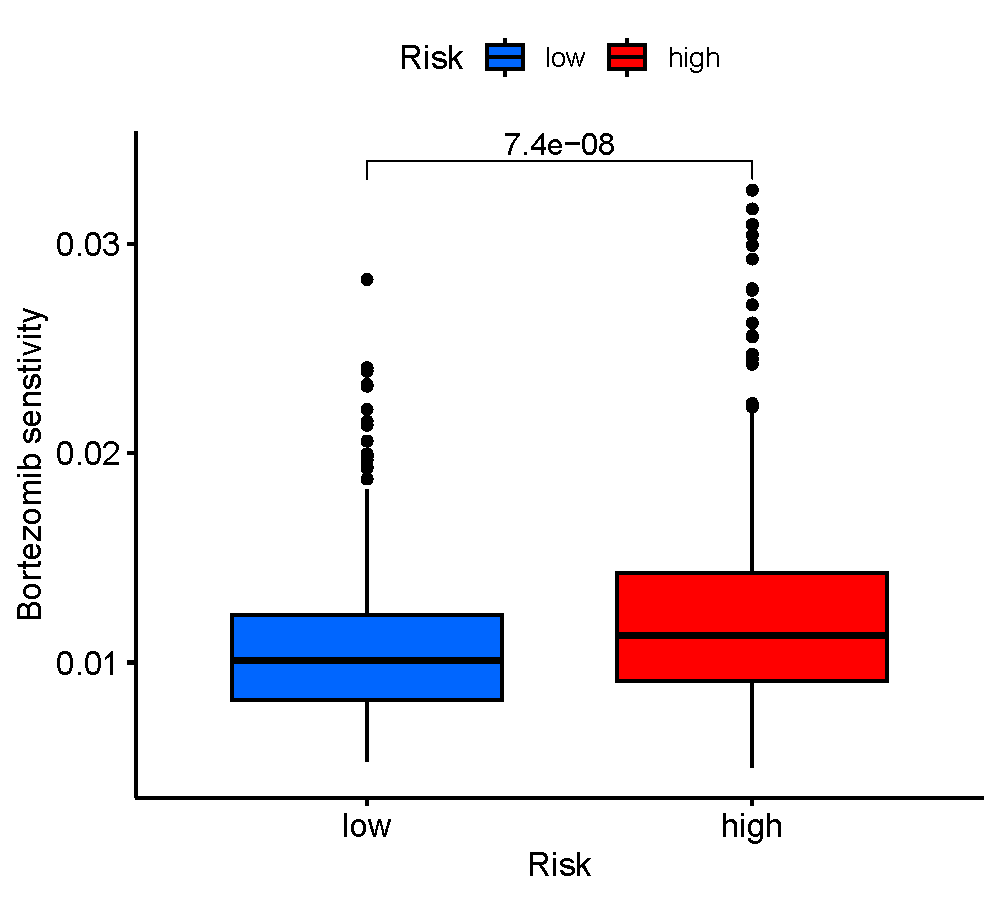

Supplement: Supplementary Figure S1 — Unsupervised clustering of disulfidptosis-related genes and Consensus matrix heatmaps for k = 3-9. [file DataSheet1.zip › Fiugre S5/drugSenstivity.Bortezomib.png]

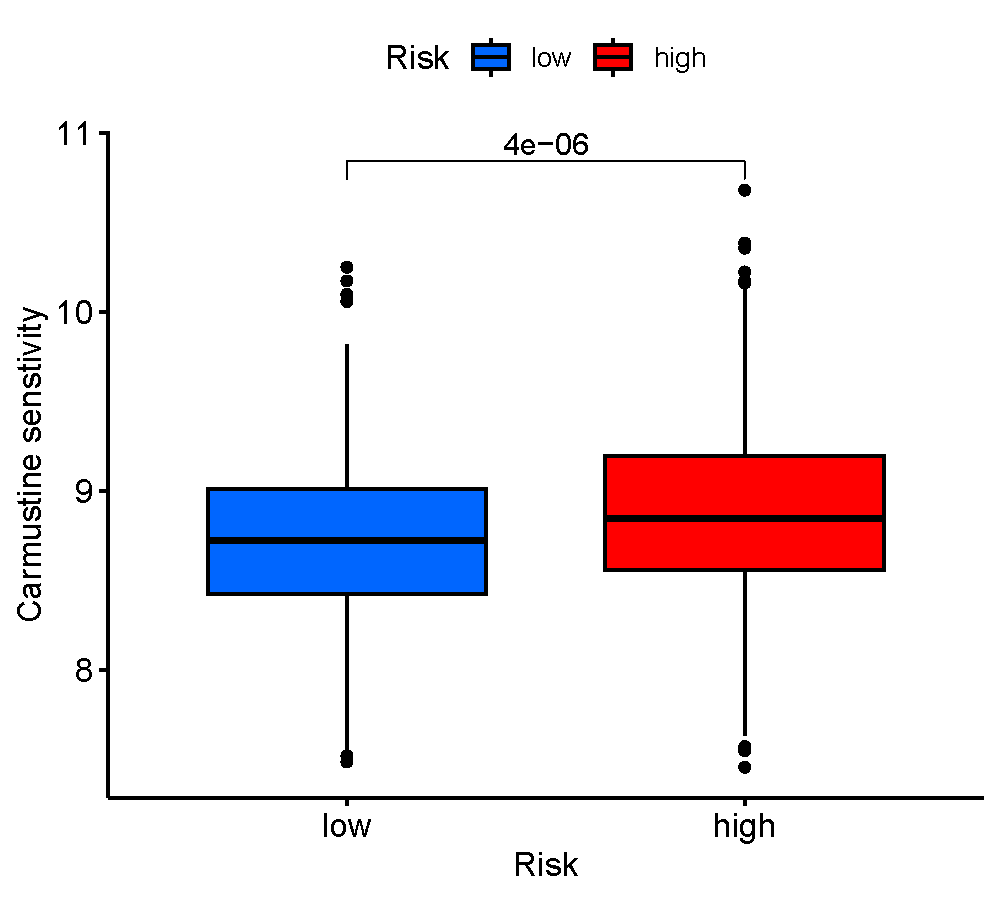

Supplement: Supplementary Figure S1 — Unsupervised clustering of disulfidptosis-related genes and Consensus matrix heatmaps for k = 3-9. [file DataSheet1.zip › Fiugre S5/drugSenstivity.Carmustine.png]

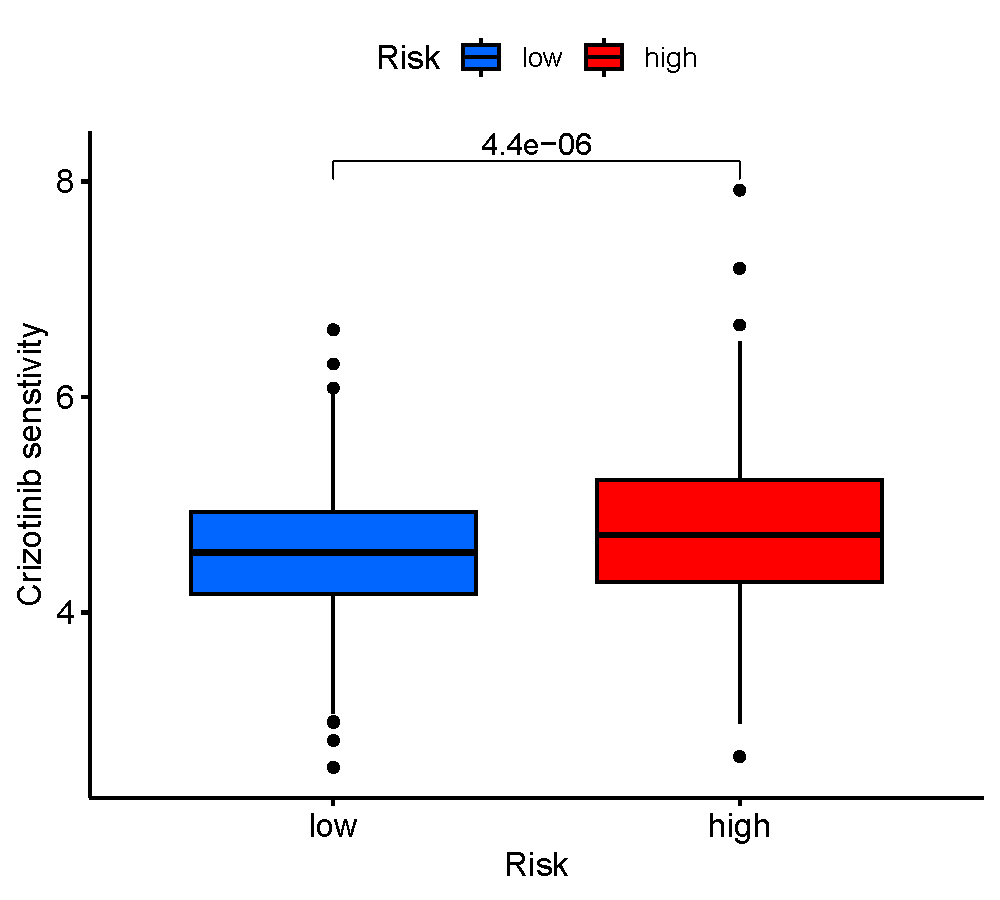

Supplement: Supplementary Figure S1 — Unsupervised clustering of disulfidptosis-related genes and Consensus matrix heatmaps for k = 3-9. [file DataSheet1.zip › Fiugre S5/drugSenstivity.Crizotinib.png]

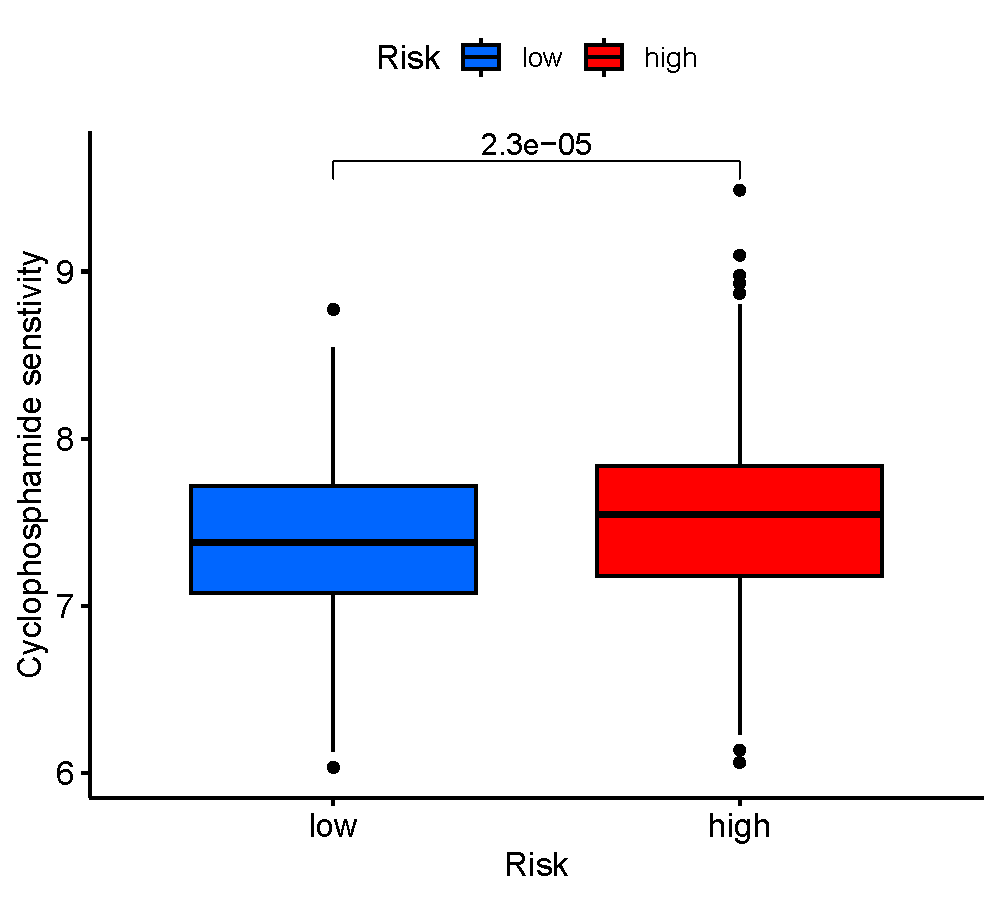

Supplement: Supplementary Figure S1 — Unsupervised clustering of disulfidptosis-related genes and Consensus matrix heatmaps for k = 3-9. [file DataSheet1.zip › Fiugre S5/drugSenstivity.Cyclophosphamide.png]

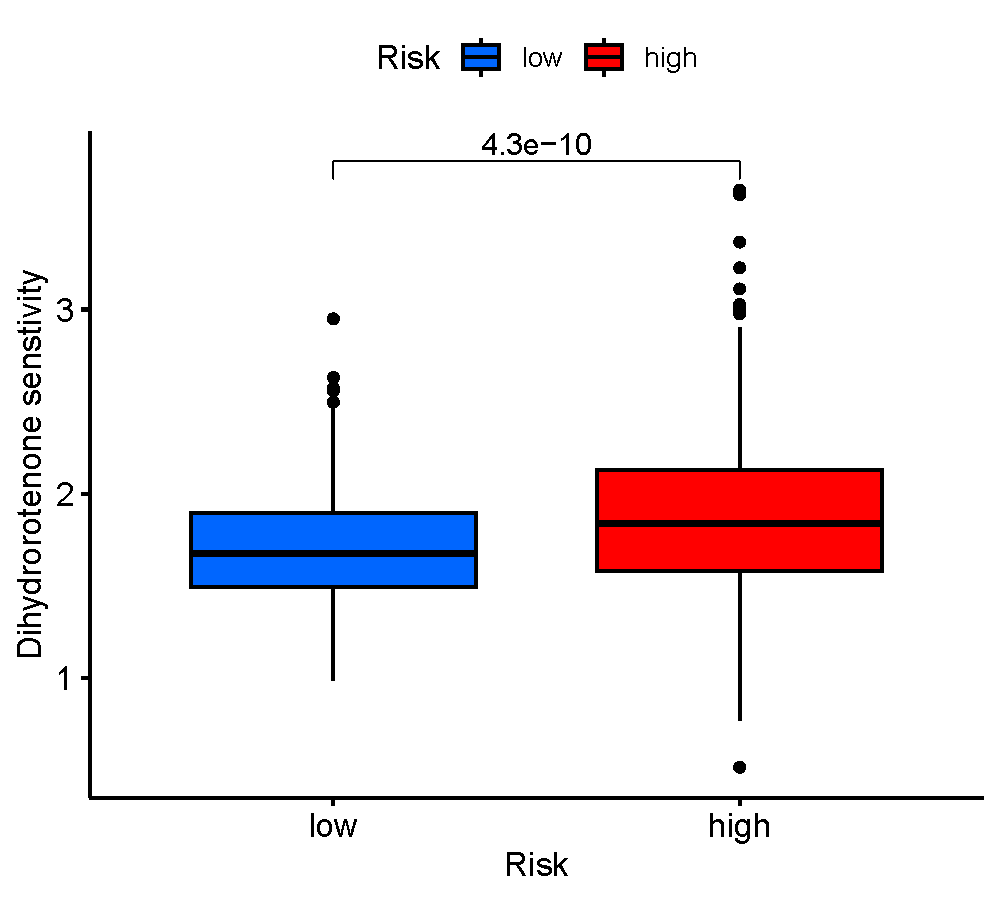

Supplement: Supplementary Figure S1 — Unsupervised clustering of disulfidptosis-related genes and Consensus matrix heatmaps for k = 3-9. [file DataSheet1.zip › Fiugre S5/drugSenstivity.Dihydrorotenone.png]

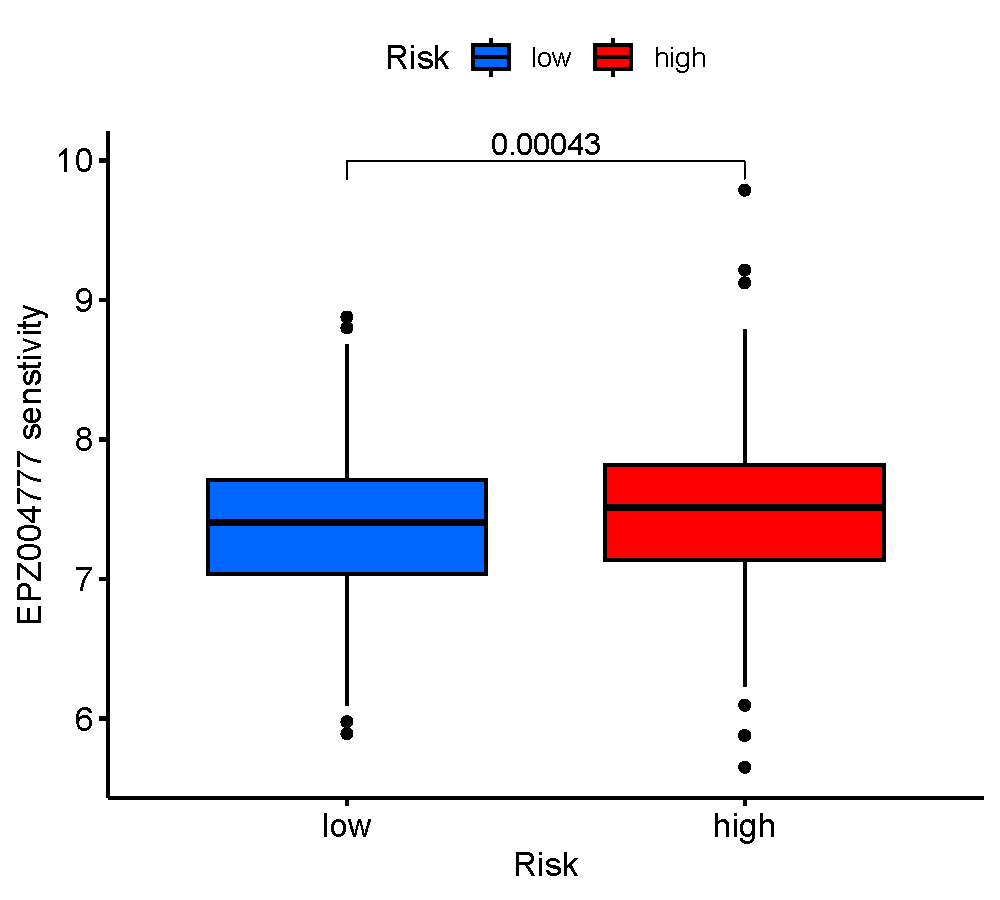

Supplement: Supplementary Figure S1 — Unsupervised clustering of disulfidptosis-related genes and Consensus matrix heatmaps for k = 3-9. [file DataSheet1.zip › Fiugre S5/drugSenstivity.EPZ004777.png]

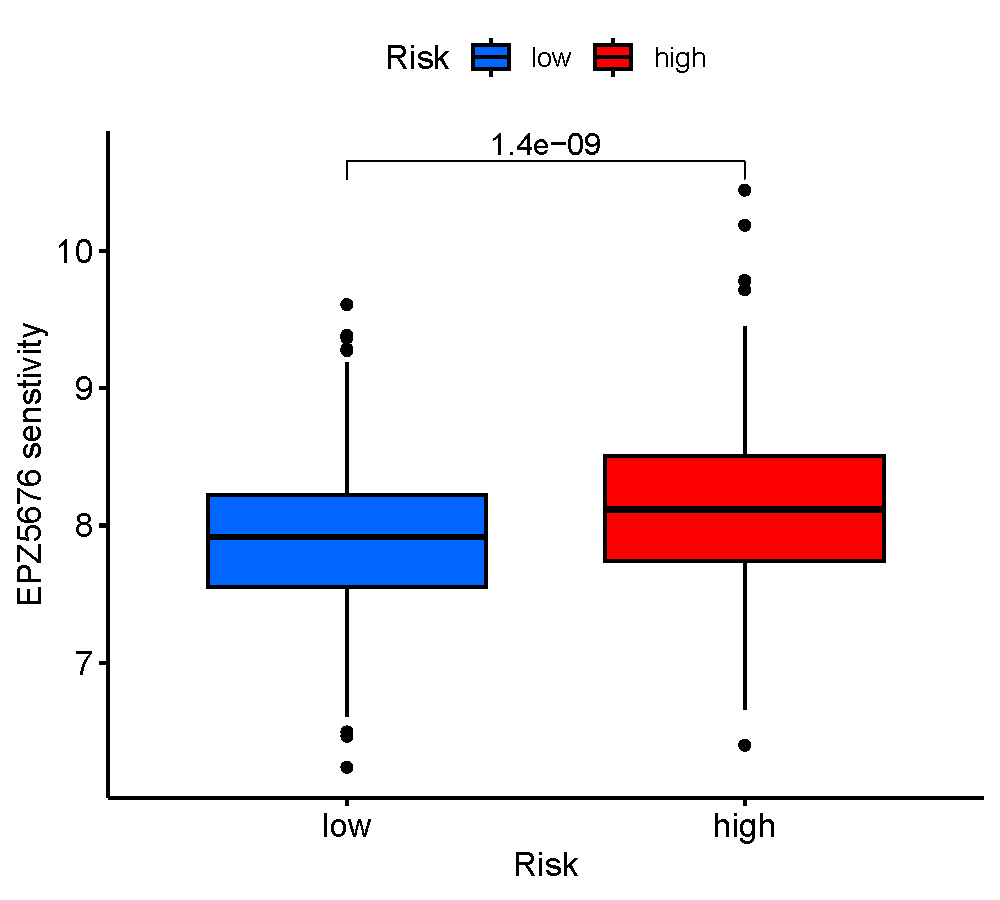

Supplement: Supplementary Figure S1 — Unsupervised clustering of disulfidptosis-related genes and Consensus matrix heatmaps for k = 3-9. [file DataSheet1.zip › Fiugre S5/drugSenstivity.EPZ5676.png]

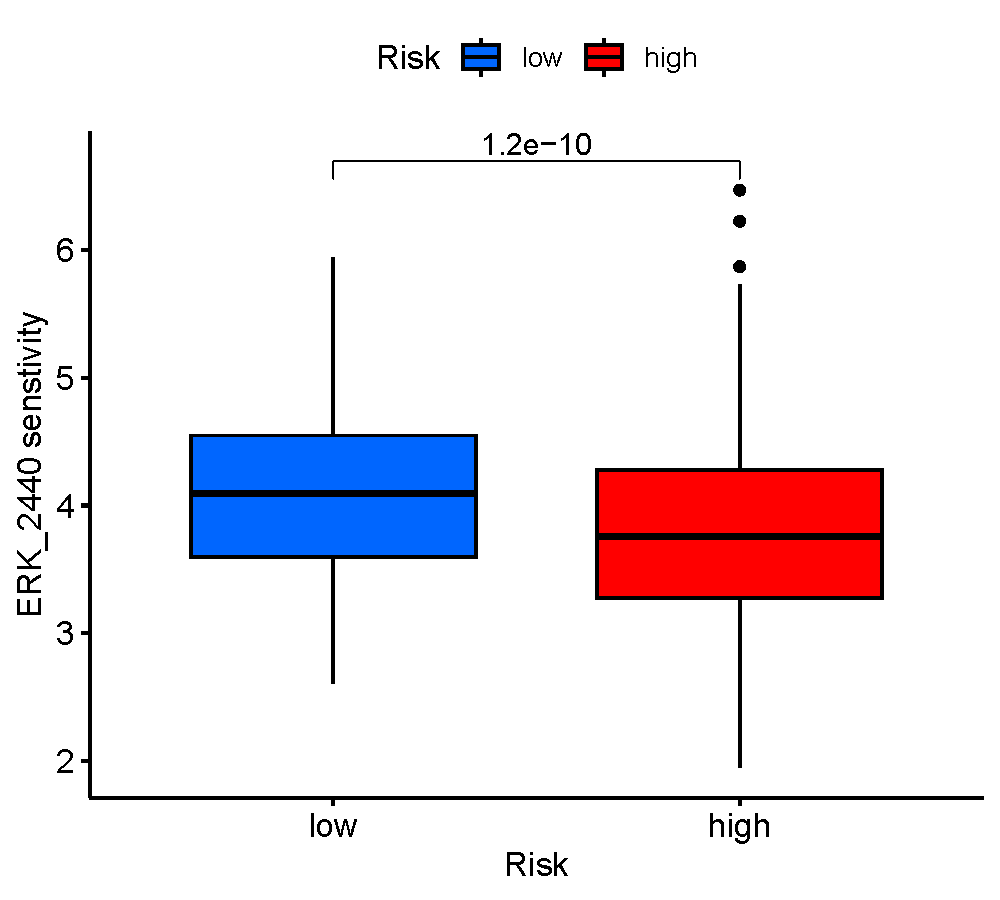

Supplement: Supplementary Figure S1 — Unsupervised clustering of disulfidptosis-related genes and Consensus matrix heatmaps for k = 3-9. [file DataSheet1.zip › Fiugre S5/drugSenstivity.ERK_2440.png]

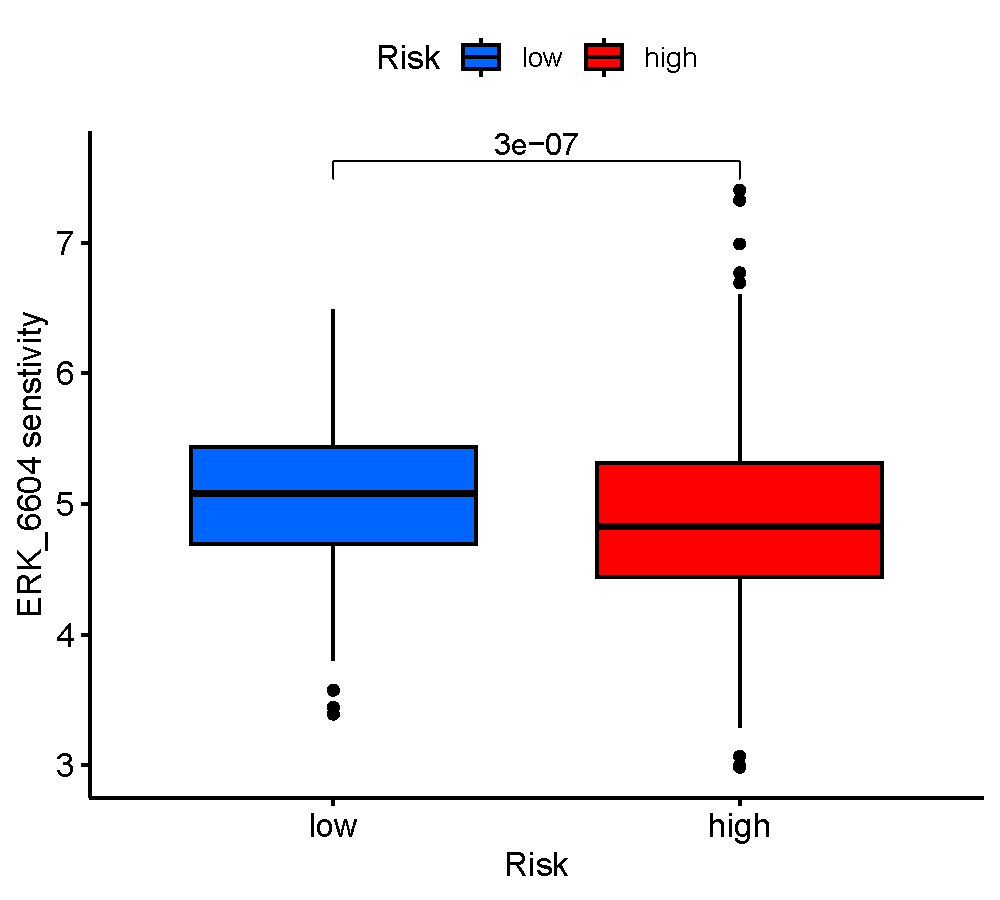

Supplement: Supplementary Figure S1 — Unsupervised clustering of disulfidptosis-related genes and Consensus matrix heatmaps for k = 3-9. [file DataSheet1.zip › Fiugre S5/drugSenstivity.ERK_6604.png]

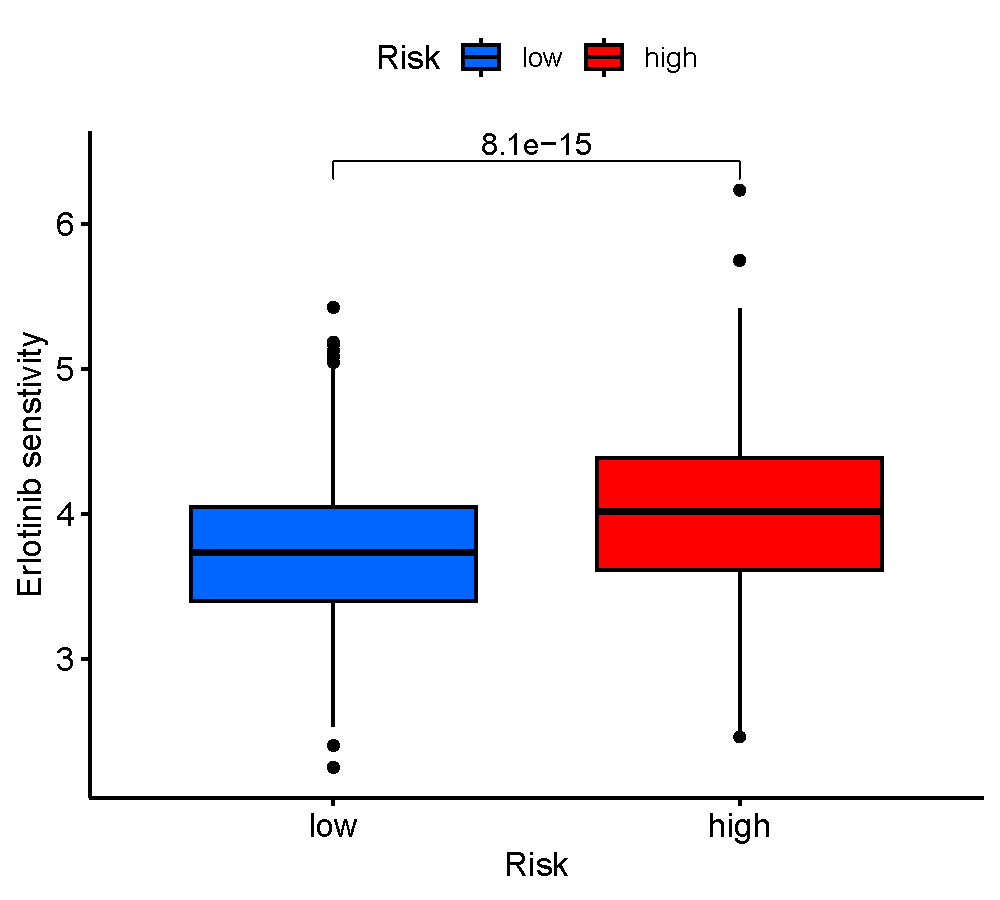

Supplement: Supplementary Figure S1 — Unsupervised clustering of disulfidptosis-related genes and Consensus matrix heatmaps for k = 3-9. [file DataSheet1.zip › Fiugre S5/drugSenstivity.Erlotinib.png]

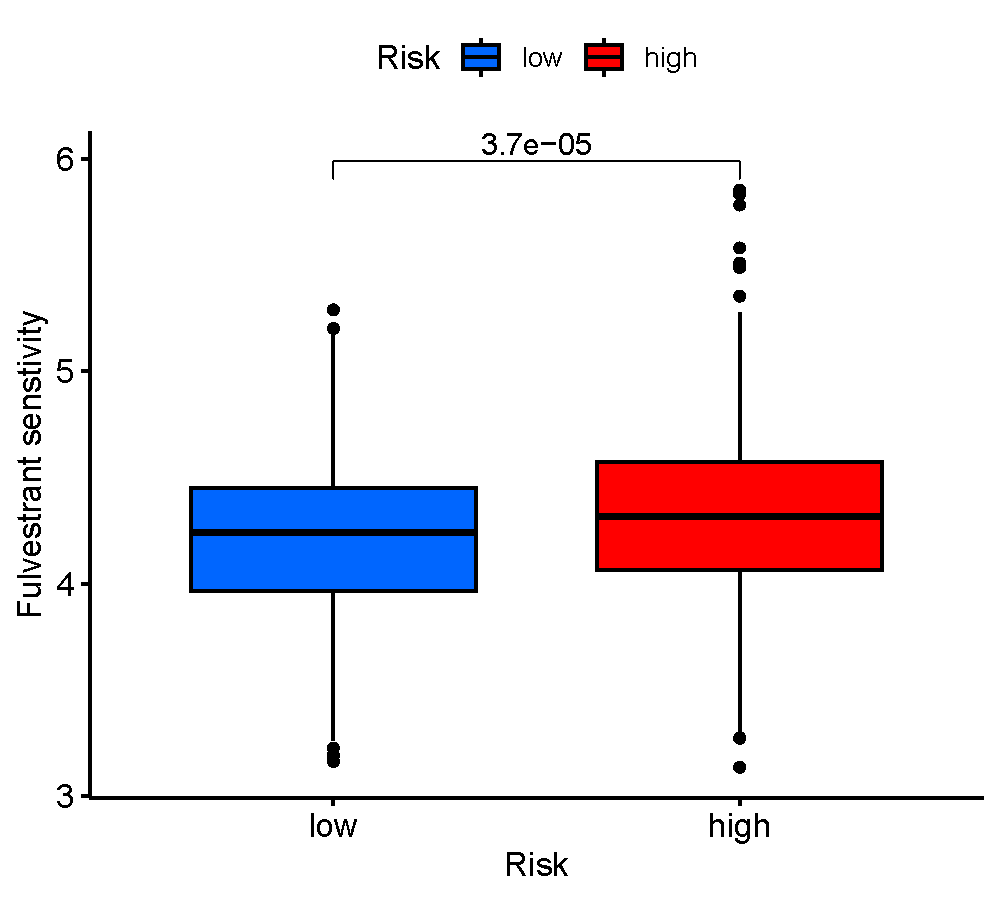

Supplement: Supplementary Figure S1 — Unsupervised clustering of disulfidptosis-related genes and Consensus matrix heatmaps for k = 3-9. [file DataSheet1.zip › Fiugre S5/drugSenstivity.Fulvestrant.png]

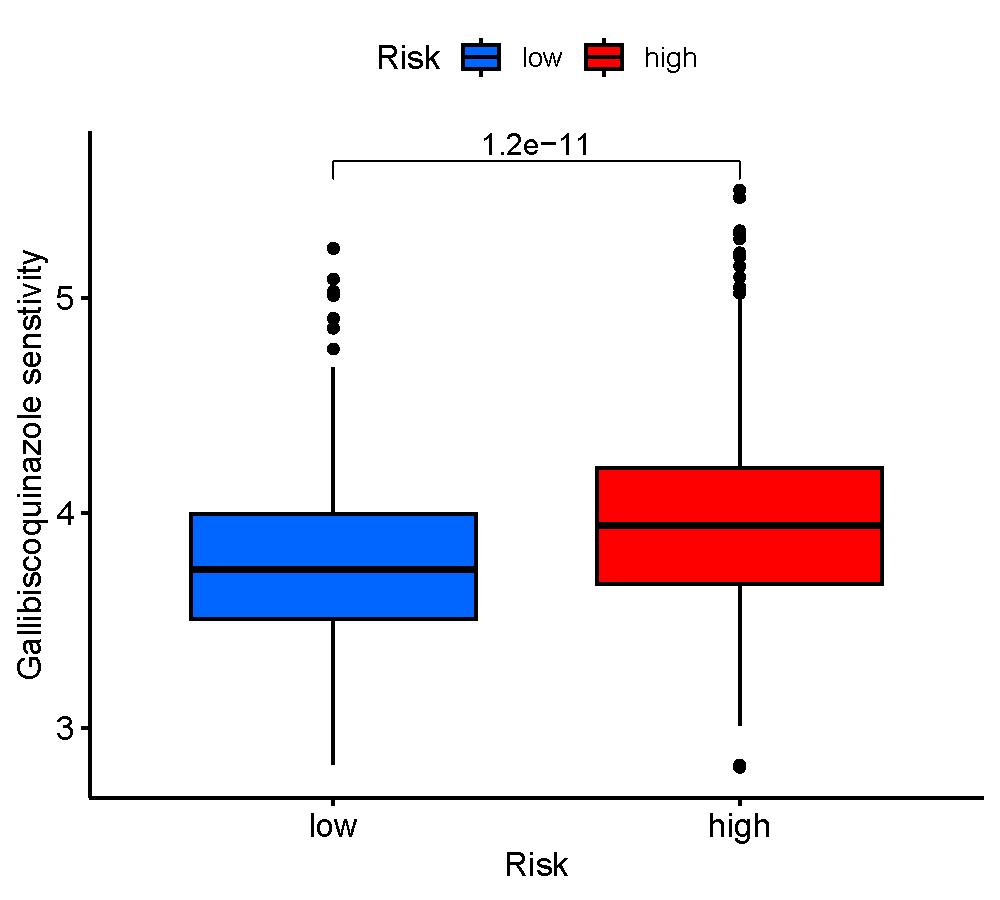

Supplement: Supplementary Figure S1 — Unsupervised clustering of disulfidptosis-related genes and Consensus matrix heatmaps for k = 3-9. [file DataSheet1.zip › Fiugre S5/drugSenstivity.Gallibiscoquinazole.png]

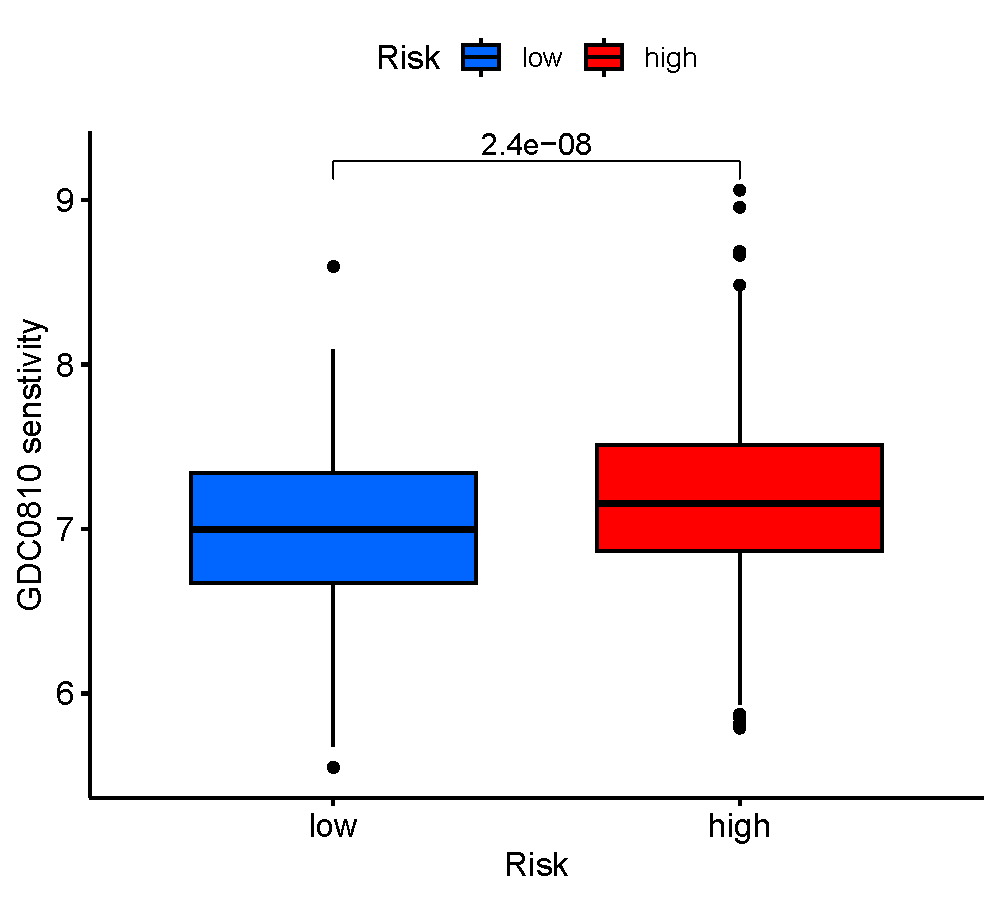

Supplement: Supplementary Figure S1 — Unsupervised clustering of disulfidptosis-related genes and Consensus matrix heatmaps for k = 3-9. [file DataSheet1.zip › Fiugre S5/drugSenstivity.GDC0810.png]

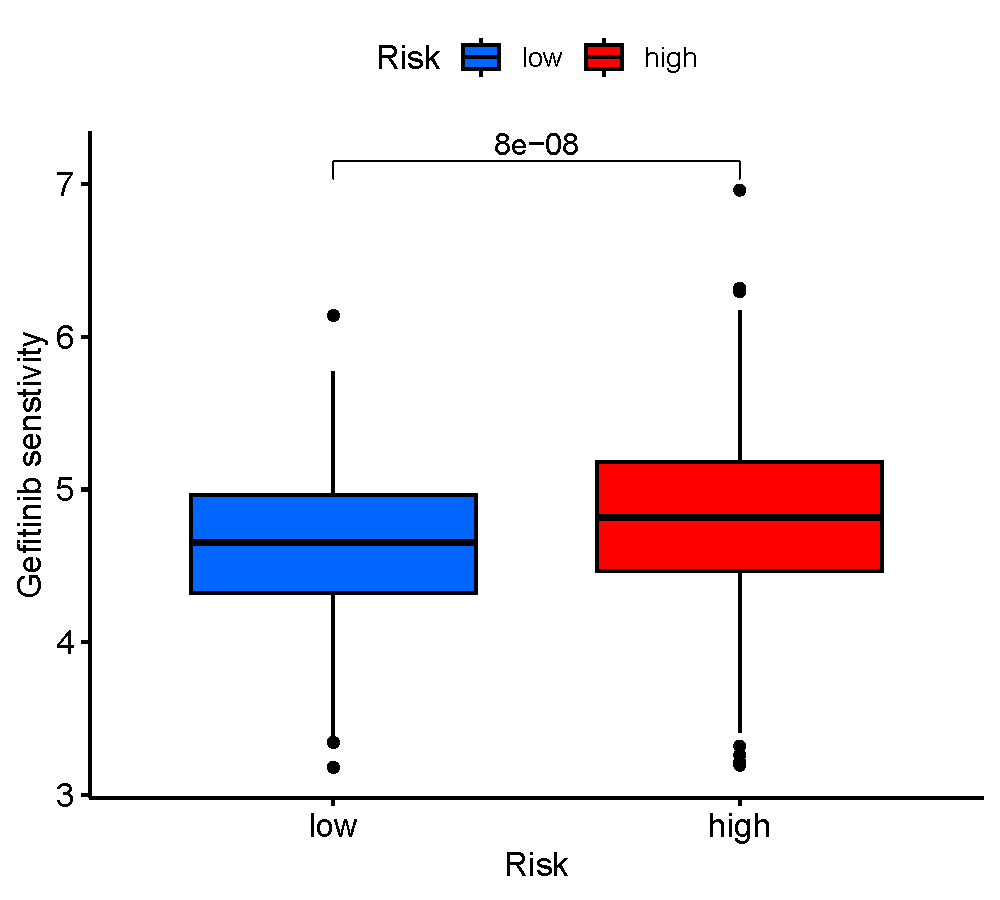

Supplement: Supplementary Figure S1 — Unsupervised clustering of disulfidptosis-related genes and Consensus matrix heatmaps for k = 3-9. [file DataSheet1.zip › Fiugre S5/drugSenstivity.Gefitinib.png]

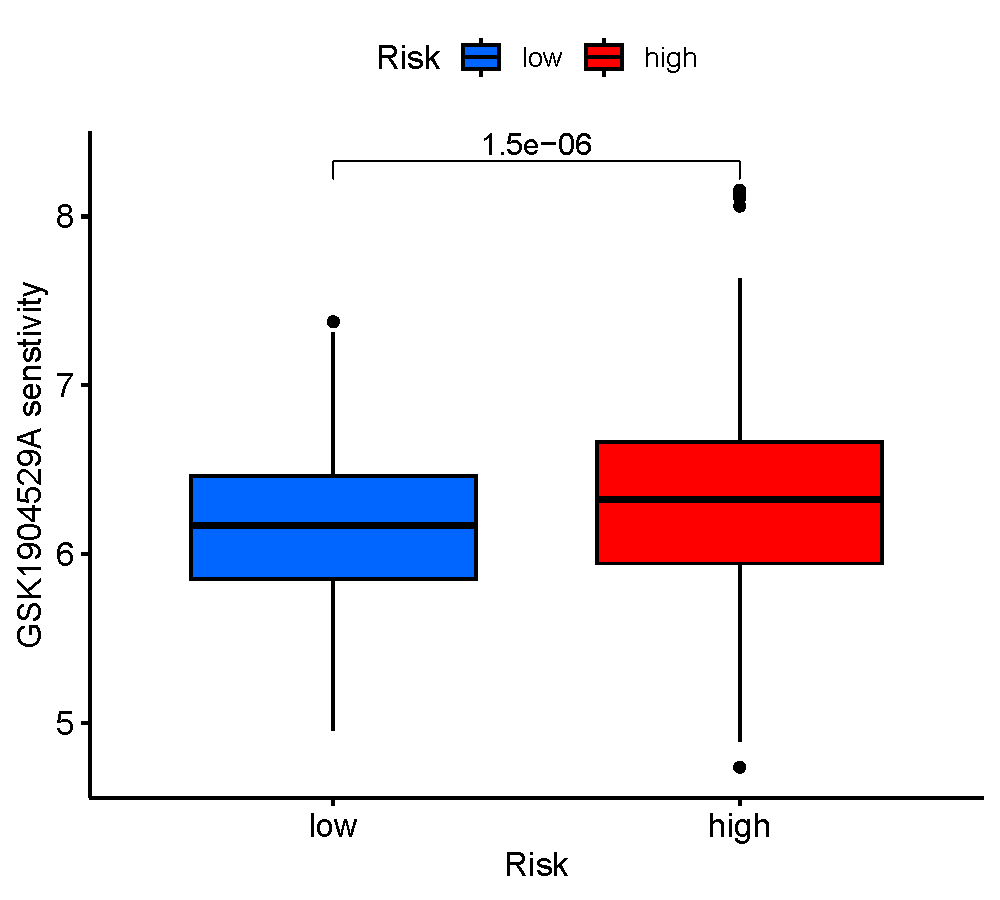

Supplement: Supplementary Figure S1 — Unsupervised clustering of disulfidptosis-related genes and Consensus matrix heatmaps for k = 3-9. [file DataSheet1.zip › Fiugre S5/drugSenstivity.GSK1904529A.png]

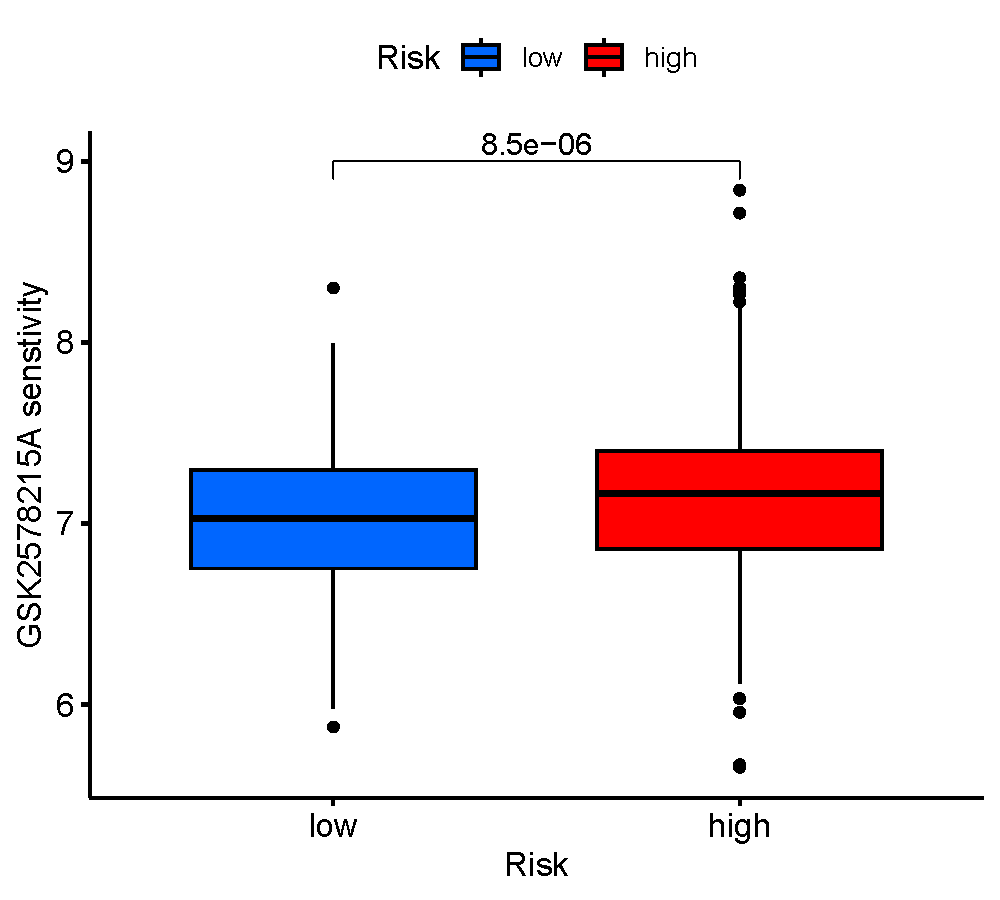

Supplement: Supplementary Figure S1 — Unsupervised clustering of disulfidptosis-related genes and Consensus matrix heatmaps for k = 3-9. [file DataSheet1.zip › Fiugre S5/drugSenstivity.GSK2578215A.png]

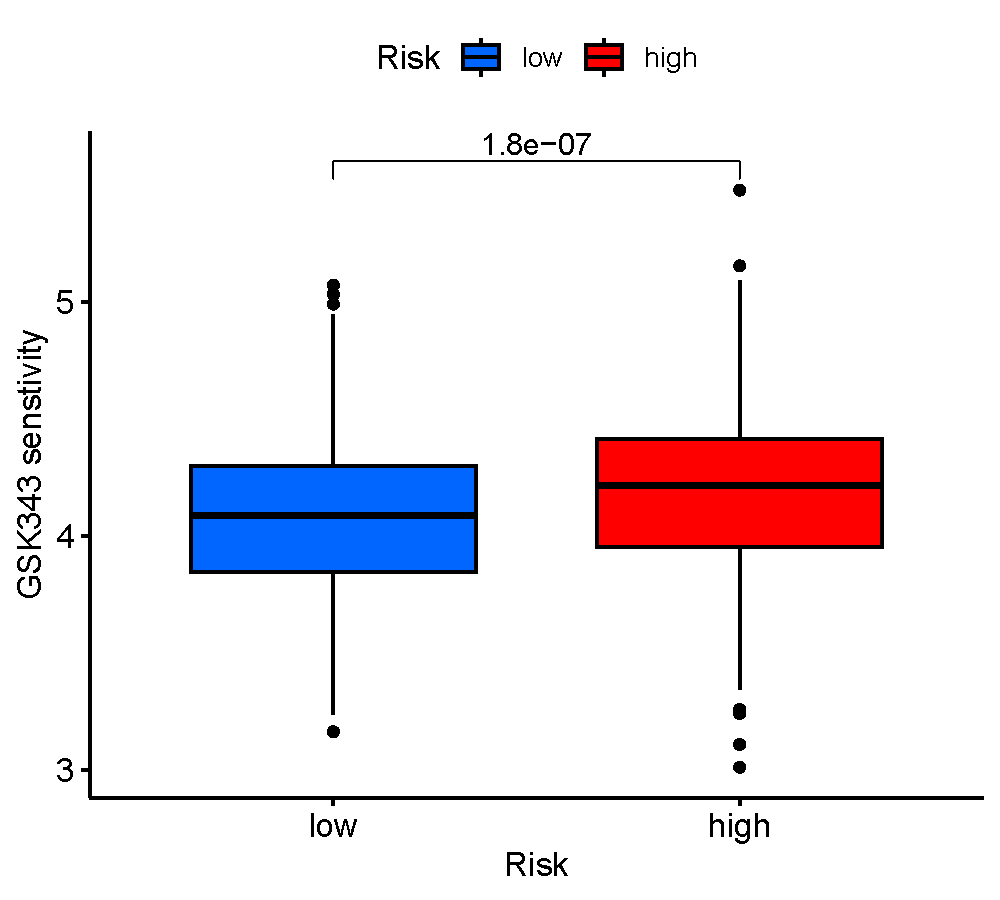

Supplement: Supplementary Figure S1 — Unsupervised clustering of disulfidptosis-related genes and Consensus matrix heatmaps for k = 3-9. [file DataSheet1.zip › Fiugre S5/drugSenstivity.GSK343.png]

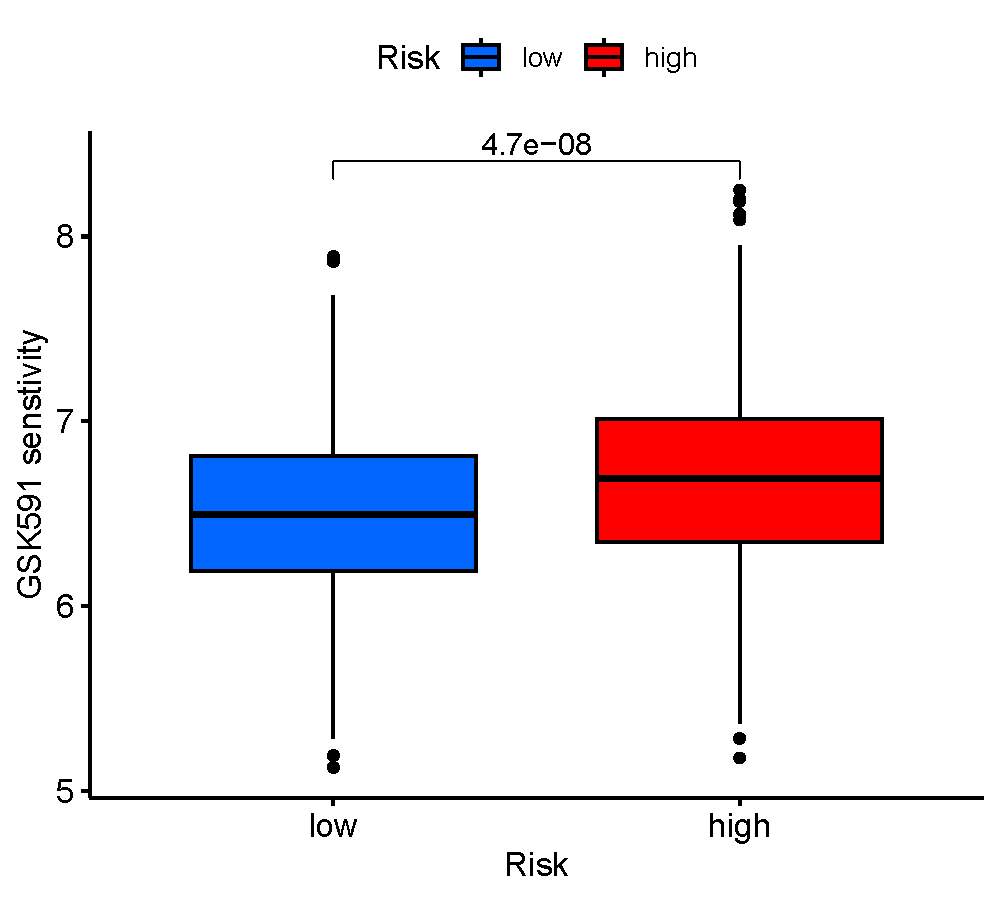

Supplement: Supplementary Figure S1 — Unsupervised clustering of disulfidptosis-related genes and Consensus matrix heatmaps for k = 3-9. [file DataSheet1.zip › Fiugre S5/drugSenstivity.GSK591.png]

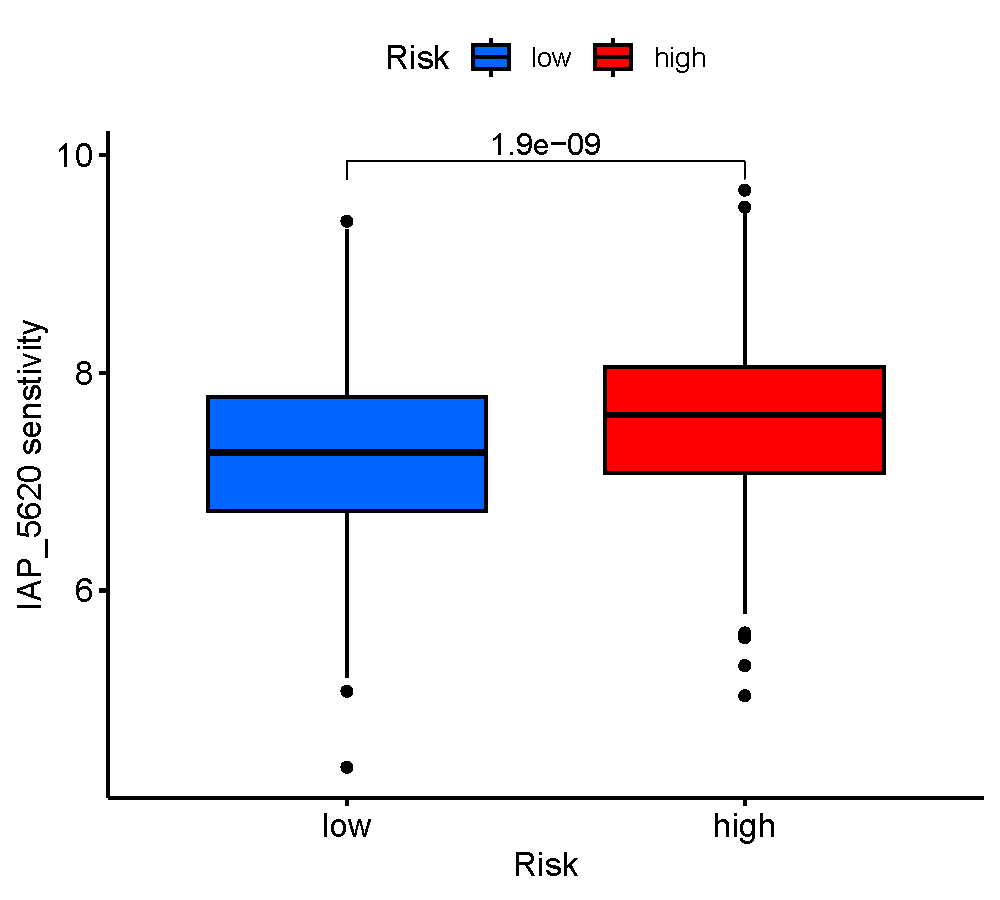

Supplement: Supplementary Figure S1 — Unsupervised clustering of disulfidptosis-related genes and Consensus matrix heatmaps for k = 3-9. [file DataSheet1.zip › Fiugre S5/drugSenstivity.IAP_5620.png]

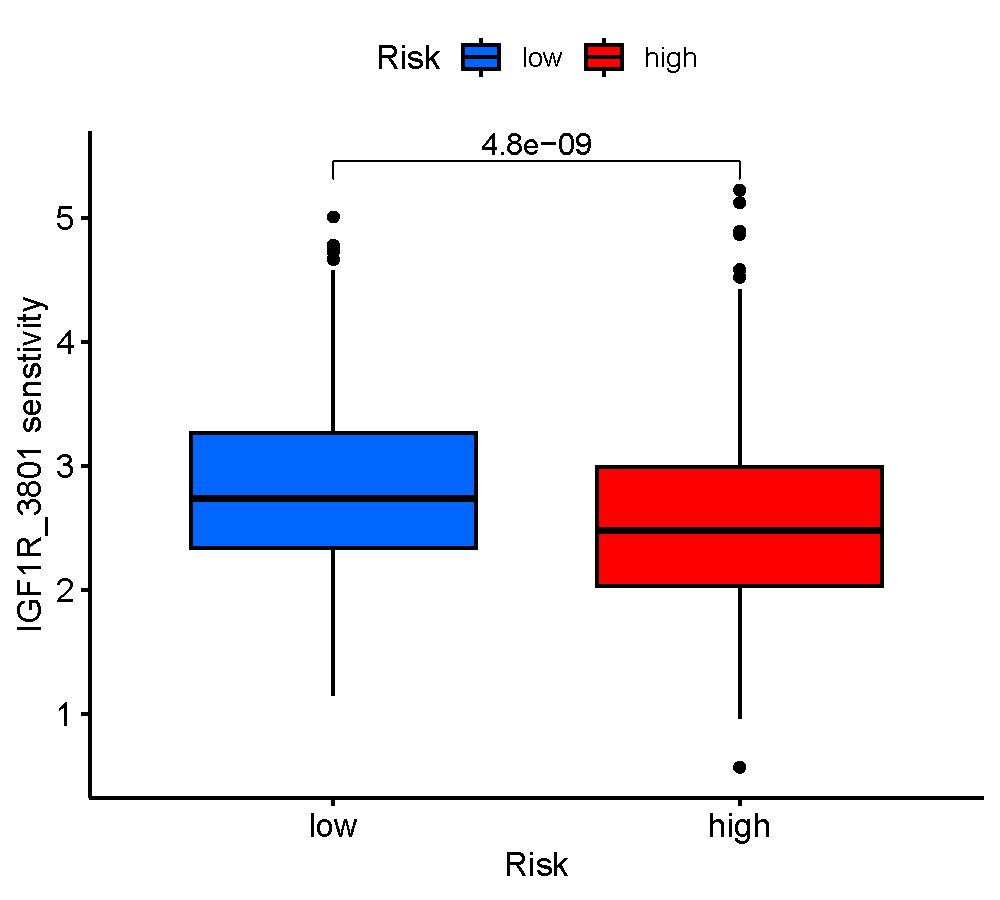

Supplement: Supplementary Figure S1 — Unsupervised clustering of disulfidptosis-related genes and Consensus matrix heatmaps for k = 3-9. [file DataSheet1.zip › Fiugre S5/drugSenstivity.IGF1R_3801.png]

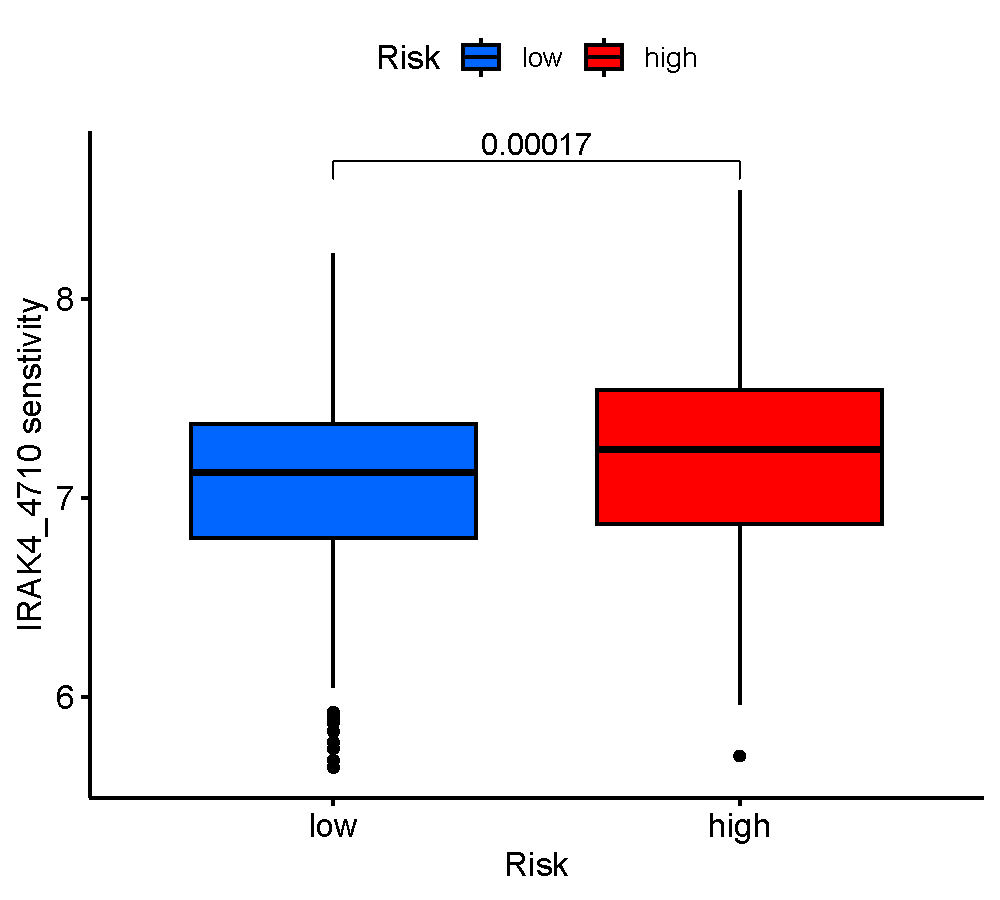

Supplement: Supplementary Figure S1 — Unsupervised clustering of disulfidptosis-related genes and Consensus matrix heatmaps for k = 3-9. [file DataSheet1.zip › Fiugre S5/drugSenstivity.IRAK4_4710.png]

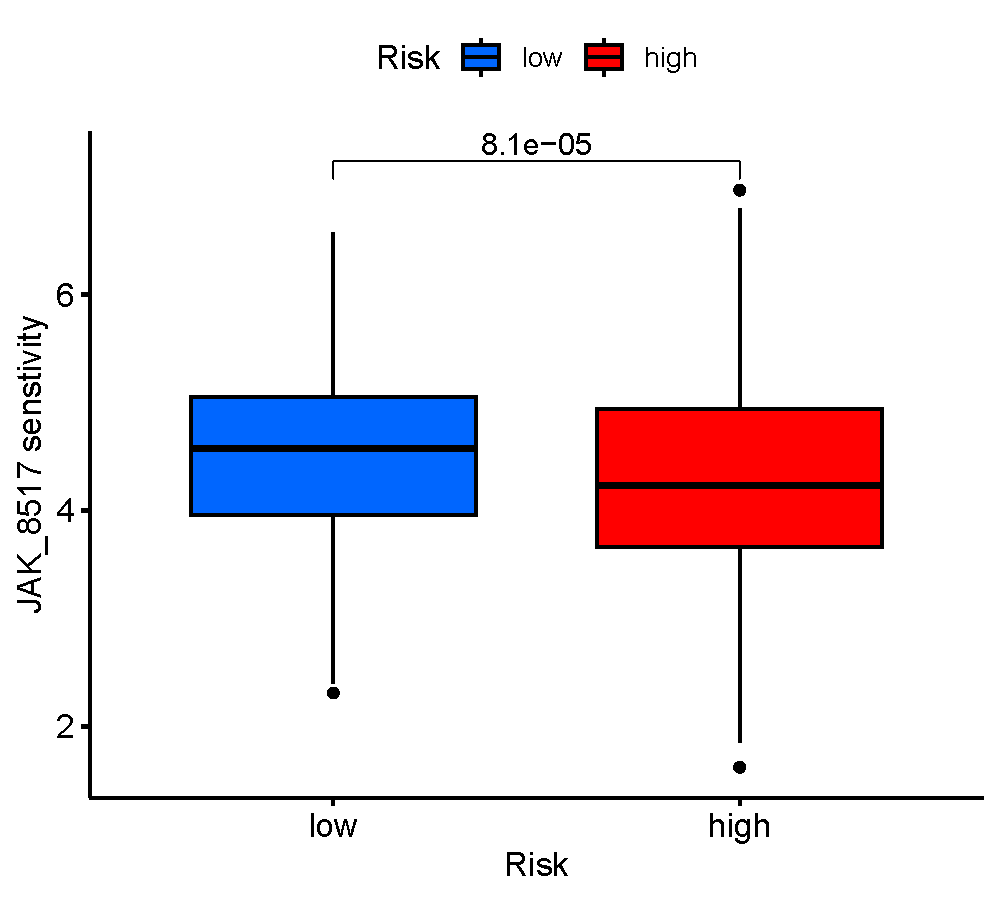

Supplement: Supplementary Figure S1 — Unsupervised clustering of disulfidptosis-related genes and Consensus matrix heatmaps for k = 3-9. [file DataSheet1.zip › Fiugre S5/drugSenstivity.JAK_8517.png]

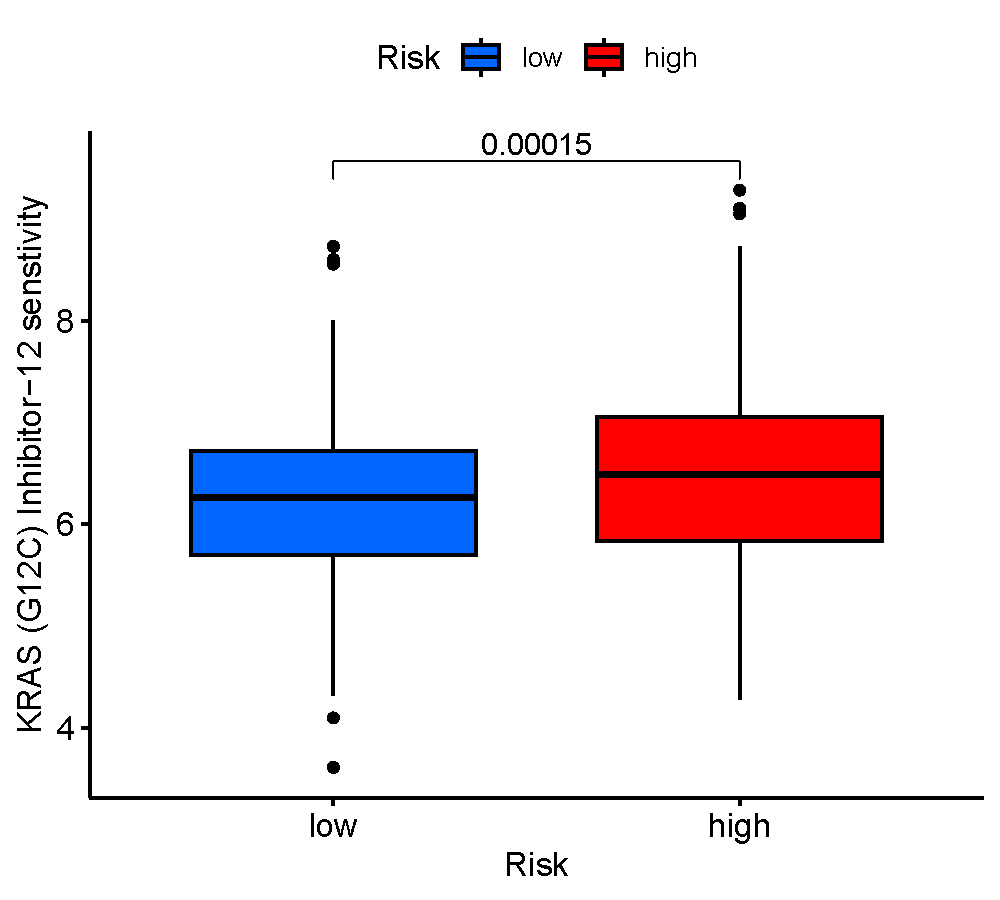

Supplement: Supplementary Figure S1 — Unsupervised clustering of disulfidptosis-related genes and Consensus matrix heatmaps for k = 3-9. [file DataSheet1.zip › Fiugre S5/drugSenstivity.KRAS (G12C) Inhibitor-12.png]

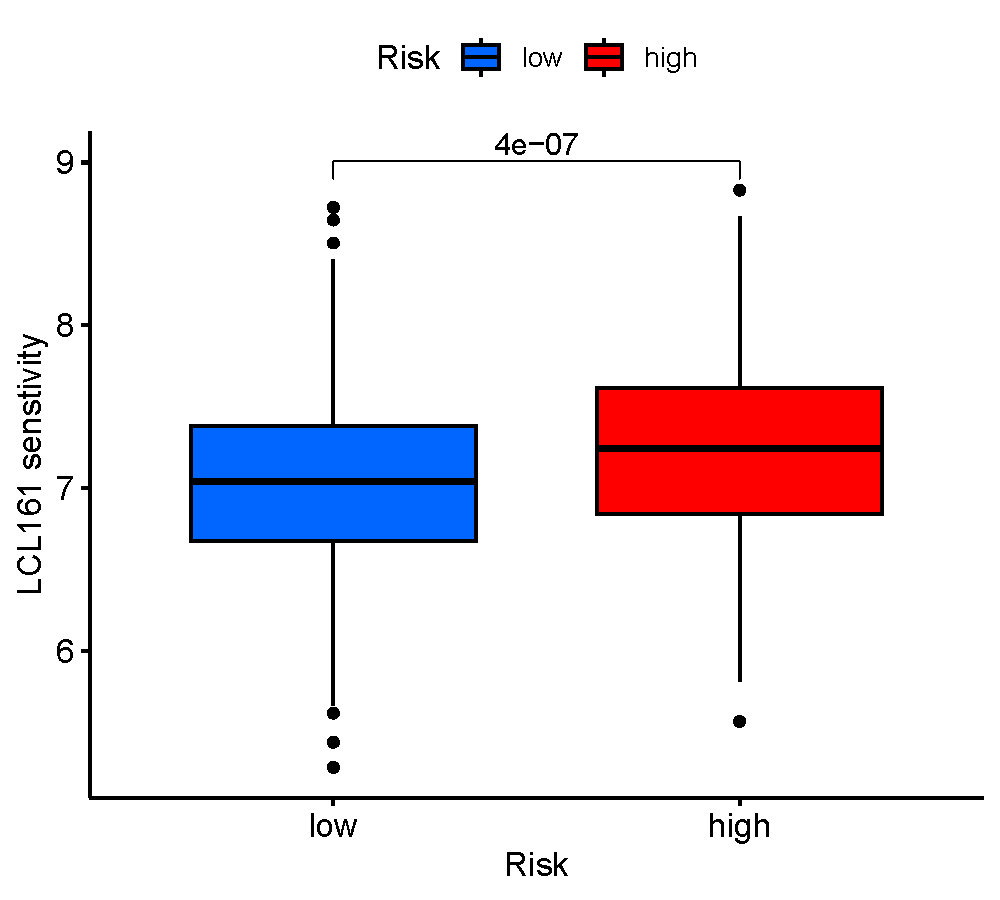

Supplement: Supplementary Figure S1 — Unsupervised clustering of disulfidptosis-related genes and Consensus matrix heatmaps for k = 3-9. [file DataSheet1.zip › Fiugre S5/drugSenstivity.LCL161.png]

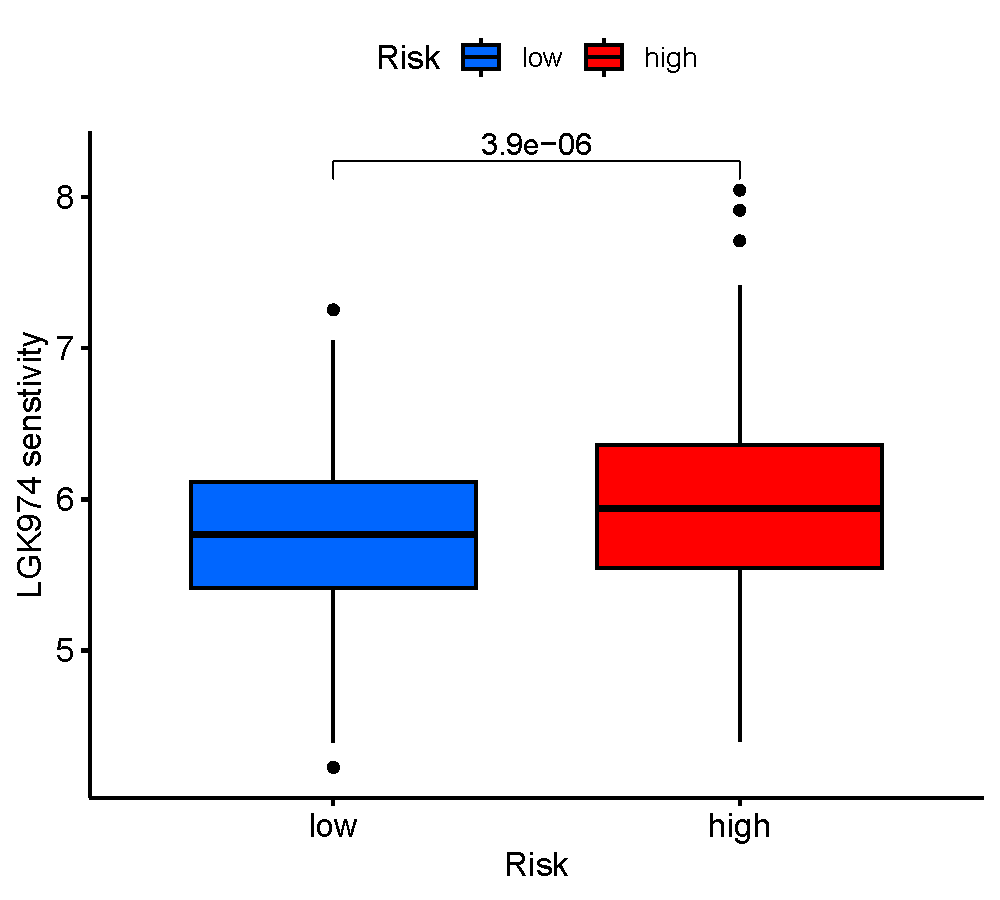

Supplement: Supplementary Figure S1 — Unsupervised clustering of disulfidptosis-related genes and Consensus matrix heatmaps for k = 3-9. [file DataSheet1.zip › Fiugre S5/drugSenstivity.LGK974.png]

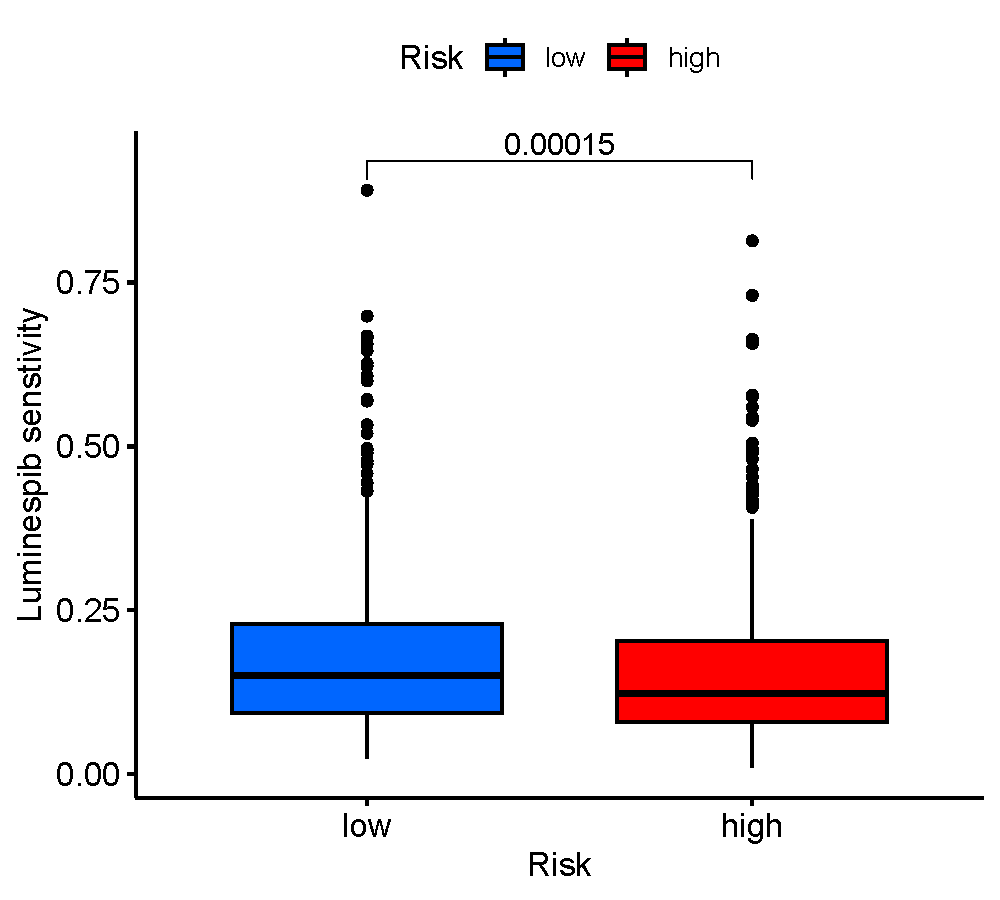

Supplement: Supplementary Figure S1 — Unsupervised clustering of disulfidptosis-related genes and Consensus matrix heatmaps for k = 3-9. [file DataSheet1.zip › Fiugre S5/drugSenstivity.Luminespib.png]

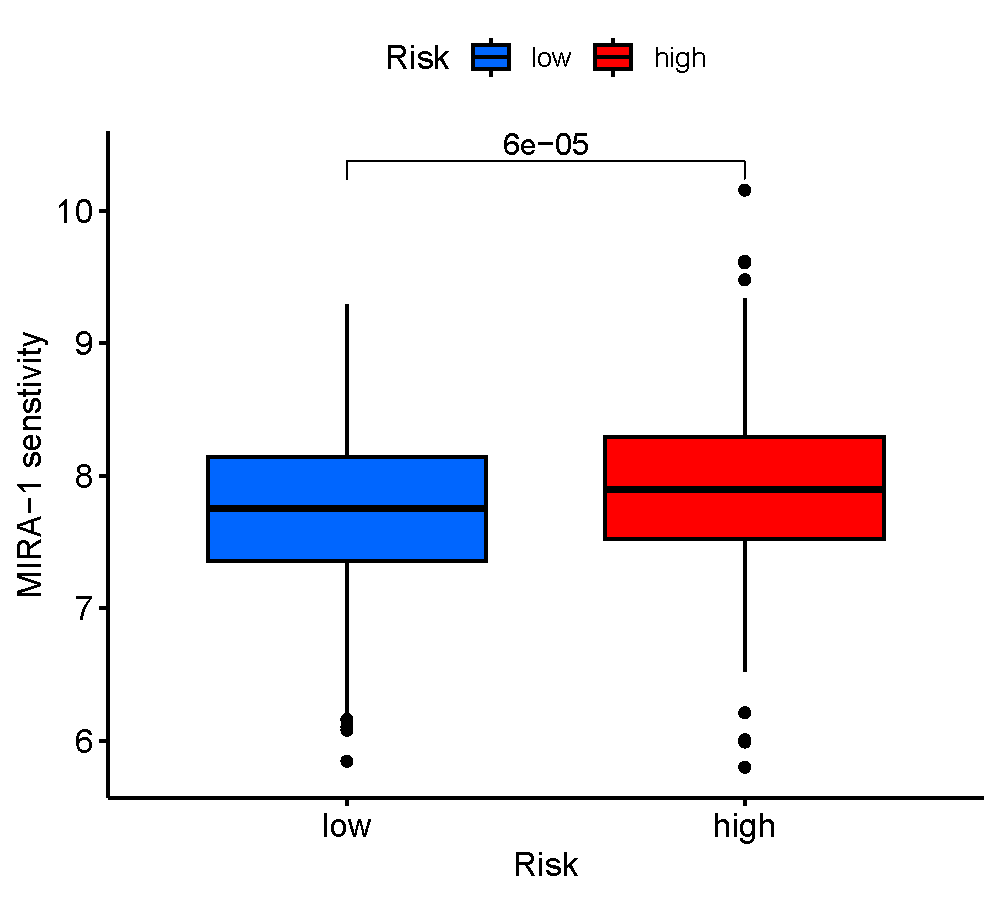

Supplement: Supplementary Figure S1 — Unsupervised clustering of disulfidptosis-related genes and Consensus matrix heatmaps for k = 3-9. [file DataSheet1.zip › Fiugre S5/drugSenstivity.MIRA-1.png]

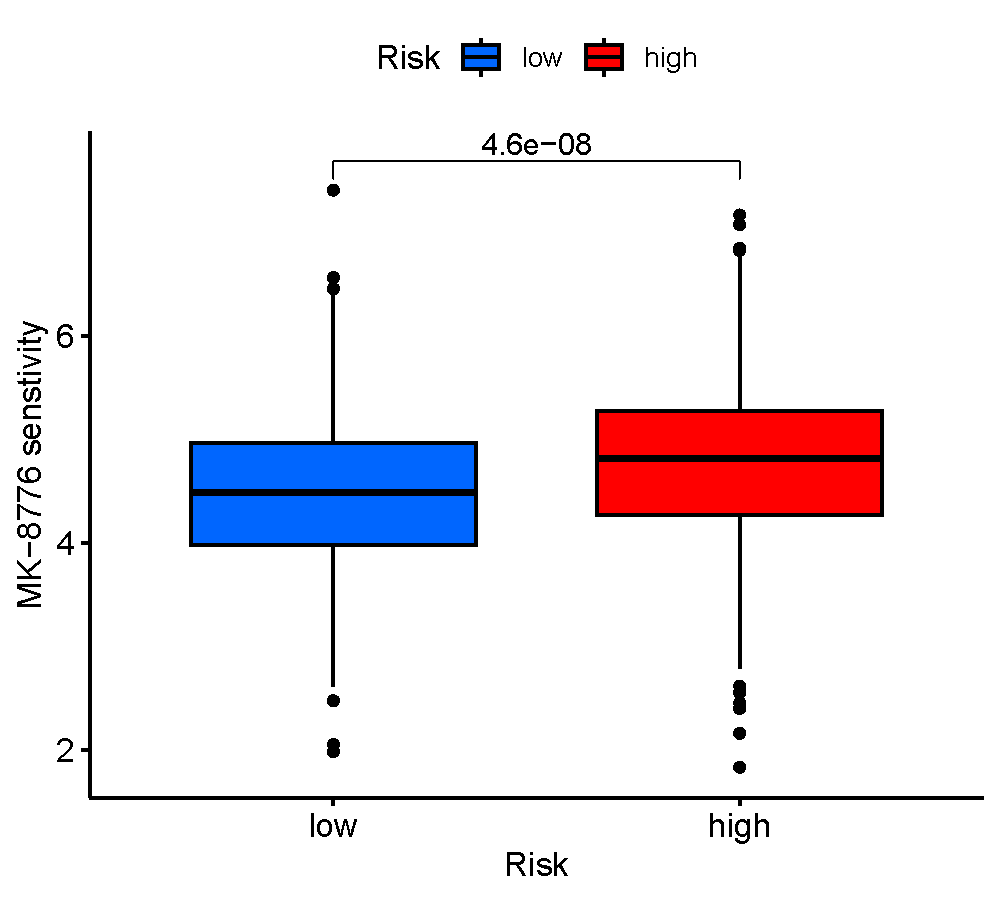

Supplement: Supplementary Figure S1 — Unsupervised clustering of disulfidptosis-related genes and Consensus matrix heatmaps for k = 3-9. [file DataSheet1.zip › Fiugre S5/drugSenstivity.MK-8776.png]

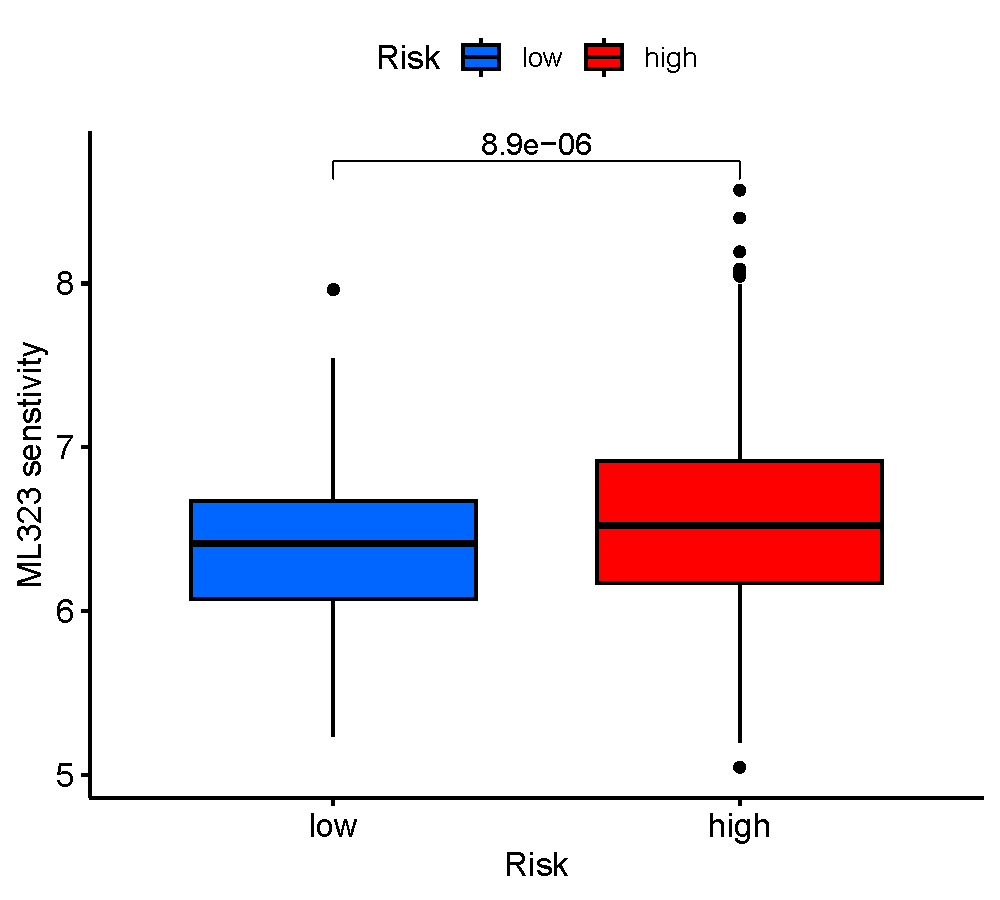

Supplement: Supplementary Figure S1 — Unsupervised clustering of disulfidptosis-related genes and Consensus matrix heatmaps for k = 3-9. [file DataSheet1.zip › Fiugre S5/drugSenstivity.ML323.png]

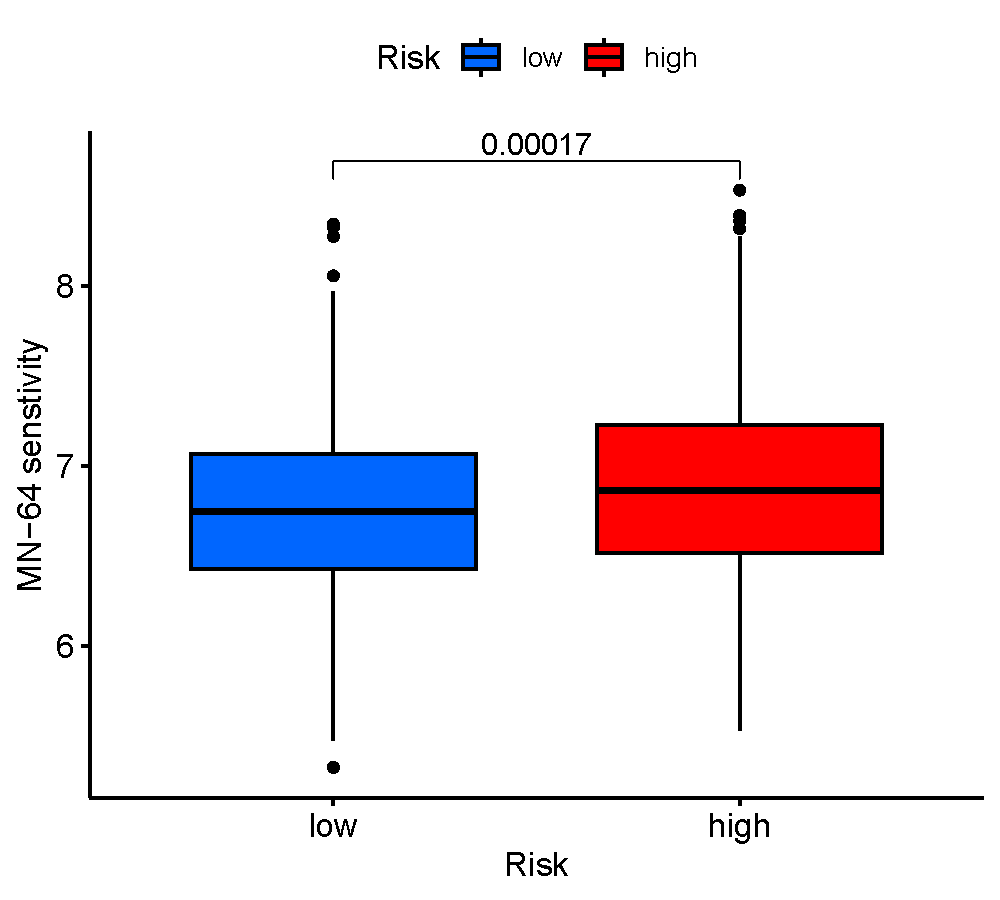

Supplement: Supplementary Figure S1 — Unsupervised clustering of disulfidptosis-related genes and Consensus matrix heatmaps for k = 3-9. [file DataSheet1.zip › Fiugre S5/drugSenstivity.MN-64.png]

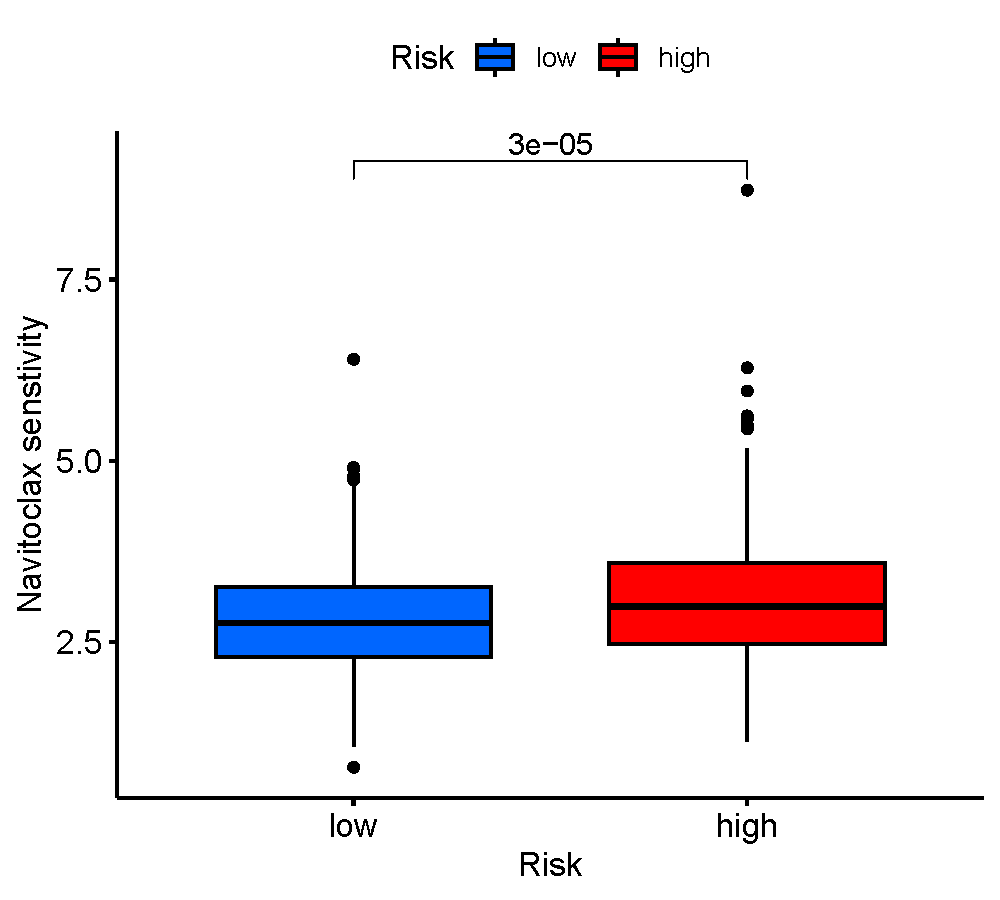

Supplement: Supplementary Figure S1 — Unsupervised clustering of disulfidptosis-related genes and Consensus matrix heatmaps for k = 3-9. [file DataSheet1.zip › Fiugre S5/drugSenstivity.Navitoclax.png]

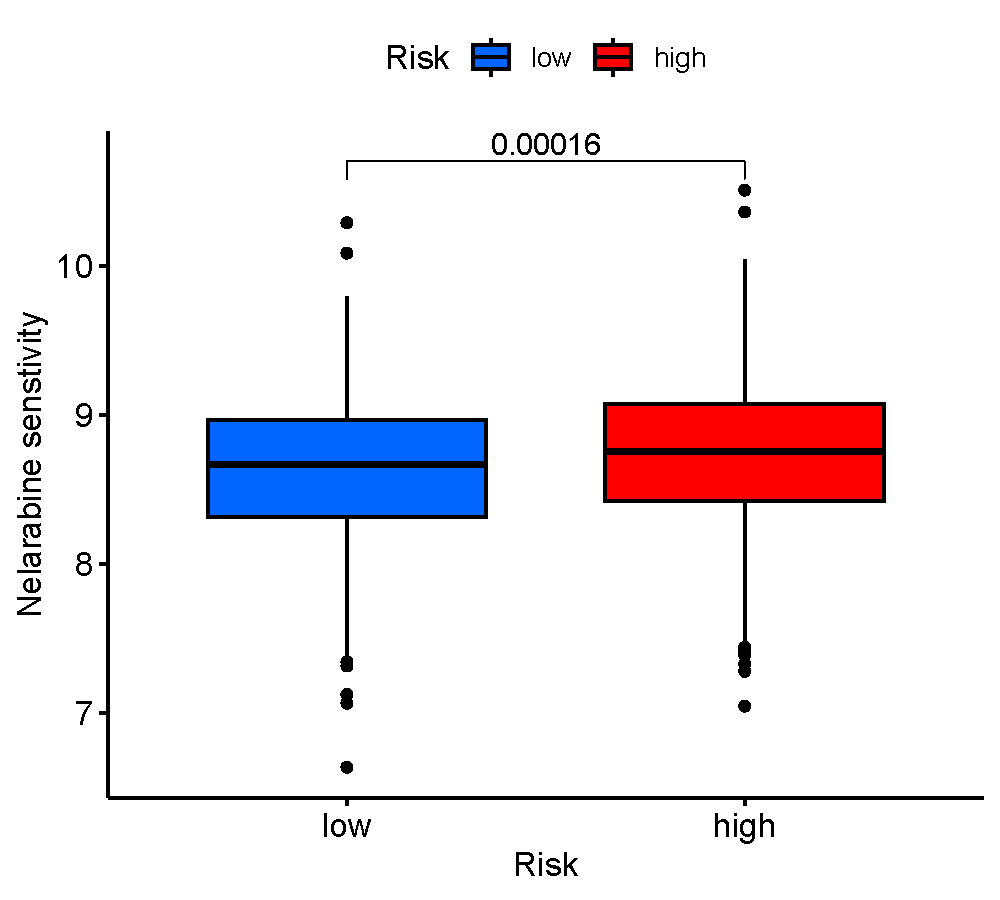

Supplement: Supplementary Figure S1 — Unsupervised clustering of disulfidptosis-related genes and Consensus matrix heatmaps for k = 3-9. [file DataSheet1.zip › Fiugre S5/drugSenstivity.Nelarabine.png]

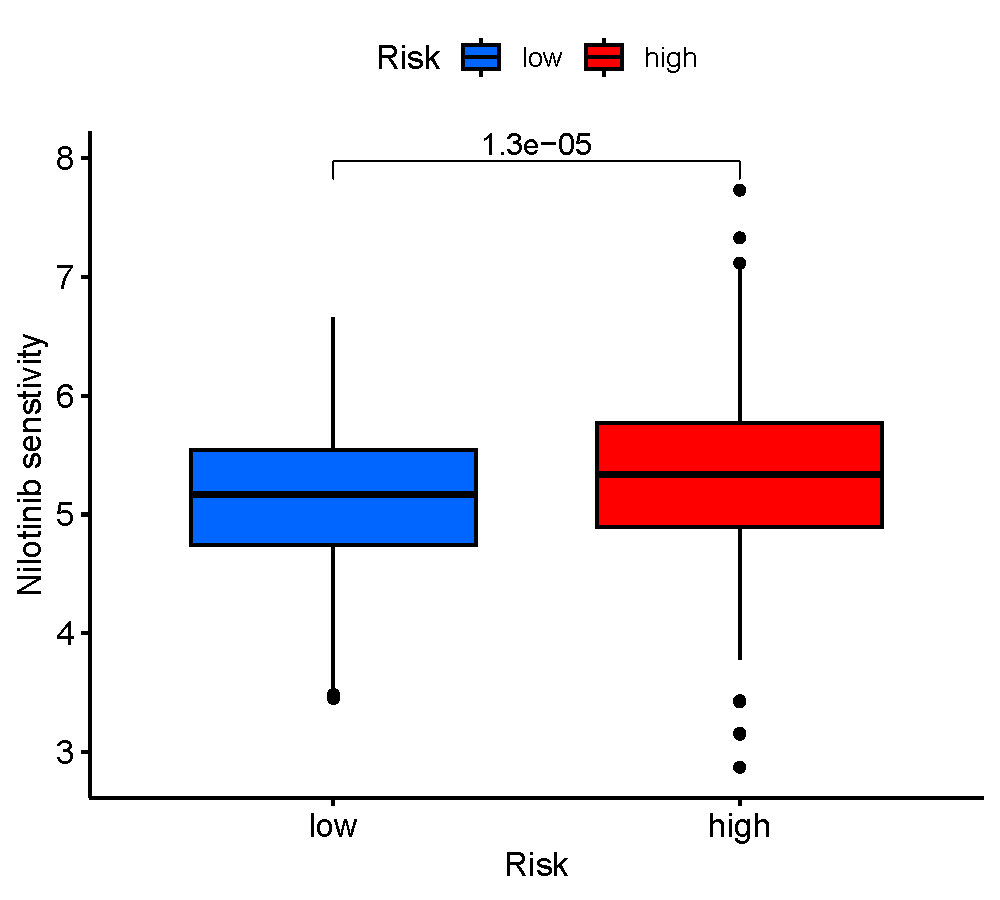

Supplement: Supplementary Figure S1 — Unsupervised clustering of disulfidptosis-related genes and Consensus matrix heatmaps for k = 3-9. [file DataSheet1.zip › Fiugre S5/drugSenstivity.Nilotinib.png]

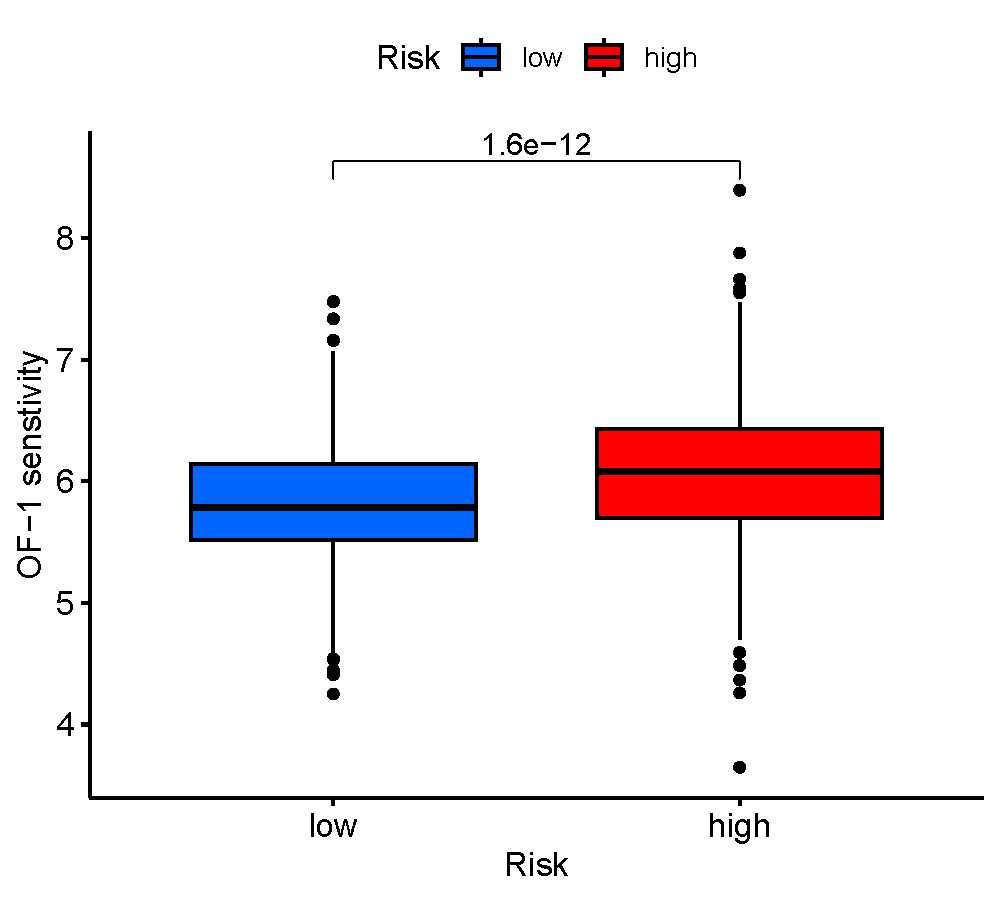

Supplement: Supplementary Figure S1 — Unsupervised clustering of disulfidptosis-related genes and Consensus matrix heatmaps for k = 3-9. [file DataSheet1.zip › Fiugre S5/drugSenstivity.OF-1.png]

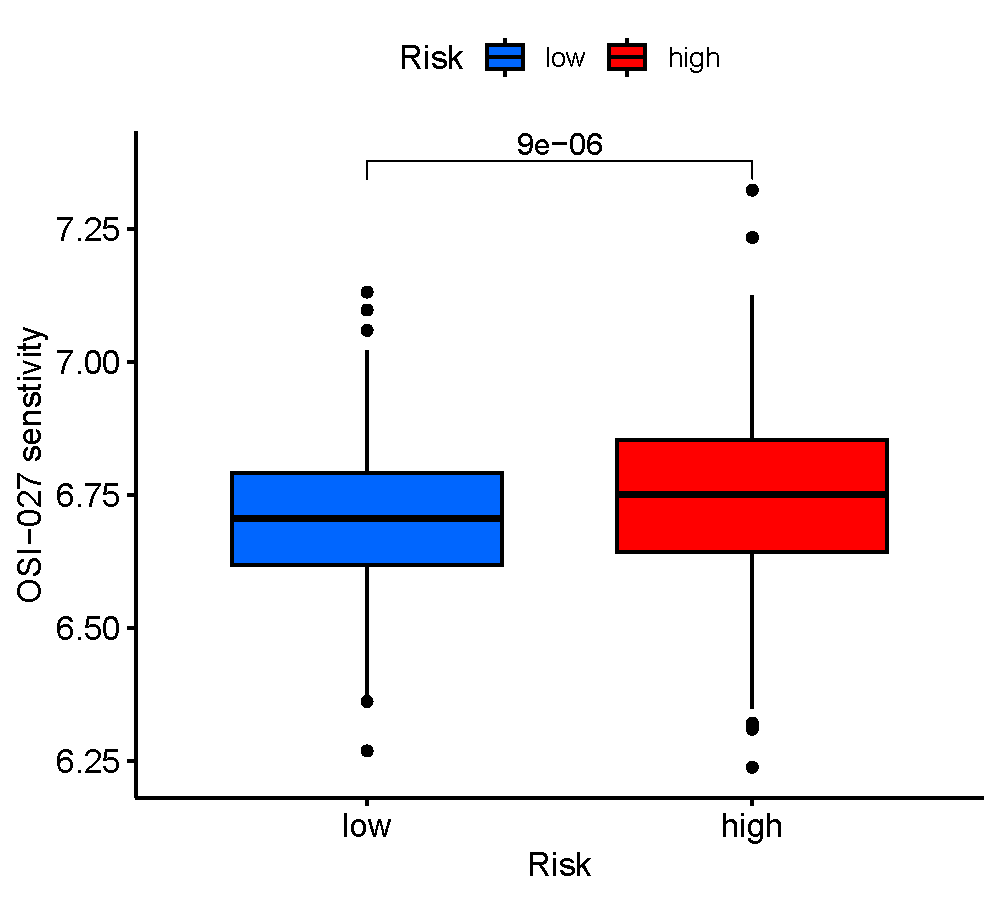

Supplement: Supplementary Figure S1 — Unsupervised clustering of disulfidptosis-related genes and Consensus matrix heatmaps for k = 3-9. [file DataSheet1.zip › Fiugre S5/drugSenstivity.OSI-027.png]

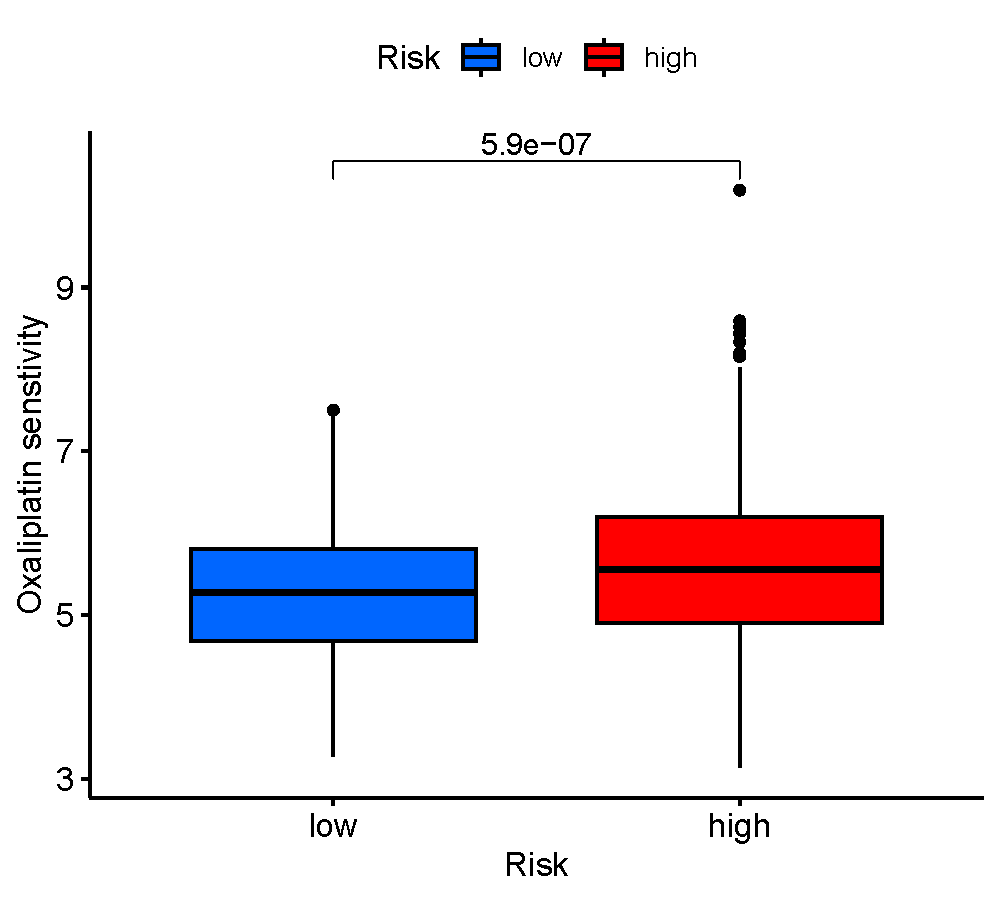

Supplement: Supplementary Figure S1 — Unsupervised clustering of disulfidptosis-related genes and Consensus matrix heatmaps for k = 3-9. [file DataSheet1.zip › Fiugre S5/drugSenstivity.Oxaliplatin.png]

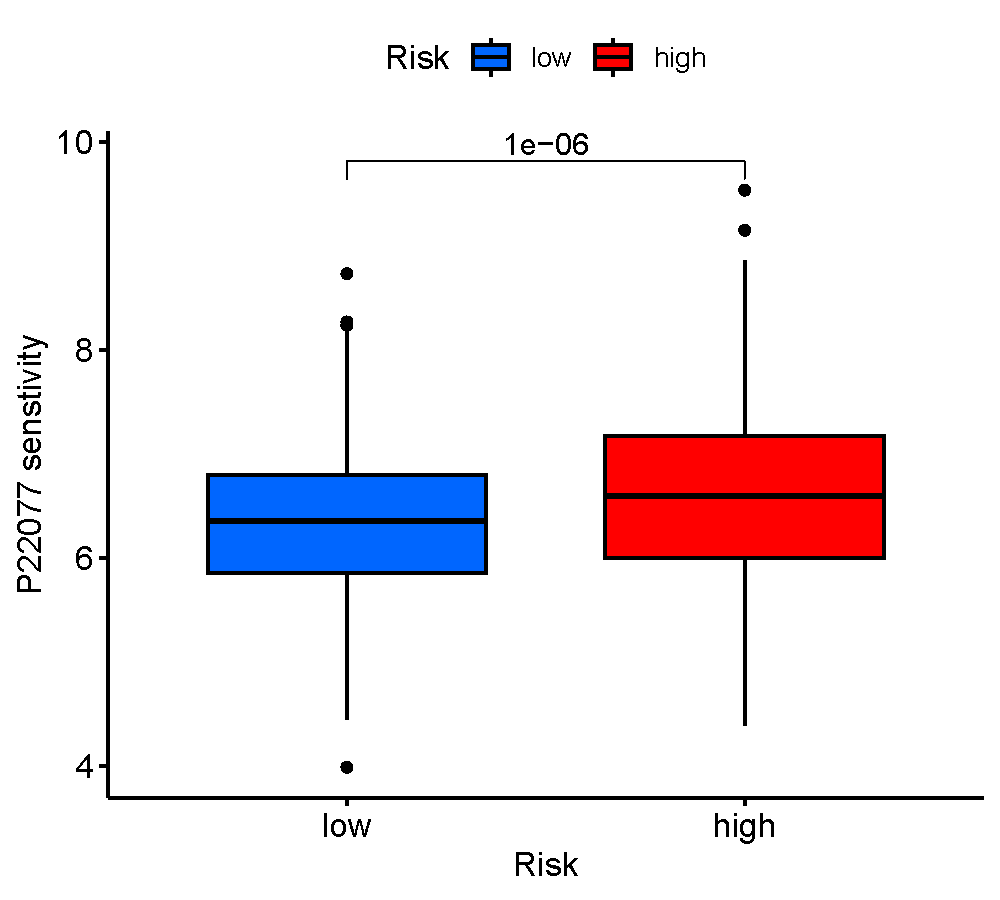

Supplement: Supplementary Figure S1 — Unsupervised clustering of disulfidptosis-related genes and Consensus matrix heatmaps for k = 3-9. [file DataSheet1.zip › Fiugre S5/drugSenstivity.P22077.png]

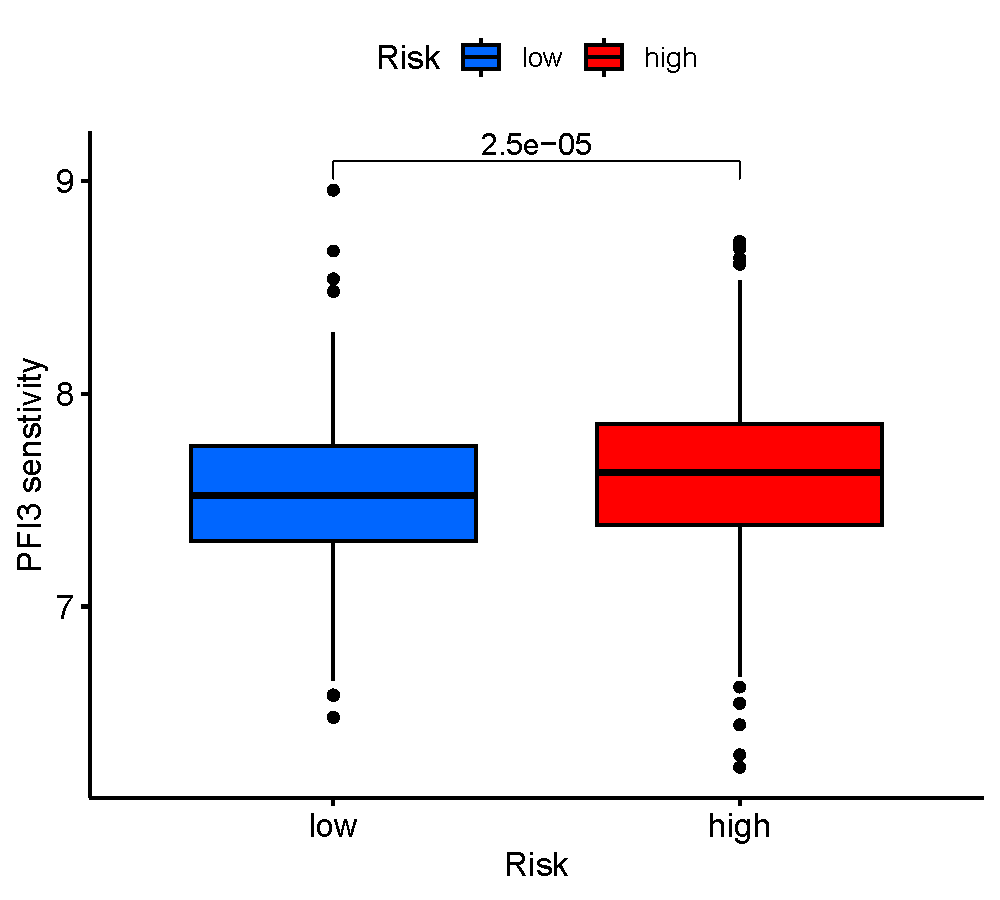

Supplement: Supplementary Figure S1 — Unsupervised clustering of disulfidptosis-related genes and Consensus matrix heatmaps for k = 3-9. [file DataSheet1.zip › Fiugre S5/drugSenstivity.PFI3.png]

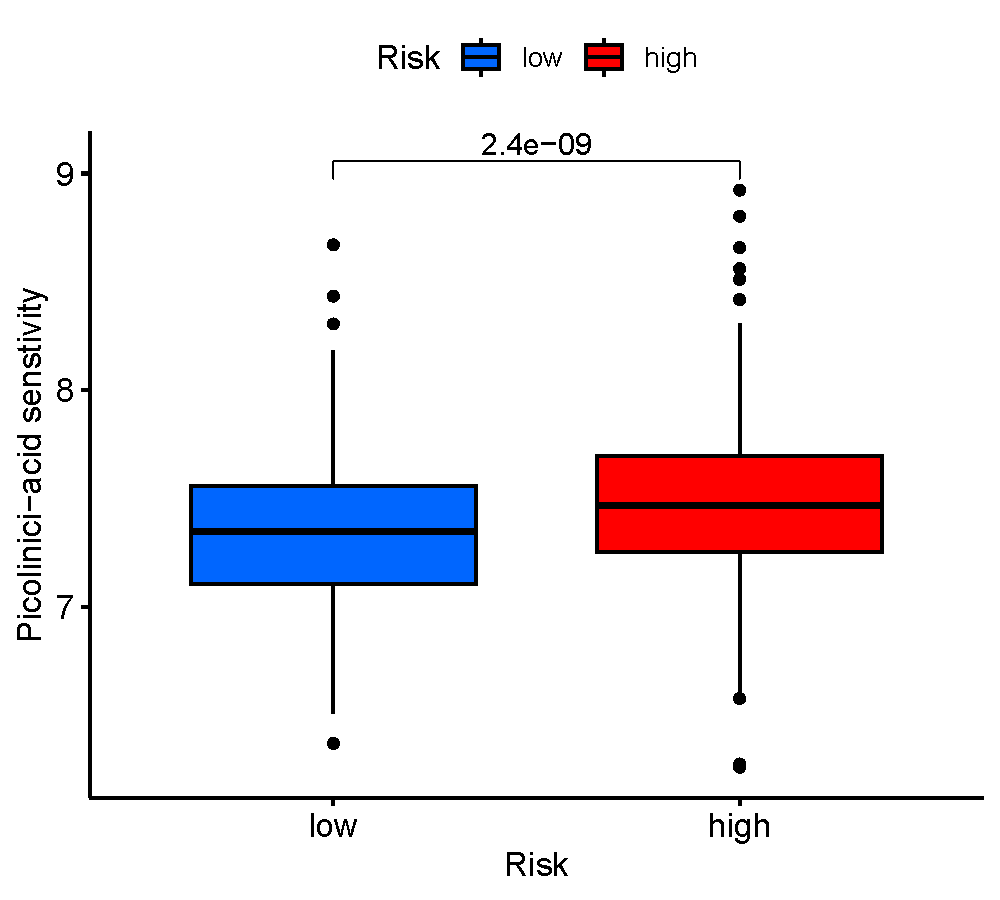

Supplement: Supplementary Figure S1 — Unsupervised clustering of disulfidptosis-related genes and Consensus matrix heatmaps for k = 3-9. [file DataSheet1.zip › Fiugre S5/drugSenstivity.Picolinici-acid.png]

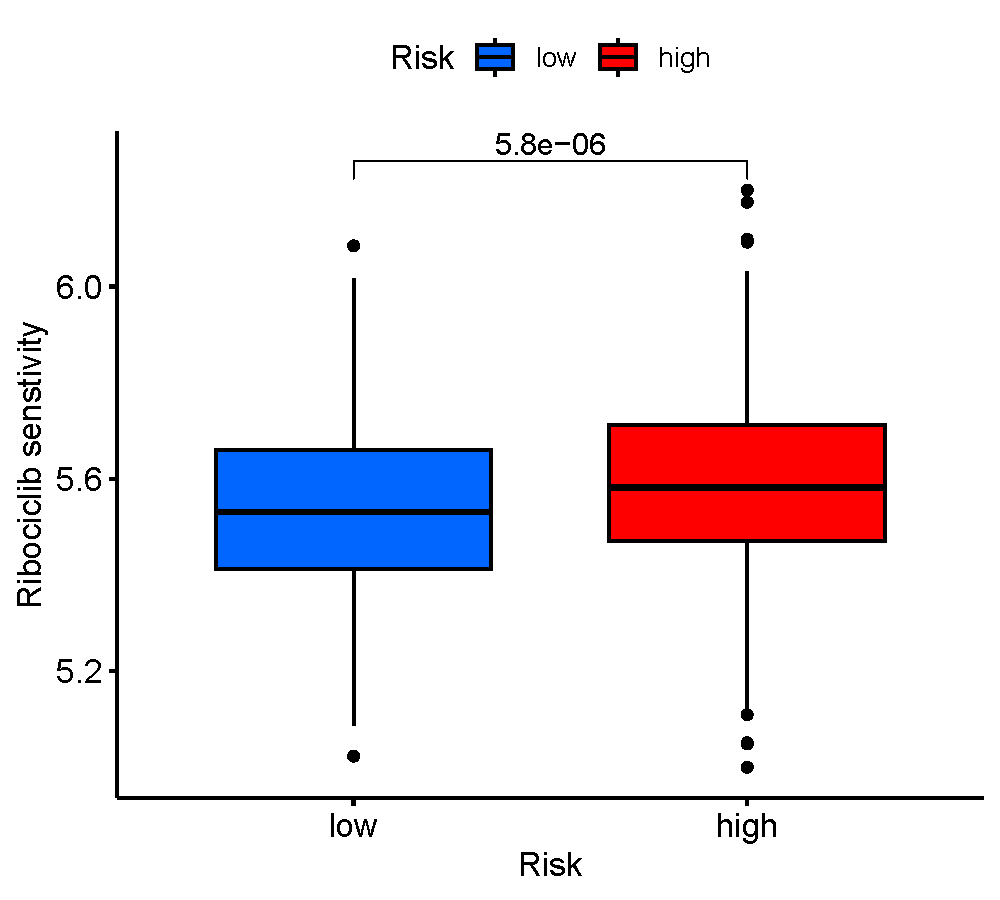

Supplement: Supplementary Figure S1 — Unsupervised clustering of disulfidptosis-related genes and Consensus matrix heatmaps for k = 3-9. [file DataSheet1.zip › Fiugre S5/drugSenstivity.Ribociclib.png]

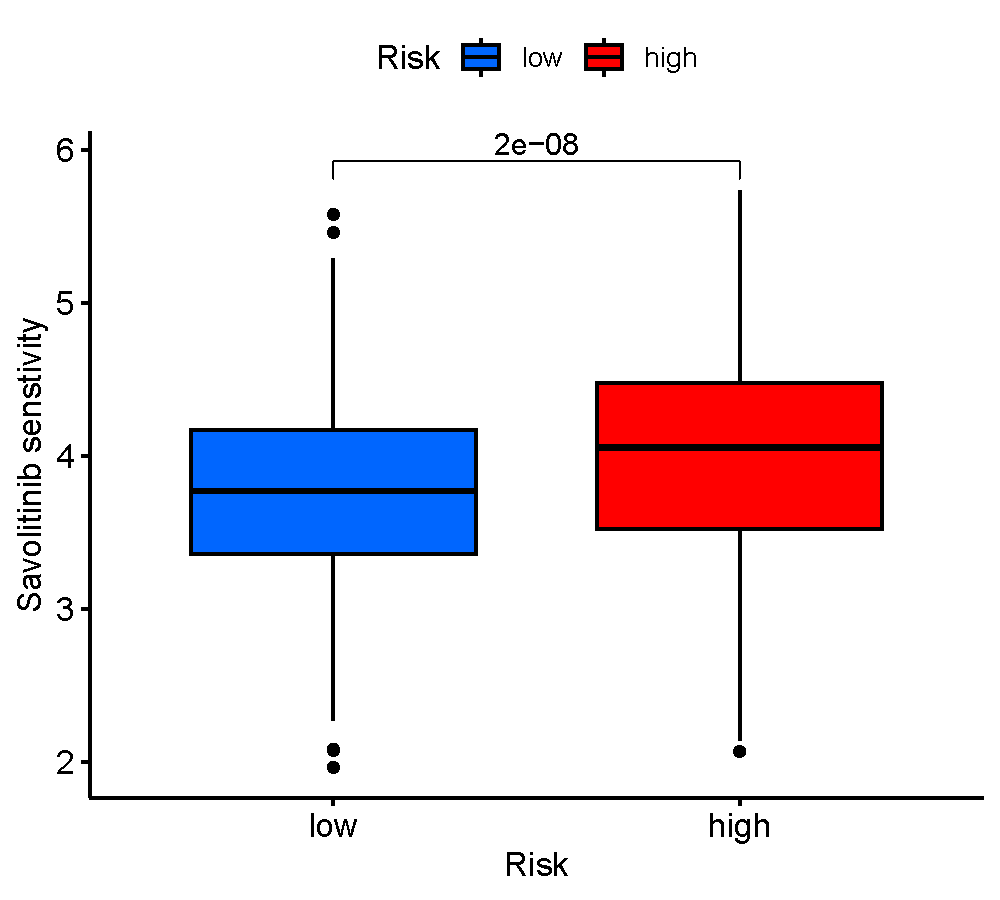

Supplement: Supplementary Figure S1 — Unsupervised clustering of disulfidptosis-related genes and Consensus matrix heatmaps for k = 3-9. [file DataSheet1.zip › Fiugre S5/drugSenstivity.Savolitinib.png]

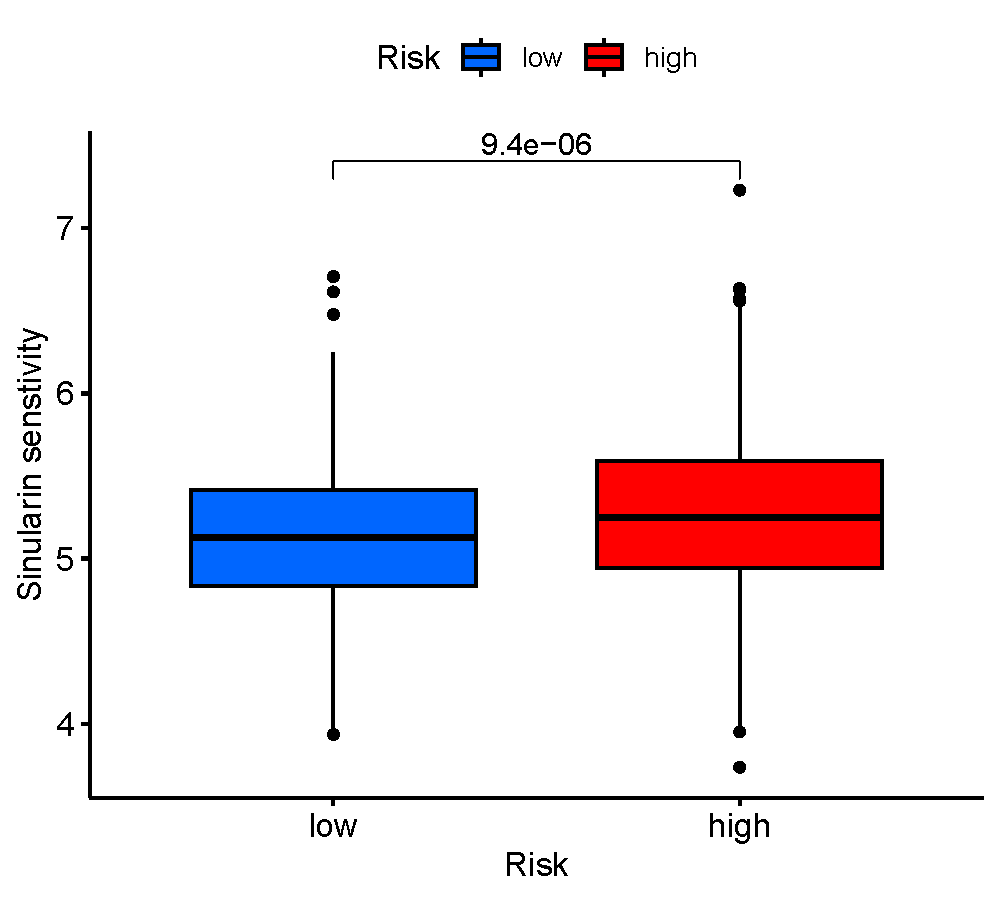

Supplement: Supplementary Figure S1 — Unsupervised clustering of disulfidptosis-related genes and Consensus matrix heatmaps for k = 3-9. [file DataSheet1.zip › Fiugre S5/drugSenstivity.Sinularin.png]

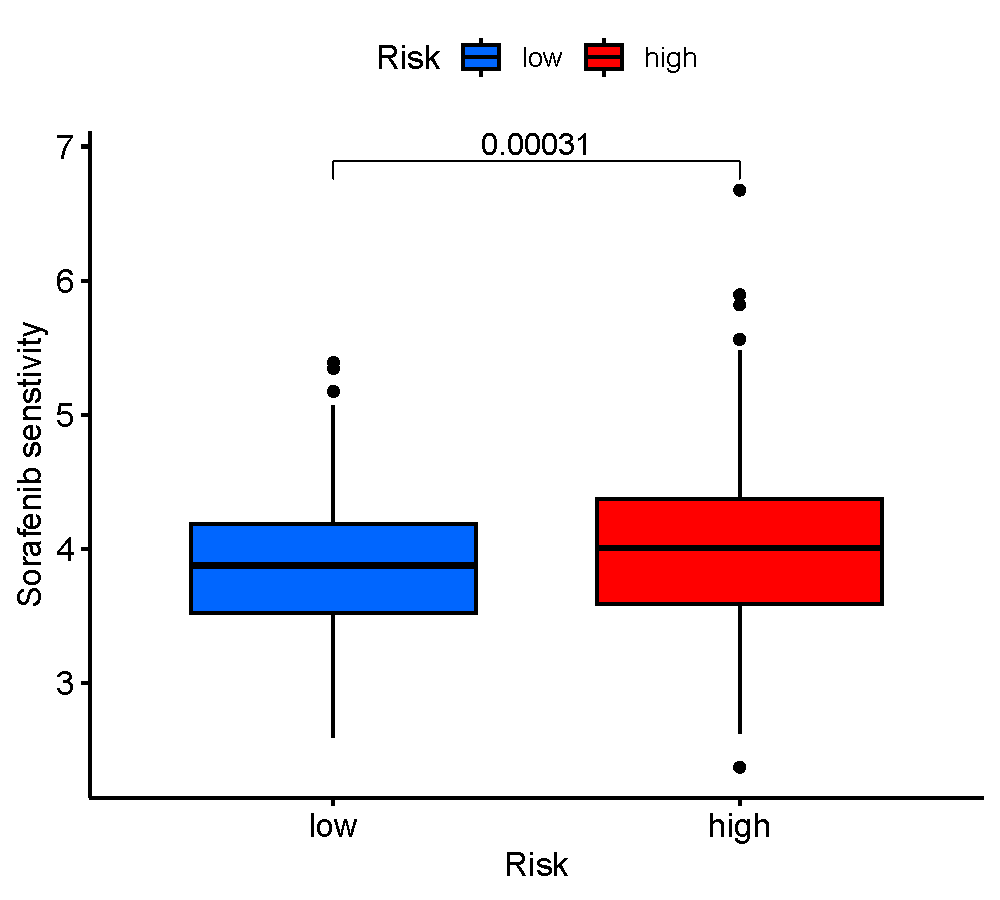

Supplement: Supplementary Figure S1 — Unsupervised clustering of disulfidptosis-related genes and Consensus matrix heatmaps for k = 3-9. [file DataSheet1.zip › Fiugre S5/drugSenstivity.Sorafenib.png]

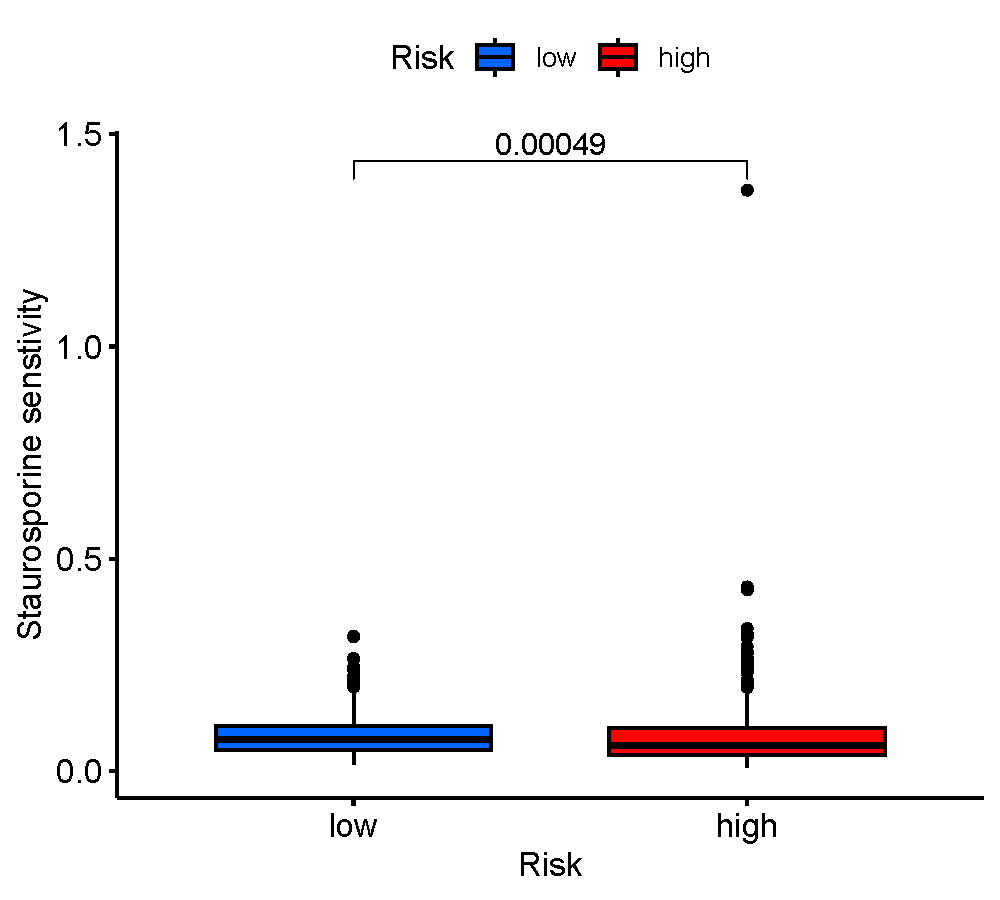

Supplement: Supplementary Figure S1 — Unsupervised clustering of disulfidptosis-related genes and Consensus matrix heatmaps for k = 3-9. [file DataSheet1.zip › Fiugre S5/drugSenstivity.Staurosporine.png]

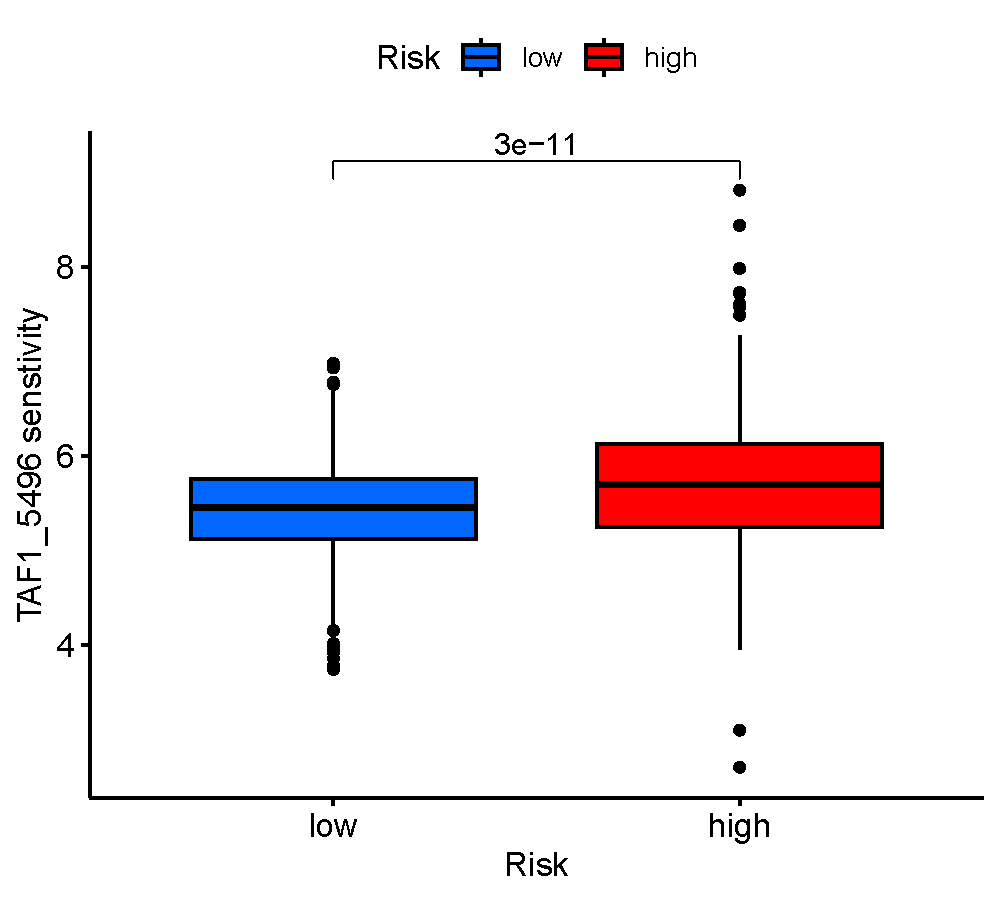

Supplement: Supplementary Figure S1 — Unsupervised clustering of disulfidptosis-related genes and Consensus matrix heatmaps for k = 3-9. [file DataSheet1.zip › Fiugre S5/drugSenstivity.TAF1_5496.png]

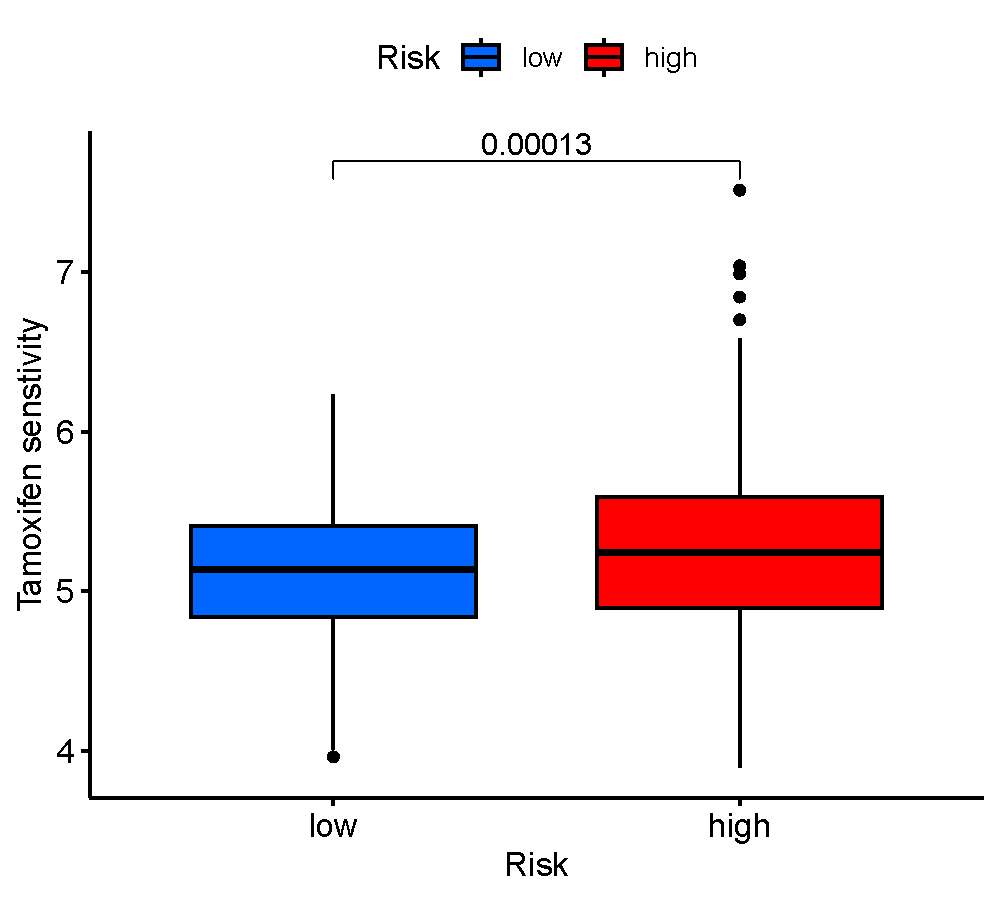

Supplement: Supplementary Figure S1 — Unsupervised clustering of disulfidptosis-related genes and Consensus matrix heatmaps for k = 3-9. [file DataSheet1.zip › Fiugre S5/drugSenstivity.Tamoxifen.png]

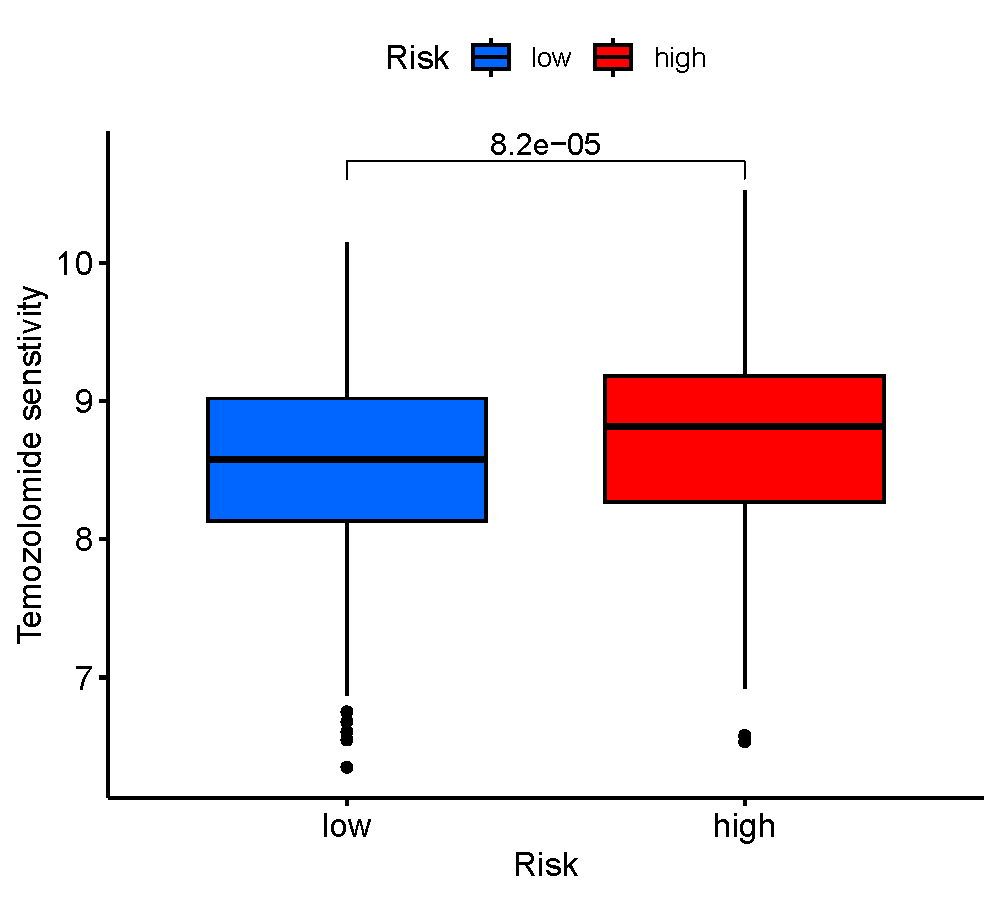

Supplement: Supplementary Figure S1 — Unsupervised clustering of disulfidptosis-related genes and Consensus matrix heatmaps for k = 3-9. [file DataSheet1.zip › Fiugre S5/drugSenstivity.Temozolomide.png]

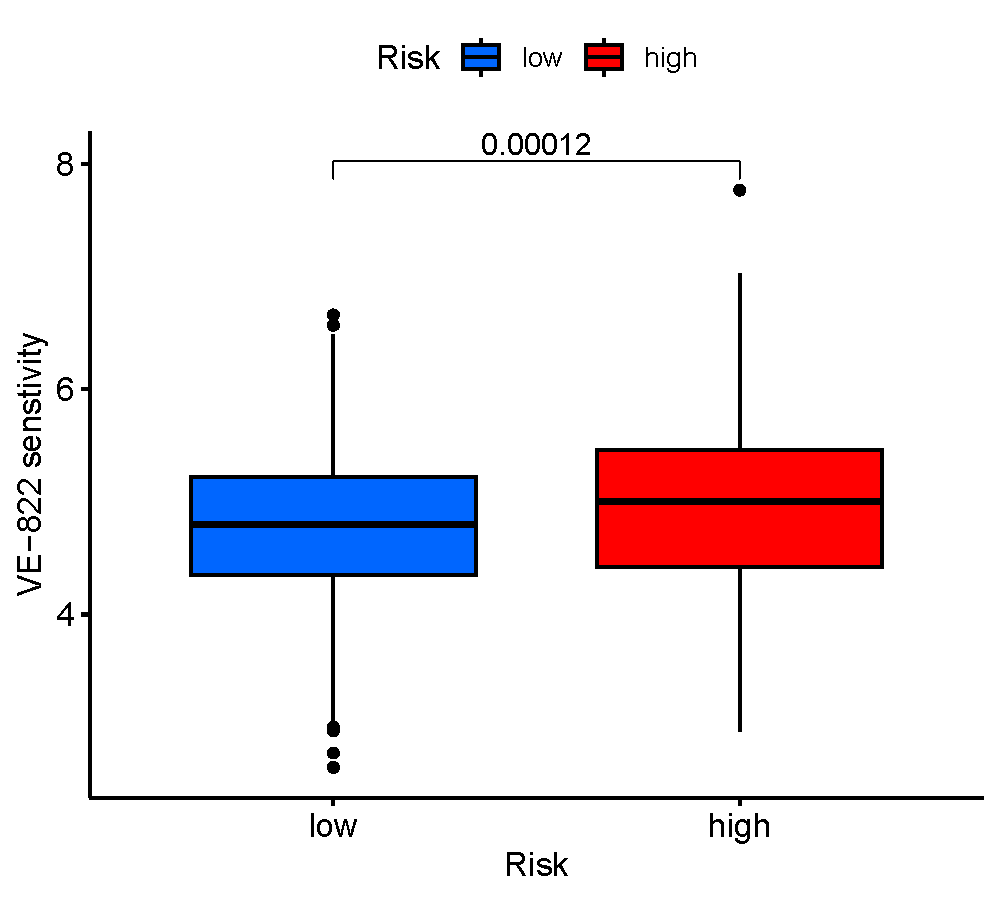

Supplement: Supplementary Figure S1 — Unsupervised clustering of disulfidptosis-related genes and Consensus matrix heatmaps for k = 3-9. [file DataSheet1.zip › Fiugre S5/drugSenstivity.VE-822.png]

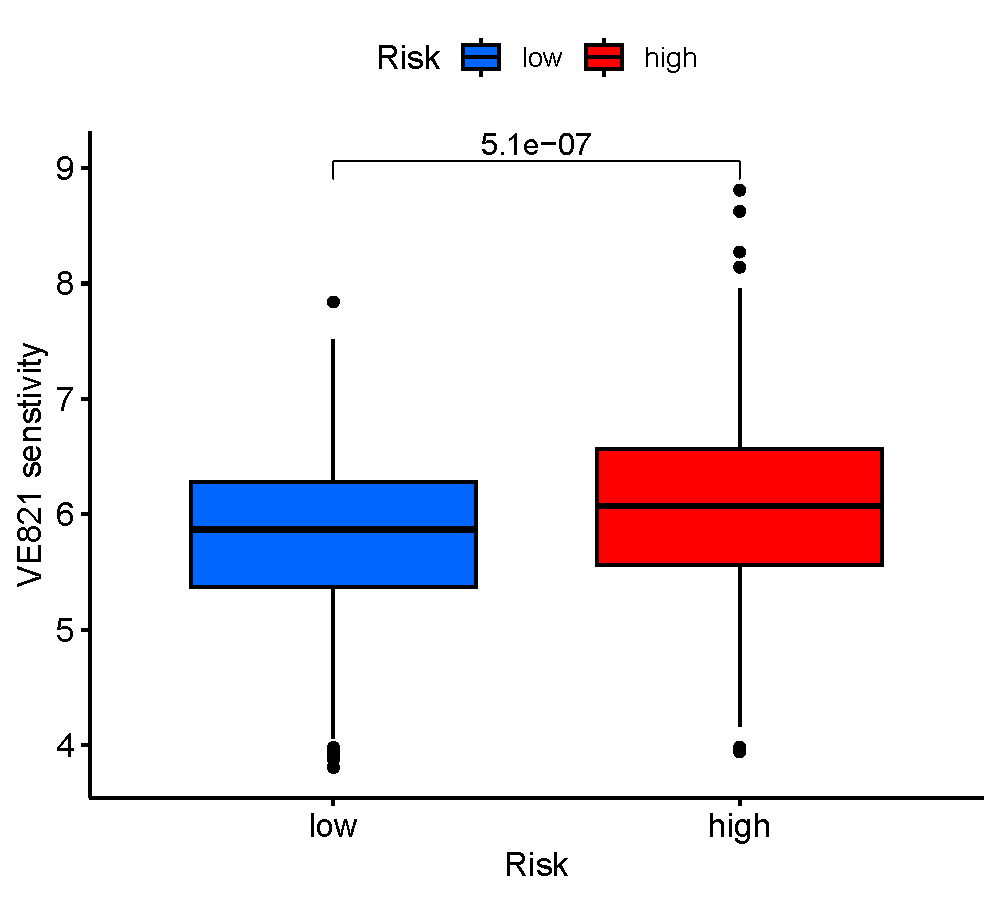

Supplement: Supplementary Figure S1 — Unsupervised clustering of disulfidptosis-related genes and Consensus matrix heatmaps for k = 3-9. [file DataSheet1.zip › Fiugre S5/drugSenstivity.VE821.png]

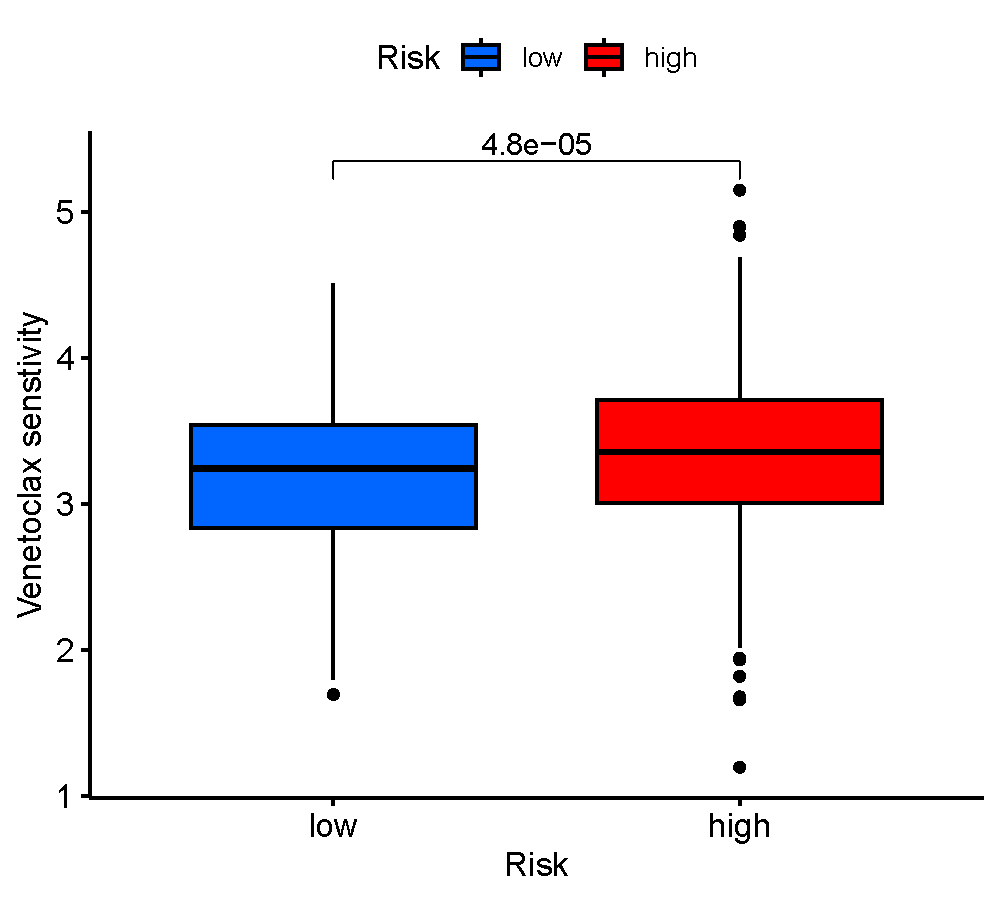

Supplement: Supplementary Figure S1 — Unsupervised clustering of disulfidptosis-related genes and Consensus matrix heatmaps for k = 3-9. [file DataSheet1.zip › Fiugre S5/drugSenstivity.Venetoclax.png]

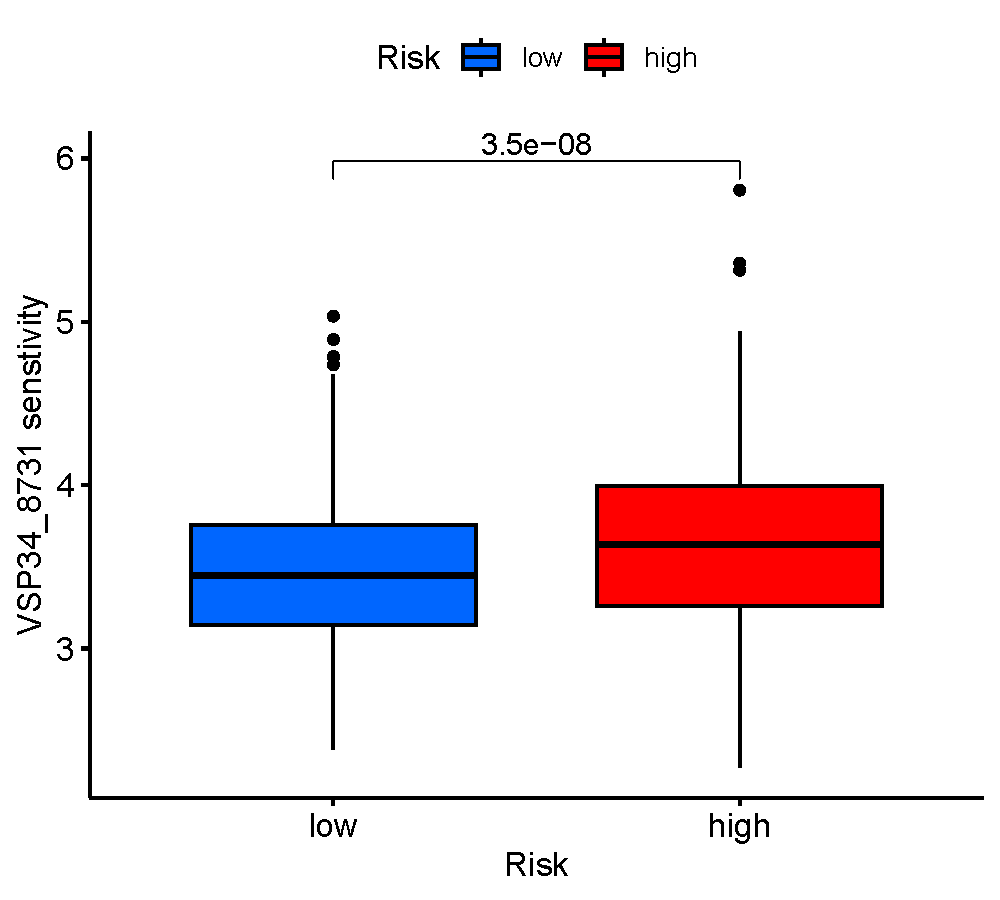

Supplement: Supplementary Figure S1 — Unsupervised clustering of disulfidptosis-related genes and Consensus matrix heatmaps for k = 3-9. [file DataSheet1.zip › Fiugre S5/drugSenstivity.VSP34_8731.png]

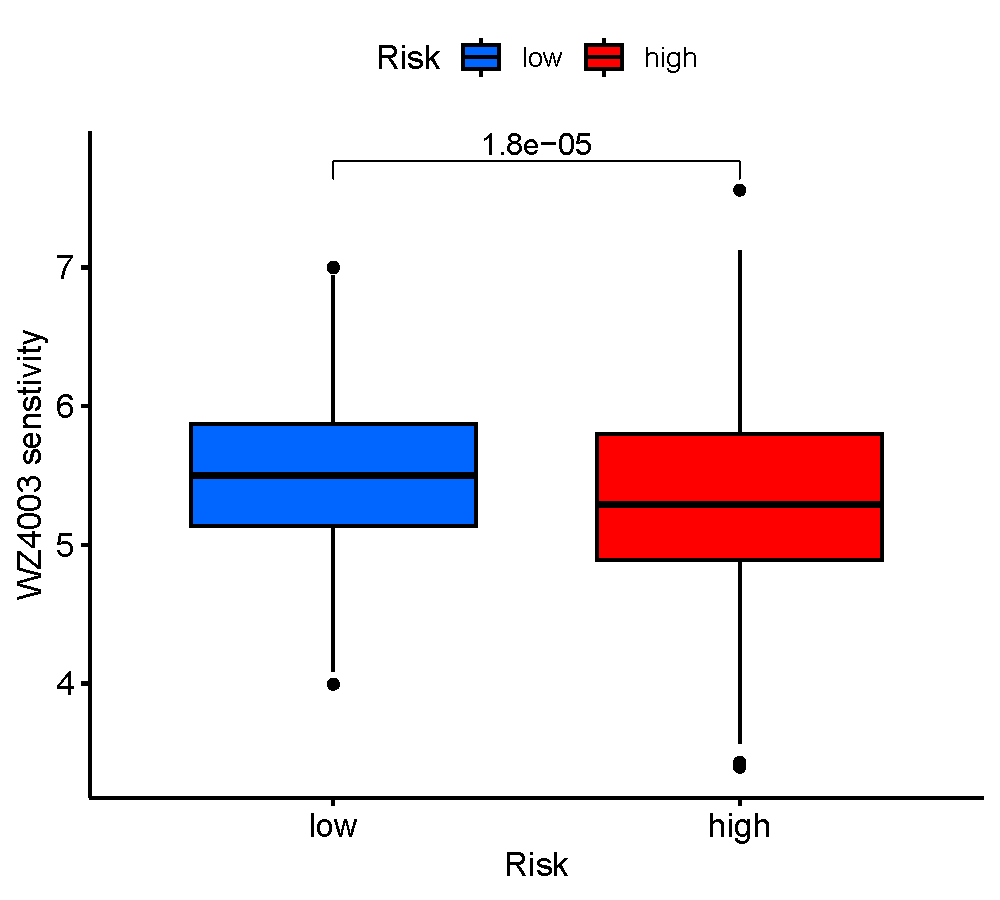

Supplement: Supplementary Figure S1 — Unsupervised clustering of disulfidptosis-related genes and Consensus matrix heatmaps for k = 3-9. [file DataSheet1.zip › Fiugre S5/drugSenstivity.WZ4003.png]

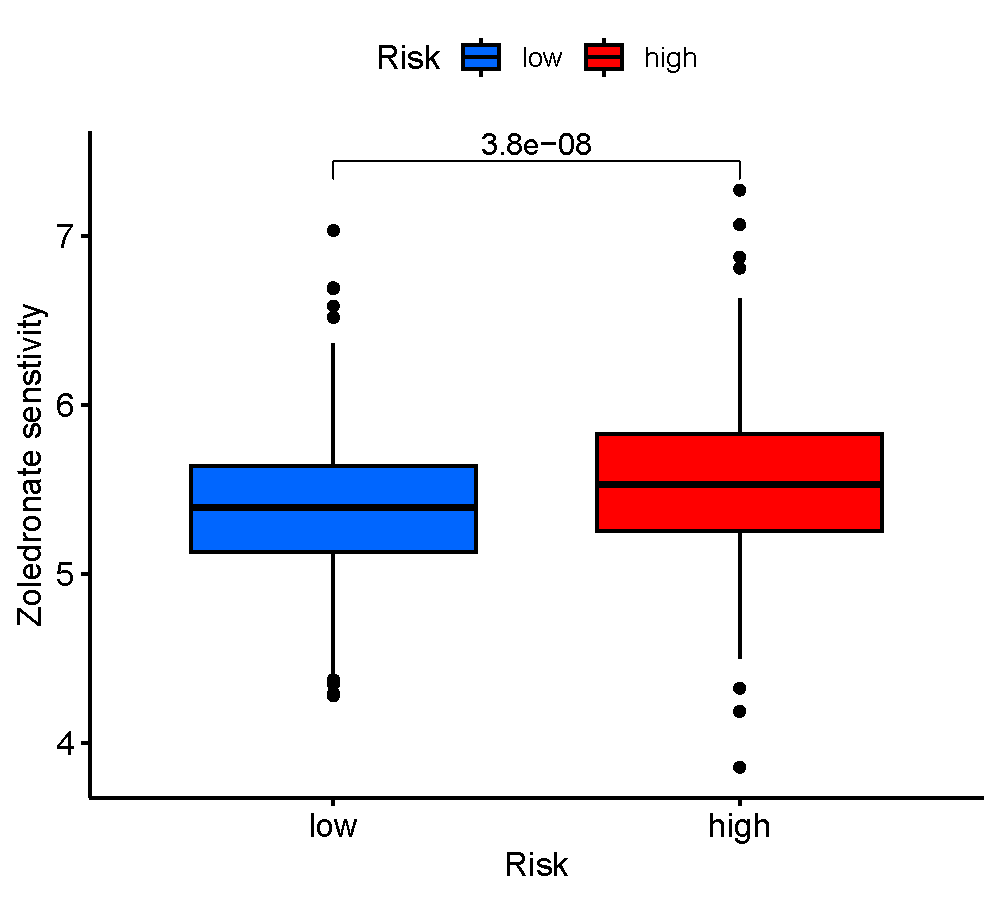

Supplement: Supplementary Figure S1 — Unsupervised clustering of disulfidptosis-related genes and Consensus matrix heatmaps for k = 3-9. [file DataSheet1.zip › Fiugre S5/drugSenstivity.Zoledronate.png]

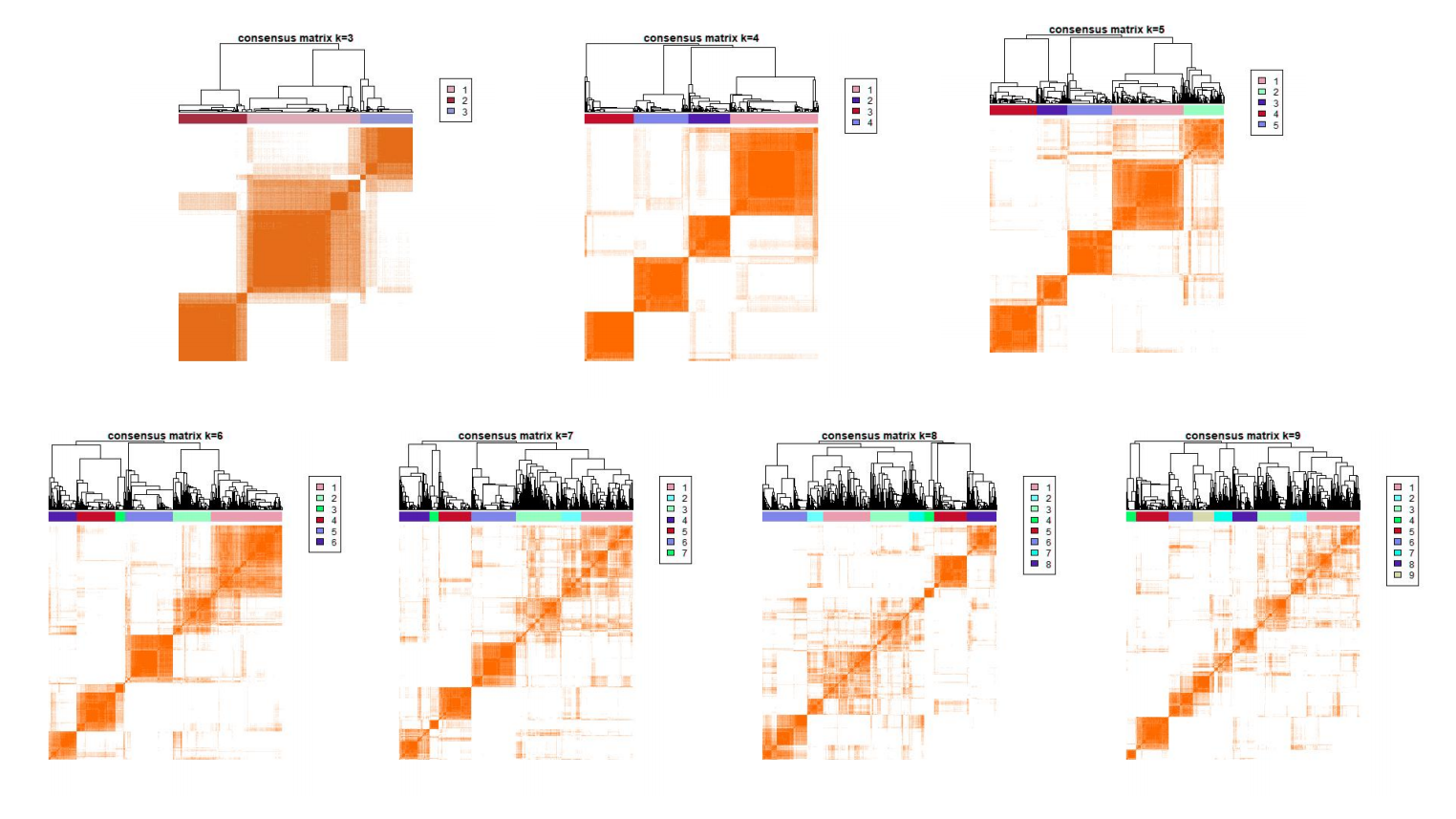

Supplement: Supplementary Figure S1 — Unsupervised clustering of disulfidptosis-related genes and Consensus matrix heatmaps for k = 3-9. [file DataSheet1.zip › Fiugre S1.tif]

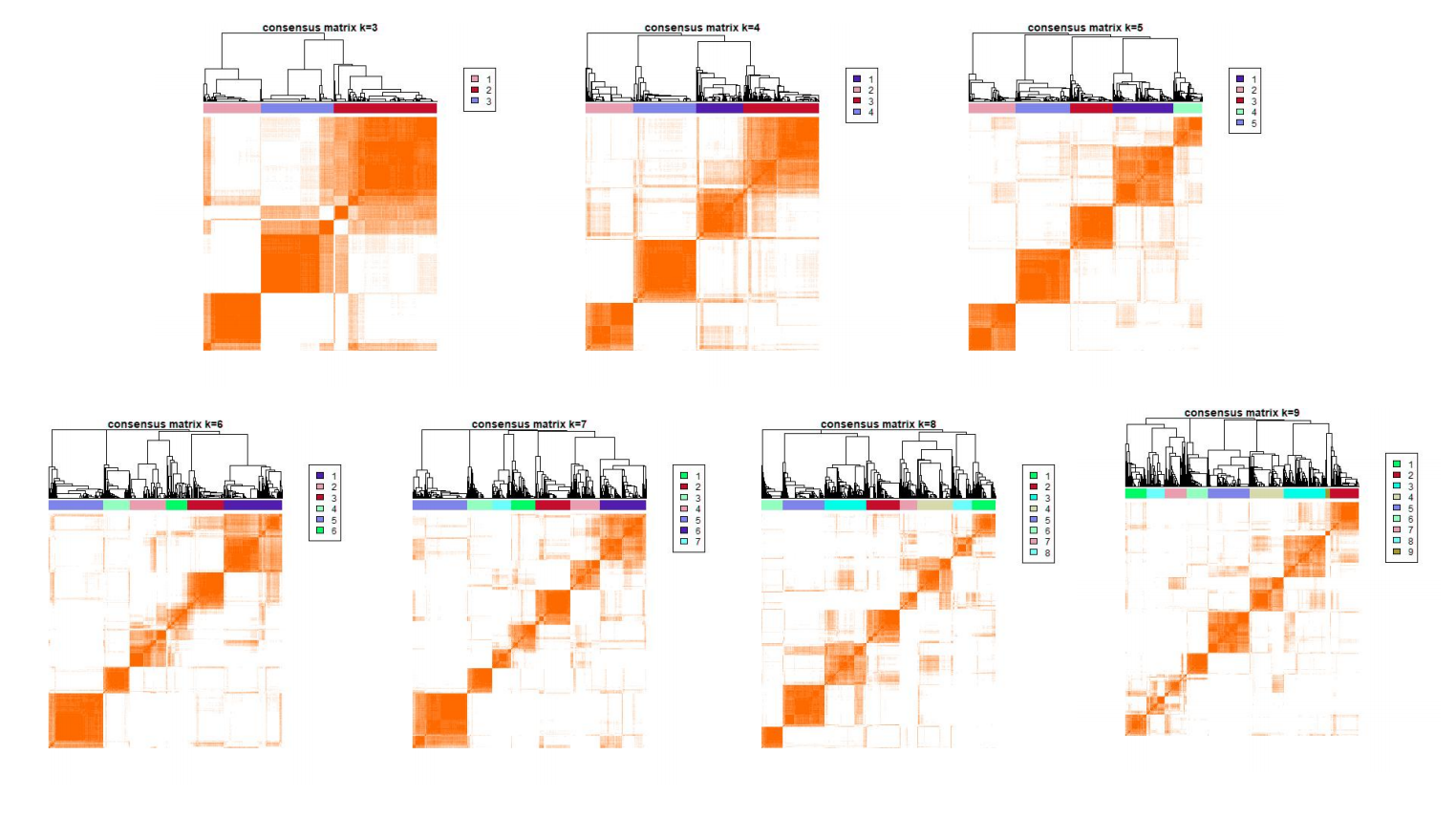

Supplement: Supplementary Figure S1 — Unsupervised clustering of disulfidptosis-related genes and Consensus matrix heatmaps for k = 3-9. [file DataSheet1.zip › Fiugre S2.tif]

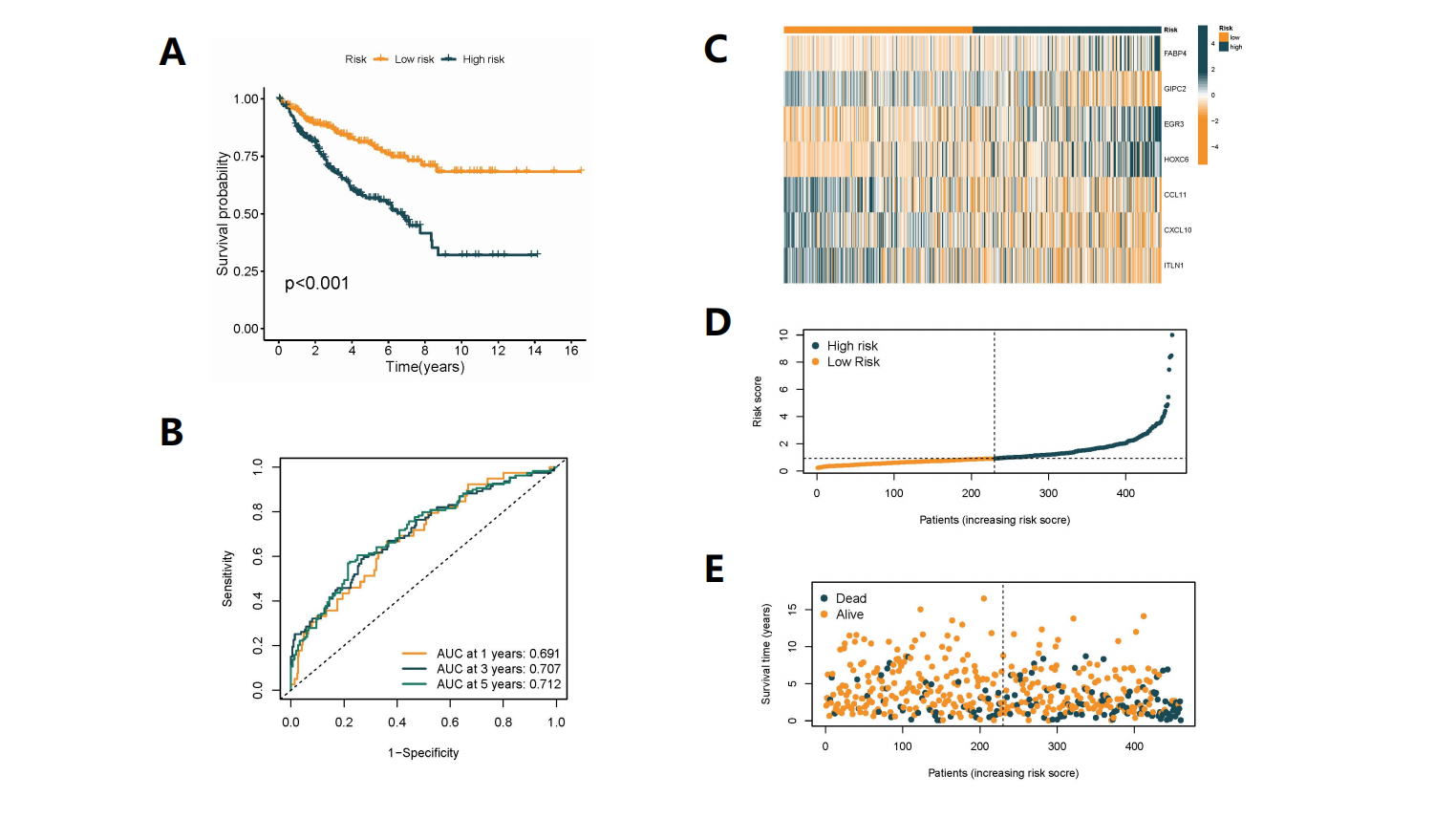

Supplement: Supplementary Figure S1 — Unsupervised clustering of disulfidptosis-related genes and Consensus matrix heatmaps for k = 3-9. [file DataSheet1.zip › Fiugre S3.tif]

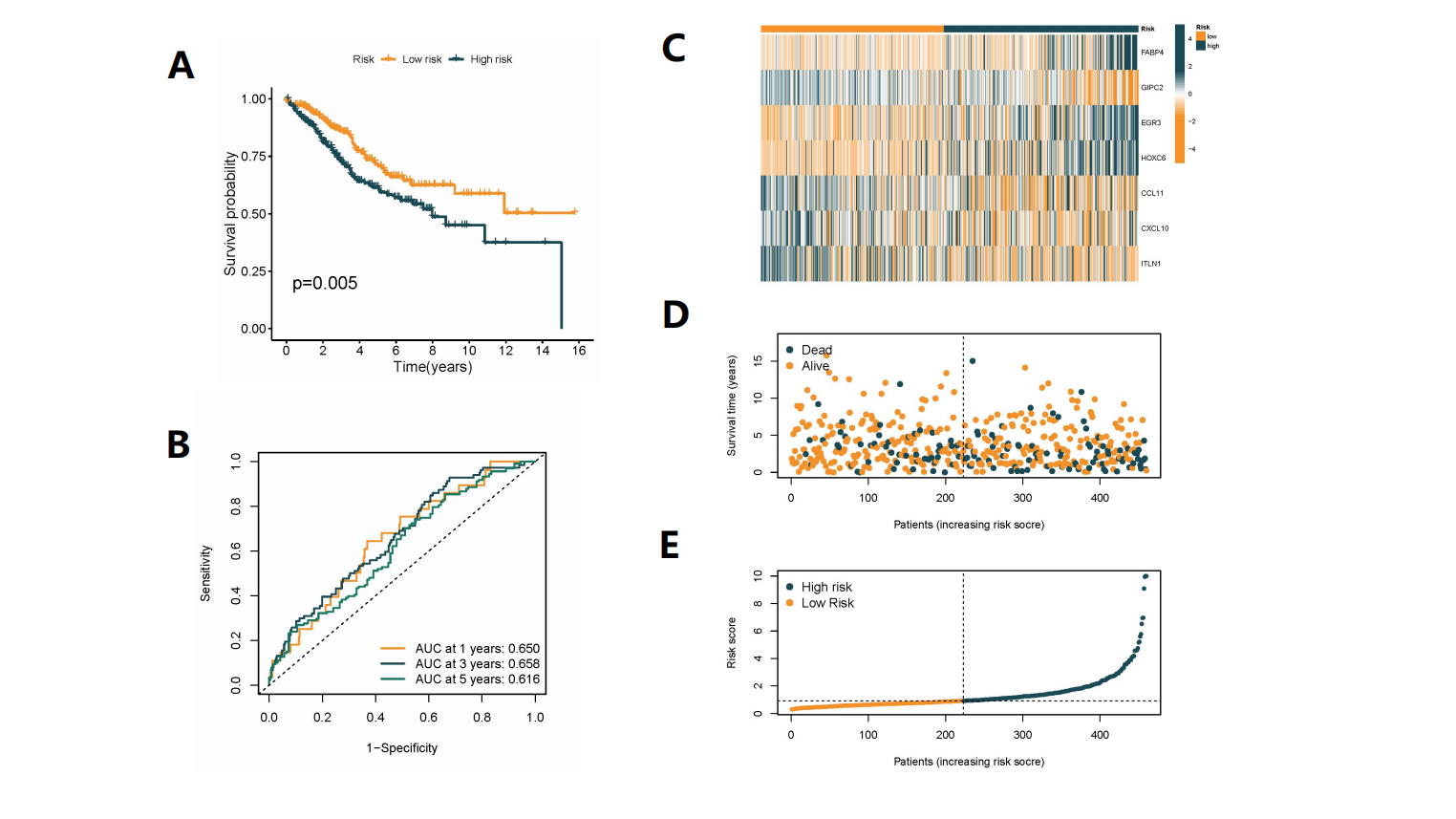

Supplement: Supplementary Figure S1 — Unsupervised clustering of disulfidptosis-related genes and Consensus matrix heatmaps for k = 3-9. [file DataSheet1.zip › Fiugre S4.tif]
